# Post-Miocene tectonics of the Northern Calcareous Alps

**DOI:** 10.1038/s41598-022-22737-5

**Published:** 2022-10-22

**Authors:** Jacek Szczygieł, Ivo Baroň, Rostislav Melichar, Lukas Plan, Ivanka Mitrović-Woodell, Eva Kaminsky, Denis Scholz, Bernhard Grasemann

**Affiliations:** 1 Department of Geology, University of Vienna, Vienna, Austria; 2 Institute of Earth Sciences, University of Silesia, Sosnowiec, Poland; 3 Institute of Rock Structure and Mechanics, The Czech Academy of Sciences, Prague, Czech Republic; 4 Department of Geological Sciences, Faculty of Science, Masaryk University, Brno, Czech Republic; 5 Karst and Cave Group, Natural History Museum, Vienna, Austria; 6 Institute of Soil Physics and Rural Water Management, University of Natural Resources and Life Sciences, Vienna, Austria; 7 Institute for Geosciences, Johannes Gutenberg University Mainz, Mainz, Germany

**Keywords:** Solid Earth sciences, Geodynamics, Geology, Geomorphology, Tectonics

## Abstract

The Late Cretaceous orogeny followed by the Eocene collision of the Adriatic with the European plate dissected the Northern Calcareous Alps (NCA) by a number of well-studied strike-slip fault systems accommodating N-S shortening and E-W stretching. However, the post-Miocene fault activity is poorly constrained due to lack of Neogene faulted sediments, and glacial erosion of geomorphic indicators. Using the protected environment of caves, we fill the knowledge gap in the post-Miocene evolution of the NCA by paleostress analysis of 172 reactivated faults that offset passages in 28 caves near major faults. Constrained maximum age of caves, our results indicate that the NCA have been subjected to N to NE trending compression since Pliocene. Faulted speleothems dated with ^230^Th/U method, indicate that the recorded present-day stress state did not significantly change during the last 0.5 Ma. In contrast to the previously proposed post-Miocene N-S extension of NCA, but in agreement with what was observed in Vienna and Pannonian basins, we conclude that the eastward extrusion resulting from N-S convergence has continued despite a distinct slowdown of plate tectonic velocities in the late Miocene. The N-S extension affected only the Alpine front during Pliocene Molasse basin inversion, while at the scale of the Alpine orogen the NCA underwent successive N-S shortening and E-W stretching.

## Introduction

**Figure 1:**
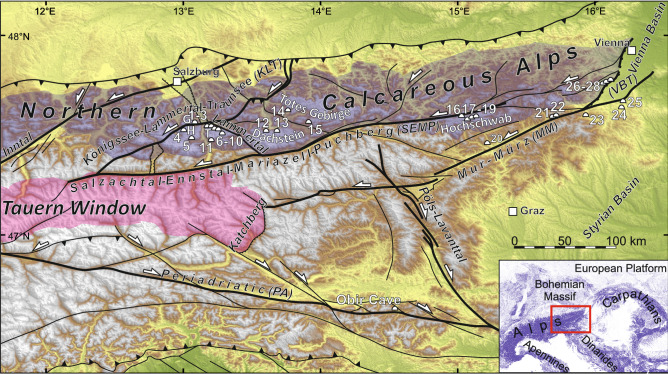
Major fault systems accomodating lateral extrusion of the Eastern Alps [after 1, 2, 6] imposed on Shuttle Radar Topography Mission (SRTM) 1 arc-second shaded relief with digital elevation model (https://www2.jpl.nasa.gov/srtm/; CC BY 4.0 ). Studied caves: 1. Gruberhorn, 2. Gamssteig, 3. Dependance, 4. Interessante, 5. Tantal, 6. Bierloch, 7. Berger, 8. Schneeloch, 9. Felsbrücken, 10. Jack’Daniels, 11. Eisriesenwelt, 12. Hirlatz, 13. Mammut, 14. Kugelmühle, 15. Bullen, 16. POL-Nord-Ponor, 17. Speikboden, 18. Potentialschacht, 19. Hirschgruben, 20. Gr. Offenberger, 21. Zederhaus, 22. Räuber, 23. Hermanns, 24. Excentriques, 25. Altaquelle, 26. Fraisloch, 27. Eisenstein, 28. Emmerberg; *G* Göll, *H* Hagengebirge, *T* Tennengebirge. The map layout was created in QGIS 3.22.4 (https://www.qgis.org/en/site/; GNU GPL).

After the subduction of the Penninic ocean followed by the collision of Adria with Europe in the Eocene, the Eastern Alps experienced an eastward lateral motion of crustal blocks between the sinistral Salzachtal-Ennstal-Mariazell-Puchberg fault (SEMP) to the north, and the dextral Periadriatic Fault (PA) to the south, since the Miocene, generally referred to as lateral extrusion^1–6^ (Fig. 1). The present-day velocity field still reveals the lateral extrusion driven by the N-directed indentation of Adria into Europe^7–10^. The Northern Calcareous Alps (NCA) are dissected by numerous strike-slip fault systems with a wide range of strikes (among which E-W, SW-NE and NW–SE dominate), which accommodated the N-S shortening and E-W stretching of the orogen, recording a complex kinematic evolution with various stages of fault reactivation^3^. However, the timing of these stages in the NCA is poorly constrained by radiometric dating or faulted Neogene intramountain sediments, and there is a considerable lack of knowledge in the post-Miocene tectonic evolution, filled only partly by scattered data on individual fault segments^11,12^. Furthermore, knowledge of the Pleistocene fault activity is very limited in the NCA since potential geomorphic signals of faulting have been erased by glacial and karstic erosion. In contrast, geophysical data consistently showing present-day tectonic motions as closely resembling those of the Miocene, only that one order of magnitude slower^8–10,13^

Karst caves develop along preexisting discontinuities which can be reactivated after the cave is formed^14^, and crucially the caves represent a unique environment where even small-scale tectonic displacements may be preserved and can be used for neotectonic and paleoseismic studies^11,14–18^. Here, we focus on cave passage offsets, which are particularly useful by combining data on reactivated fault kinematics with dated broken and sealed speleothems. We collected 172 kinematic data of reactivated faults from 28 caves, some of which were dated with the ^230^Th/U method, and demonstrate that the central and eastern NCA are affected by a complex pattern of post-Miocene deformation ranging from N-S shortening, orogen-parallel extension and sinistral shearing.

## Post-Miocene tectonics of the Eastern Alps

The Eastern Alps represent an N-propagating thrust wedge composed of the Austroalpine nappe system consisting of a continental basement and cover sequences (Adria)^19,20^. Convergence between the Adriatic and the European plates started in the Late Cretaceous^21^. In the Oligocene/Miocene subduction roll‐back along the Carpathian arc and Pannonian back‐arc basin opening contributed to switching from the collisional N-S shortening to E-W orogen-parallel extension^1–4,22^.

The along-strike extension in the Eastern Alps has been accommodated by the exhumation of the Tauern Window along the W-dipping Brenner and the E-dipping Katschberg normal faults and the Rechnitz Window together with major, strike‐slip faults that fragmented brittle crust into fault-bounded wedges^23,24^. The NCA were divided along the sinistral Inntal, Königssee-Lammertal-Traunsee (KLT) and SEMP faults^25^ (Fig. 1). To the south, the sinistral Mur-Mürz (MM) fault system continues into the Vienna Basin Transfer fault system (VBT) accommodating eastward extrusion^6^ (Fig. 1).

Previous studies were primarily focused on the initiation and the main lateral extrusion phase in the Miocene, with a less well-constrained geologic record of younger phases during the Plio-Pleistocene [^5^ and references cited therein]. Along SEMP and KLT, Late Miocene E-W compression was followed by N-S extension interpreted as a response to the growing topography^2,3^. Fault kinematic analysis and apatite fission track ages suggest a Late Pannonian-Pontian E-W contraction along the MM^6^. The 500 m of uplift, during the last 4 Ma, south from MM was likely to result from renewed N-S compression^26^. In VBT the late Pannonian E-W shortening reactivated originally sinistral faults with dextral kinematics resulting in basin inversions^4^. During the latest Miocene and Pliocene, uplift continued but under N-S compression, which readjusted sinistral kinematics on the NE-SW striking faults^4,27^.

In general, GNSS data suggest the ongoing eastward motion of the central crustal blocks in the Eastern Alps^9,10^. Also, N-S compression, which drives lateral extrusion, is ongoing, as indicated by focal mechanism solutions^28^ and borehole breakout^8,12^. Earthquake focal mechanisms along the MM revealed compression rotating from NNE-SSW in the west, to NE-SW in the east^13^ which is consistent with GNSS based model that estimates 1.4 ± 0.2 mm/a sinistral motion along the MM^10^. However, the geological record of recent fault kinematics in the Eastern Alps is less clear than the geophysical data. Present-day kinematic behavior of active faults recorded in microdisplacements so far did not reveal consistent and/or expected kinematics, and the total displacements are a magnitude smaller than those from the GNSS data^29^. Also, paleoseismic data were based on secondary earthquake effects with no kinematic indicator^15,30^, except for the Hirschgruben cave where Late Pleistocene sinistral slip on faults parallel to SEMP has been documented^11^. Late Pleistocene and Holocene fault reactivation were also recorded in the Obir cave, which has been linked to the PA activity^17^ at the southern boundary of the lateral extrusion (Fig. 1). Outside the Eastern Alps, in the Vienna Basin, the fault ruptures evidence Late Pleistocene-Holocene activity of VBT^31,32^.

## Results

### Paleostress reconstruction from cave offset

**Figure 2:**
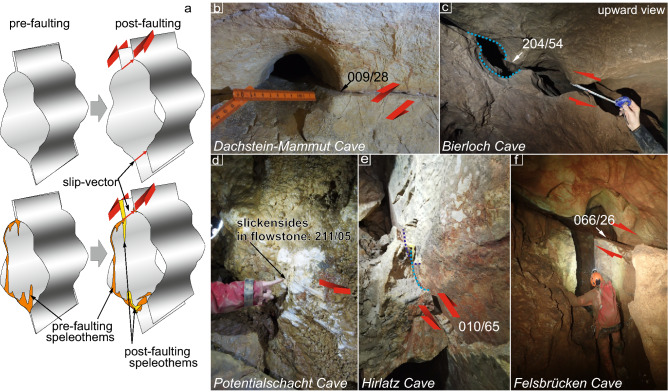
(**a**) – Scheme of cave passage offset along a reactivated fault; (**b–f**) – Field examples of cave passage offsets; for the caves location see Fig. 1.

Displaced karst cave conduits may serve as the geomorphic indicator of movement postdating cave formation. The conduits typically form along pre-existing discontinuities, if the orientation of which is favorable to reactivation with respect to regional stress the reactivation effect may be observed in an offset of karst morphology (Fig. 2). To measure a total offset, only sites with clear pre-faulting morphology and without signs of gravity-induced collapses have been chosen for this study.

**Figure 3:**
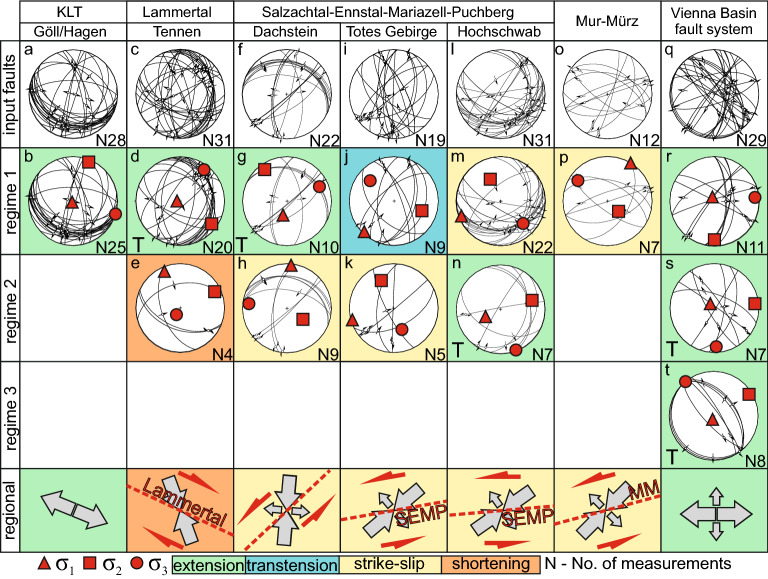
Fault-slip data divided into fault-sets reactivated in different regimes; great circles: blue-normal faults; red—reverse faults; part of faults not matching any of the regimes have been rejected; regional stress is the mean of the computed horizontal compressions or extension and excludes local topographic influences denoted by “T”.

Studied faults often produce or record no slickenlines, hence we used slip vectors revealed from the offsets that are equivalent to the kinematic marker for the paleostress analysis^14^. If cave passages would be geometrically ideal cylindrical tubes, the determination of the slip vectors from offset passages without striations on the faults would be impossible. However, the walls of natural cave passages are characterized by dissolution mesoforms such as scallops, pockets, anastomoses, rills, etc. which, when displaced, are ideal markers to determine the exact slip vector in 3D. We computed paleostress tensors from fault plane orientations, slip vectors, and sense of movement using the multiple inversion method for heterogeneous fault-slip data processed in the MARK2010 software^33^. The resulting stress states are described by the orientation of three orthogonal principal stress vectors σ_1_, σ_2_ and σ_3_ (σ_1_ represents the maximum compressive normal stress; Fig. 3; the raw data are available in supplementary material). The separated regimes do not represent successive tectonic phases, but only the most probable stress tensors associated with particular best-fitting sets of faults reactivated under similar conditions. The studied caves are located in massifs bounded by major faults (Fig. 1). The measured offsets range from 1 to 80 cm.

In the Göll and Hagengebirge massifs adjacent to the KLT, tens of active, mostly oblique normal strike-slip faults were documented. The dominating paleostress regimes are extensional with σ_3_ oriented NW–SE and sub-vertical σ_1_ (Fig. 3b). In Tennengebirge, which is bounded from NNE by the Lammertal fault, a similar extension NNE-SSW was recorded (Fig. 3d), but also the set of faults operated under NNW-SSE σ_1_ (Fig. 3e). The regimes computed for the Dachstein fault sets are similar to KLT, i.e. the extensional regime with σ_1_ steeply inclined to SSW (196/55°) and SSW-trending σ_3_ (Fig. 3g), and the strike-slip one with N-S oriented horizontal σ_1_ (Fig. 3h). To the E, in Totes Gebirge, two regimes have been identified with NE-SW (transtension) and ENE-WSW (strike-slip) oriented σ_1_, and σ_3_ inclined to NW and SSE, respectively, that were calculated from mostly steep oblique reverse NNE to NW striking faults with offsets up to 0.4 m (Fig. 3j,k). In the Hochschwab massif, 80 km to the E where SEMP bends from ENE-WSW to E-W, a NE-SW oblique sinistral strike-slip regime was computed from reverse, oblique reverse, oblique normal, and sinistral strike-slip reactivated faults (Fig. 3m). Yet, some faults have been reactivated under an extensional regime with very steep σ_1_ (Fig. 3n). Outside the NCA, consistent results were obtained from the NNE-SSE, ESE-WNW and ENE-WSW striking faults along the MM, where also the sinistral strike-slip regime is driven by NE-SW compression (Fig. 3p). However, fault kinematics changes dramatically along the southern part of VBT, where NW–SE and NNE-SSW oriented faults were recorded, with normal to sinistral cumulative offsets of a few mm to a couple of cm. Here, we recorded three extensional regimes with vertical σ_1_ and σ_3_, which varies from E-W (Fig. 3r), NW–SE (Fig. 3t) to N-S (Fig. 3s).

### ^230^Th/U dating of deformation

Totally 22 speleothem samples from 6 caves (one fault in each cave) were collected to determine the deformation interval by dating either broken layers pre-dating the faulting or layers covering the fault yielding the post-deformation age (Fig. 2a, Table 1).

**Table 1:** ^230^Th/U dating results for cave speleothems from the Northern Calcareous Alps (Austria).

Area	Sample	Cave	Fault orientation		Dated Offset [cm]	238U [µg/g]	±	232Th [ng/g]	±	(234U/238U)	±	(230Th/238U)	±	Age uncorrected [ka]	±	Age corrected [ka]	±
			Dip dir	Dip ang													
Dachstein	IM6	Mammut	275	65	80	0.130	0.001	1.760	0.019	1.524	0.004	1.379	0.009	198.2	3.4	198.0	3.5
	DK2	Mammut	275	65	80	0.004	0.000	4.462	0.046	1.070	0.037	2.047	0.262	> 500	–	> 500	–
Hochschwab	P2 -A	Potential	118	07	1	0.033	0.000	4.954	0.061	1.046	0.004	1.173	0.021	> 500	–	> 500	–
	P2 -B	Potential	118	07	1	0.044	0.000	254.998	3.322	0.096	1.340	1.011	0.033	114.9	2.9	> 500	–
	P5	Potential	118	07	1	0.022	0.000	6.548	0.179	2.194	0.052	0.093	0.041	8.75	0.81	4.7	2.1
	P16-3	Potential	118	07	1	0.025	0.000	16.091	0.186	1.070	0.008	1.166	0.023	> 500	–	> 500	–
	S1-A	Speikboden	183	34	1	0.022	0.000	below detection limit	below detection limit	1.302	0.010	1.358	0.023	363.87	+ 53.19−36.01	363.87	+ 53.19−36.01
	S1-B	Speikboden	183	34	1	0.016	0.000	13.153	0.137	2.277	0.174	0.877	0.033	61.8	2.0	50.6	+ 5.88−4.93
Vienna basin	EM1A	Emmerberg	342	86	Post 3.9/pre 0.5	0.047	0.000	122.683	1.597	1.715	0.771	0.907	0.119	157.5	+ 7.57−7.02	77.2	+ 126.20−31.80
	EM1B	Emmerberg	342	86	3.9	0.153	0.001	246.048	3.218	1.602	0.221	1.276	0.103	183.6	4.4	148.9	+ 73.2−36.9
	EM1C	Emmerberg	342	86	3.9	0.213	0.002	51.355	0.589	1.493	0.018	1.405	0.017	224.8	5.2	221.0	+ 11.98−10.62
	EM2A	Emmerberg	342	86	0.5	0.033	0.000	4.579	0.053	1.262	0.009	0.105	0.019	12.66	0.69	9.45	1.8
	EM2B	Emmerberg	342	86	0.5	0.143	0.001	32.656	0.360	1.396	0.014	1.044	0.010	140.5	2.6	136.2	3.7
	EM2016-2	Emmerberg	342	86	3.9	0.366	0.002	4.363	0.045	1.258	0.002	1.315	0.007	392.22	+ 18.2−15.7	392.0	+ 17.70−15.50
	EH1a	Eisenstein	090	70	Co-seismic	0.097	0.001	0.221	0.004	1.164	0.002	0.817	0.008	126.5	2.3	126.4	2.3
	EH1b	Eisenstein	090	70	Co-seismic	0.073	0.000	0.128	0.006	1.153	0.003	0.798	0.013	123.7	3.6	123.7	3.6
	EH2	Eisenstein	090	70	Post-event	0.059	0.000	0.057	0.001	1.406	0.005	0.111	0.003	8.94	0.22	8.92	0.22
	EX1	Excentriques	122	88	Pre- and Post-dating	0.398	0.004	6.655	0.072	1.141	0.008	0.244	0.003	26.60	0.38	26.18	0.42
	EX2	Excentriques	122	88	Post-dating	0.399	0.003	1.320	0.014	1.230	0.003	1.135	0.006	231.0	4.3	231.0	4.3

**Figure 4:**
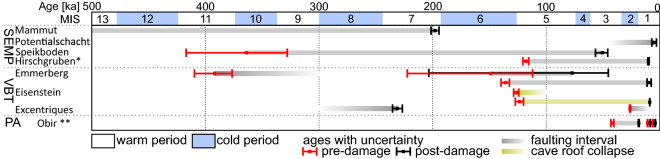
Age distribution of damaged speleothems in studied caves against the marine isotope stages (MIS); for caves location see Fig. 1; *^11^; **^17^.

Not all faults observed in caves are associated with damaged speleothems and therefore our chronological data capture only a subset of the presented fault-slip data (Fig. 3). In Mammut Cave on Dachstein, a normal sinistral fault 283/70° with striae 200/04° continuously transformed into sinistral striae 242/59° of cumulative offset to ~ 0.8 m. Fault fiber crystals (DK2), related to this reactivation revealed an age beyond the limit of ^230^Th/U dating (i.e. 0.5 Ma), Thus, the maximum age of the fault reactivation is that of the Miocene-Pliocene transition^31^. While the flowstone healing the fault plane (IM6) postdating the faulting grew 198 ± 4 ka ago. Similarly, in Potentialschacht, Hochschwab, only 4.7 ± 2.1 ka old flowstone (P5) postdating the 139/32° oriented reverse fault with an offset up to 7 cm, while broken flowstones predating faulting (P2-A, P2-b, P16-3) yield ages > 0.5 Ma. In the nearby Speikboden Cave, the E-W striking fault revealed a relatively older (undated) reverse phase and a younger normal sinistral one for about 1–3 cm between ${364}_{-36}^{+53}$(S1-A) and ${51}_{-5}^{+6}$ ka (S1-B; Table 1, Fig. 4).

Chronological data from flowstone-rich caves along the Vienna Basin are more robust. The older faulting phase is constrained by faulted flowstone from Emmerberg cave yielding an age of ${392}_{-16}^{+18}$ ka (EM2016-2) and flowstone covering collapse in Excentriques Cave dated to 231 ± 4 ka (EX2), which may represent a minimum age of faulting. The younger faulting has been dated in three caves. In Emmerberg Cave, faulted flowstone yields ages ${221}_{-10}^{+12}$(EM1C; oldest broken layer) to ${149}_{-37}^{+73}$ (EM1B; youngest broken layer), which points to maximum reactivation timing, while the layer enveloping broken flowstone was dated to ${77}_{-32}^{+126}$ ka (EM1A). In Emmerberg Cave the faulted layer is 136 ± 4 ka old (EM2B) and it is covered with 9.5 ± 1.8 ka old (EM2A) calcite. In Excentriques Cave, the younger phase is constrained by fractured flowstone dated to 26.2 ± 0.4 ka (EX1). In Eisenstein Cave, no faulted speleothems were found, however abundant of broken and fallen speleothems and proximity to Emmerberg Cave, together with similar ages of fracturing allow correlation of the deformations from both caves. Top layers of fallen stalagmite predating collapse yielding ages of 126 ± 2 (EH1a) and 124 ± 4 ka (EH1b), and the stalagmite that grew on collapse deposit is 8.9 ± 0.2 ka old (Fig. 4).

## Discussion

### Timing of fault reactivation

Our ^230^Th/U dating results show two faulting phases along SEMP and VBT. The older phase was recorded in Speikboden Cave between ca. 364 and 51 ka, and in Emmerberg Cave where faulted flowstone yielded an age of ${392}_{-16}^{+18}$ ka, which may be constrained by flowstone covering collapse in Excentriques Cave dated to 231 ± 4 ka. The deformation linked with the younger phase has been found in Potentialschacht Cave and all three caves near VBT, and they correlate with the faulting recorded in Hirschgruben Cave about 118 to 9 ka ago^11^. However, deformed flowstone, dated to 26.2 ± 0.4 ka, from Excentriques cave may narrow this interval, or suggest another, younger event (Fig. 4), which would be also in agreement with the latest Pleistocene co-seismic soft-sediment deformations from Hirlatz Cave^15^.

The maximum age of fault slip with no associated damaged speleothems may be constrained by the age of cave formation. Paleophreatic caves within the MM and VBT fault zone are located < 130 m above the valley bottom. The Quaternary incision along the Mur river catchment has been estimated at 40 m/Ma^26^. Hence, the studied caves are most likely of the latest Pliocene/Quaternary age. A similar estimate can be applied to the caves in the NCA, where caves located below 1200 m a.s.l (e.g. Berger, Bierloch, Dependance, and part of Hirlatz cave) likely formed in Quaternary, as inferred from incision rates (120–210 m/Ma)^34^. This incision rate also suggests that the caves we studied above this altitude most probably originated in the latest Miocene and Pliocene.

So far there is not enough data from caves to unequivocally state whether the displacements are co-seismic or creep or a mixture of both. The present-day kinematic behavior of active faults in the Eastern Alps reveals a variety of different displacement modes at the micrometer level^29^ and associated near-surface crustal stress variations^35^ due to interplaying tectonic crustal processes and gravitational relaxation^36^. The macroscopic observation shows mm to cm-scale present-day co-seismic displacements resulting in speleothems damage, as observed after the 2017 Mw 6.6 Bodrum–Kos earthquake in Greece^18^ or in Obir Cave where the 1976 Mw 6.7 Friuli earthquake dislocated dripstone column^17^. A co-seismic origin of the deformations is also supported by the soft-sediments deformation structures that were investigated in Hirlatz Cave^15^ where we documented a reverse fault (Fig. 2e). Also several cm-long linear strike-slip scratches on cave walls are the argument for co-seismic deformations, as in the Hirschgruben Cave^11^ or Potentialschacht Cave (Fig. 2d). Moreover, in a few caves, e.g. Emmerberg or Speikboden, we observed at least two reactivations of the same fault. A similar observation has been recorded in Obir Cave^17^ (Fig. 1) or in the Demänovská Cave in the Carpathians^16^. This would indicate repeated activation of the same faults to accommodate stressdrop and abrupt displacements rather than creep. Thus, we argue that the macroscopic offsets are co-seismic and the total offsets are the results of cumulative slip events, although we are aware that it is impossible to estimate the number of events in between dated pre- and post-faulting speleothems. Therefore, it is impossible to calculate slip rates from faults where both pre-faulting and post-faulting layers were dated.

### Fault reactivation causes

Cave passages are voids in the mountains, which affect the near-field stress state in the wall rocks, comparable to mines or tunnels. However, the water dissolves the carbonates at much lower rates compared to artificial excavations resulting in several orders of magnitudes less stress concentrations^37,38^. An offset of cave passages along faults are rare observations, where faults have been reactivated by tectonic (or gravitational processes) but in general, the passages are not offset ruling out that this is a common process of local stress relaxation due to cave formation. If the karstic morphology is not in a static equilibrium state with the wall rocks, the stress pattern known as tension dome leads to collapses until a cave obtains an arch-like cross-section, which is the third common morphology type in caves^37^. Different from artificial constructions, cave formation is too slow to induce seismicity^39^, since for a dissolution of a passage in carbonate rocks with a cross-section diameter of 3 m, a time span of at least 10,000–100,000 years is required^40^. In contrast, numerous studies have shown for recent and historical earthquakes (e.g. ^17,18^) as well as for slow-rate tectonic movements that faults can dislocate cave passages (e.g. ^29,41^).

As some of the studied caves are located above valley bottoms, gravitationally induced fault reactivation due to the topography should be considered. Especially dip-slip faults located close to the slope may be affected by gravity (see sets with “T” in Fig. 3). The difference between gravitational and tectonic movements is evident in the Tennengebirge. Here, the NE-directed slip toward the Lammertal valley along E dipping bedding planes (Fig. 2f) most likely indicates gravitational movement. Whereas, in the same massif, E-W striking dextral-reverse faults, so parallel to the valley and Lammertal Fault (Fig. 2c) operated under NNW-SSE compression, so perpendicular to the valley axis, which we interpret to be tectonically driven.

Although we cannot explicitly exclude local variations of the stress field due to topography^42^, most faults have been reactivated by horizontal compression that we attribute to tectonic forces. The tectonic reactivation is also supported by slickensides observed on some fault planes (Fig. 2d,e). The studied cave offsets occurred at already preexisting fractures with significantly reduced strength parameters, i.e. the angle of internal friction and the cohesion (*φ, c*) and the measured fault data do not indicate simple conjugate sets typical for newly formed faults (Fig. 3). As the Mohr–Coulomb theory suggests that shear fracture formation with respect to the principal stress directions strongly depends on the host rock *c* and *φ*, the fault reactivation is mainly controlled by frictional resistance on pre-existing surfaces. Therefore, under the same stress regime, a much wider range of spatial orientations of weak faults with more acute and/or obtuse angles to the principal shear stress might reactivate compared to the conjugate orientations of newly formed fractures predicted by Mohr–Coulomb theory^43,44^.

**Figure 5:**
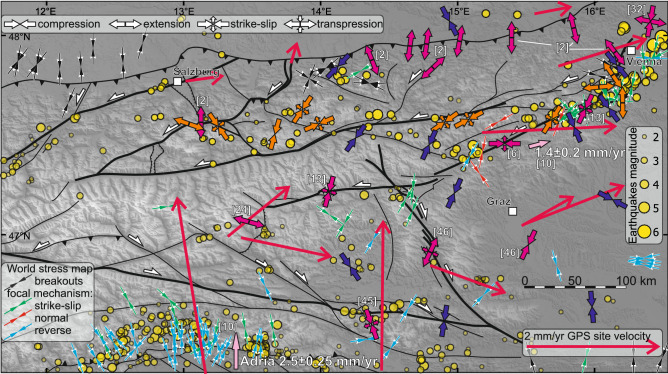
Major fault systems in the Eastern Alps [after 1, 2, 6] imposed on Shuttle Radar Topography Mission (SRTM) 1 arc-second shaded relief with digital elevation model (https://www2.jpl.nasa.gov/srtm/; CC BY 4.0 ) with: Pliocene–Pleistocene paleostress data (orange arrows—this research, purple arrows—previously published data, numbers in square brackets by the arrows correspond with the references list), present-day maximum horizontal compressional stress S_Hmax_ (dark blue arrows ^8^ and World Stress Map ^7^), Present-day maximum uniform principal strain axes and relative displacements (pink arrows ^10^), GPS site velocity (scaled red arrows ^10^), seismicity (01.01.2000–18.07.2022 https://www.usgs.gov/products/data-and-tools/real-time-data/earthquakes). The map layout was created in QGIS 3.22.4 (https://www.qgis.org/en/site/; GNU GPL).

Considering the small offsets of the measured faults, that are subsidiary to the major fault systems, their kinematics and derived principal paleostress directions reflect the local stress field associated with the major fault systems (Fig. 5). The faults in Göll and Hagengebirge may be interpreted as extensional relay along the KLT. In the Tennegebirge we recorded dextral shear along the Lammertal fault, which is robustly represented by the Bierloch Cave fault (Fig. 2c) where dextral slip along SSW dipping faults, parallel to the Lammertal fault and located only 1.5 km from its core, shows the tectonic origin of this movement linked to a major fault. Since Dachstein is distant from the KLT and SEMP, the fault orientation (Fig. 3), together with the prevalent reverse sense (e.g. Fig. 2e), indicates N-S shortening. Further to the East, NNE compression in relation to the central and eastern segments of SEMP (in Totes Gebirge and Hochschwab), as well as MM, caused sinistral shearing along the faults. The W-E extension (Fig. 3r) along the margins of the Vienna Basin agrees with the pull-apart basin opening mode along the VBT. The multiple extensional deformations at the boundary of the NCA and the Vienna Basin are in agreement with fault geometry observed in the Quaternary basin fill^45^. The overall changes in the orientation of the principal paleostress directions of the investigated faults systems directly reflect the post-Miocene extrusion tectonics: The western segment of NCA, N of Tauern Window, is dominated by N-S shortening reflecting indentation of the Adriatic Microplate^28^. The further E, the stronger the influence of the extrusion between the SEMP and PA, reflected by the NE trending principal compressive stress direction.

Signals of Pliocene continuation of Adria indentation have been documented in the Eastern Alps (Fig. 5) mainly by indirect observations and less frequently by faults. The youngest apatite (of ca. 3–4 Ma) from the Tauern Window combined with the fault-slip analysis suggests that the Brenner and the Katschberg faults have been active at least up to Pliocene and operated under orogen parallel E-W extension^24^. Also, Pliocene extrusion tectonics is well documented south of MM, along PA^46^, Lavanttal Fault^47^, or in Neogene basins e.g. Styrian Basin^47,48^ or Vienna Basin [^32^ and references there]. In the wider framework, Pliocene deformations driven by the Adria indentation as the far-field effect have been found in Pannonian Basin^49^ and the Western Carpathians^16,50^. Yet, in NCA Plio-Quaternary has been linked with N-S extension, and driven by topographic readjustment^2,3^. In contrast, our results show that NCA have been subjected to the N to NE trended compression since Pliocene with the phase of Middle to Late Pleistocene fault reactivation decently constrained by ^230^Th/U dating. The stresses we have reconstructed are in agreement with the present-day deformations, inferred from both GNSS displacement directions and focal mechanisms (Fig. 5)^7–10^. Thanks to the wide range of ^230^Th/U ages that we provide we can conclude that the currently recorded stress state lasts from at least 0.5 Ma. In a rigid NCA block, in the near-surface zone, the compression is accommodated repetitively by the same reactivated faults. It is indicated by several tens of centimeters offsets documented in the caves, which compared to the currently observed, coseismic displacement of centimeters scale suggests repetitive reactivation. Given the documented horizontal compression toward the N and NE lasting at least since the Middle Pleistocene, the N-S extension tended to affect only the Alpine front itself and was a response to Pliocene Molasse basin inversion and uplift^51^. On the orogen scale, on the other hand, the rigid NCA block, fixed between stable Europe, Inntal Fault, KLT, SEMP, and MM^49^ underwent successive N-S shortening and E-W stretching, as occurred during the Pliocene in the Taurn Window^24^. As shown by numerical modeling, E-W extension during continental convergence in the Eastern Alps is only plausible with the SEMP crustal-scale fault^52^. Thus, if this extension is documented for Pliocene in the Taurn Window, then the SEMP and consequently the adjacent NCA must also have undergone this deformation. The structures observed in caves provide firm evidence of this deformation from at least the middle Pleistocene.

## Conclusions

Our results indicate that NCA have been subjected to the N to NE trended compression since Middle Pleistocene, as pointed by ^230^Th/U dating, and possibly since Pliocene, as inferred from the maximum ages of caves. We provide tectonic and geochronological records for the Plio-Pleistocene kinematics showing (i) the N-S shortening N of the Tauern Window, reflecting indentation, (ii) the sinistral strike-slip tectonics close to the SEMP and MM, reflecting extrusion of the Eastern Alps, and (iii) continuous opening of the southern part of the Vienna Basin. Recorded extensional displacements we associate with mass movements rather than a gravitational collapse of the orogen. In contradiction to the previously proposed post-Miocene N-S extension of NCA^2,3^, and in agreement with what was observed in Vienna Basin^32,45^ and Pannonian Basin^13,49^ we conclude that the eastward extrusion resulting from N-S convergence has continued despite a distinct slowdown in the Late Miocene. The N-S extension affected only the Alpine front itself during Pliocene Molasse basin inversion, while in the orogen scale NCA underwent successive N-S to NE-SW shortening and E-W stretching, which has been an effect of ongoing convergence resulting in Pliocene Taurn Window E-W extension and the near and far tectonic consequences resulting from this key deformation of the Eastern Alps.

## Methods

### ^230^Th/U-dating

^230^Th/U-dating was performed at the Max Planck Institute for Chemistry, Mainz, Germany, with a Nu Plasma multi-collector inductively coupled plasma mass-spectrometer (MC-ICPMS). The weighed samples were dissolved in 7 N HNO_3_, and a mixed ^229^Th–^233^U–^236^U spike was added (see^53^, for details on spike calibration). Potential organic material was removed from the samples by adding a mixture of concentrated HNO_3_, HCl and H_2_O_2_. The dried samples were then dissolved in 6 N HCl, and U and Th were separated using ion exchange columns^54^. For technical details about the MC-ICPMS procedures, see^55^. All activity ratios were calculated using the decay constants of Cheng et al.^56^ and corrected for detrital contamination assuming a ^232^Th/^238^U weight ratio of 3.8 for the detritus and ^230^Th, ^234^U and ^238^U in secular equilibrium.

### Paleostress analysis

We computed paleostress tensors from fault plane orientations, slip vectors, and sense of movement based on the Wallace-Bott hypothesis^57^ that the direction of striation on a fault surface corresponds to the direction of the shear stress on this surface, and thus the shear stress in the perpendicular direction is equal to zero. Using this idea, it was possible to derive a system of homogeneous linear equations for the direct calculation of the reduced paleostress tensor from four homogeneous fault-slip data^58^. Subsequently, the development of the mentioned idea led to the formulation of a geometric interpretation of σ-space in 6D, which was supplemented with a method for the best-fit calculation of the paleostress for a multimember set of homogeneous fault-slip data^59^. In this multidimensional σ-space, individual fault-slip data are represented by individual vectors, and the correct solution is represented by a vector perpendicular to all of them. Since the stress tensor has nine components, even though three pairs are identical, the extension to the 9D space used in this research shows the true geometric relationships^33^. Meanwhile, a multiple inverse method for processing heterogeneous files using a method for best-fit calculation was developed^60^ using all combinations of four- or five-member sets of fault-slip data selected from the heterogeneous data. This method thus made it possible to replace the various total search methods by direct calculation of paleostress from heterogeneous data. However, simple automation of separation into homogeneous sets is not possible for a number of limiting constraints^33^.

We employed the multiple inversion method using the direct calculation for all combinations of the four fault-slip data^58^ to find possible candidates for the correct paleostress tensors using our own Mark2010 software^33^. Since the incorrect inversion of combinations of four fault-slip data are generally scattered and the correct solutions cluster around the same direction^60^, the directions with the maximum density of correct solutions were searched using Watson’s density function extended to 9D space [^33^ and references there]. Subsequently, the angular deviation in σ-space was determined for each fault-slip data and, based on the statistical distribution of the deviation, a demarcation between individual homogeneous sets was sought as a gap and after that, the sets were separated. Finally, paleostress characteristics were calculated from the separated homogeneous sets (crosses) and dispersion in direction is shown by Watson’s density function in 3D for each of the principal stresses.

Before calculation, all fault-slip data were orthogonalized (i.e. corrected so that the normal to the fault surface is perpendicular to the striation) so that the detected deviation was equally distributed over the fault surface orientation and striae direction, but the original data are presented in the diagrams. Data with high deviations were excluded from the calculation.

## Supplementary Information

Supplementary Information.

## Data Availability

All the data for sample dating are reported in Table 1. Fault-slip data, sample location, and their geomorphological context are available in the Supplemental Material.
