# Supplementary material for: Post-Miocene tectonics of the Northern Calcareous Alps
Source: Sci Rep. 2022 Oct 22;12:17730. doi: 10.1038/s41598-022-22737-5 (PMC9588011; doi:10.1038/s41598-022-22737-5)
Supplement: Supplementary file 1 — Supplementary Information. [file 41598_2022_22737_MOESM1_ESM.pdf]

Supplementary information to “Post-Miocene tectonics of the Northern Calcareous Alps (Austria)”

Jacek Szczygieł [1,2], Ivo Baroň[3], Rostislav Melichar[4], Lukas Plan[5], Ivanka Mitrović-Woodell[1], Eva Kaminsky[6], Denis Scholz[7], Bernhard Grasemann[1]

[1] *Department of Geology, University of Vienna, Vienna, Austria; jacek.szczygiel@us.edu.pl*

[2] *Institute of Earth Sciences, University of Silesia, Sosnowiec, Poland*

[3] *Institute of Rock Structure and Mechanics, The Czech Academy of Sciences, Prague, Czech Republic*

[4] *Department of Geological Sciences, Faculty of Science, Masaryk University, Brno, Czech Republic*

[5] *Karst and Cave Group, Natural History Museum, Vienna, Austria*

[6] *Institute of Soil Physics and Rural Water Management, University of Natural Resources and Life Sciences, Vienna, Austria*

[7] *Institute for Geosciences, Johannes Gutenberg University Mainz, Mainz, Germany*

DESCRIPTION OF THE CAVES AND SAMPLE SITES

The following caves and sample sites are documented. The numbering of the caves refers to Fig. 1 in the main text. For most caves, an example photo of the fault is presented. Sites, where samples for $^{230}Th/U$ dating were taken are documented with photographs and maps. Topographic maps are based on the Open Street Maps. All cave maps are taken with permission from the cave cadastre of the Speleologic Society of Vienna and Lower Austria (Landesverein für Höhlnekunde und Wien und Niederösterreich). If the caption lacks the author of the photo, it means that it was taken by one of the co-authors.

1. *Gruberhornhöhle*

No. of Austrian cave register:	1336/29	*Province*	Salzburg
Location	E of Hoher Göll, SSE of Gruberhorn	*Elevation of main entrance [m a.s.l.]*	1,905
UTM 33T Easting	357,642	*UTM 33T Northing*	5, 271,735
Length [m]	6,700	*Depth [m]*	854
Lithology	Dachsteinkalk (Upper Triassic)	*Key reference*	Klappacher & Knapczyk, 1979

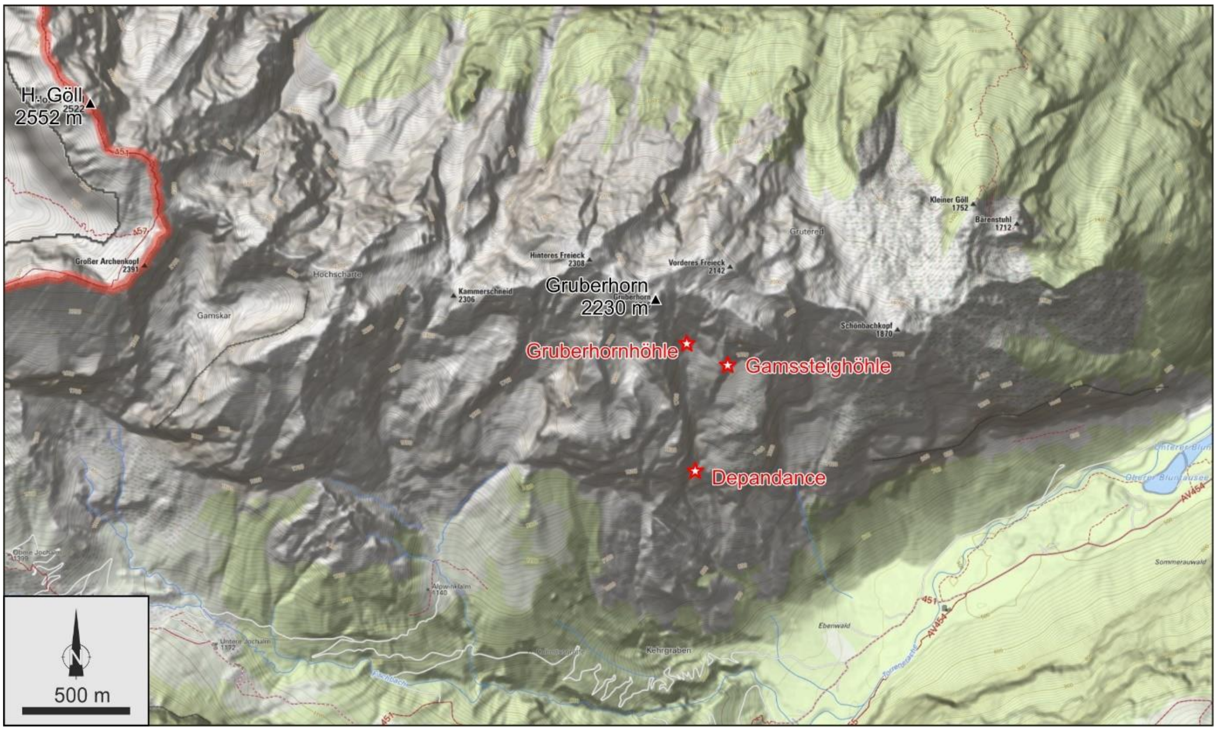

Fig. 1. Topographic map with the location of the investigated cave in Hoher Göll.

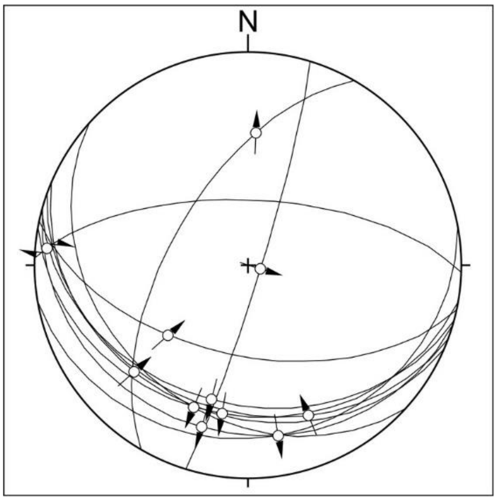

Fig. 2. Angelier diagram of fault-slip data (Equal-area stereographic projection, lower hemisphere) of reactivated faults from Gruberhornhöhle.

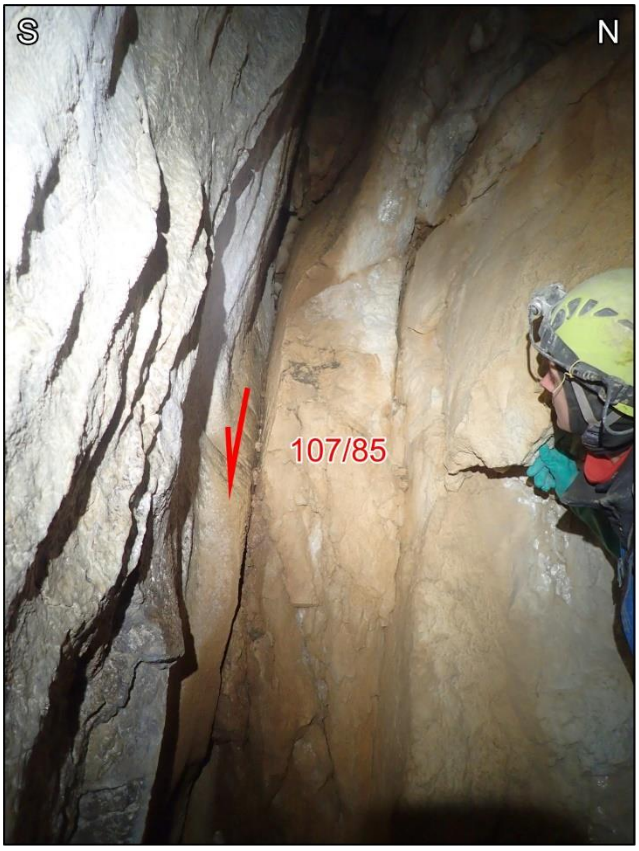

Fig. 3. Photograph of the reactivated fault in Gruberhornhöhle.

2. Gamssteighöhle

No. of Austrian cave register:	1336/48	*Province*	Salzburg
Location	E of Hoher Göll, SE of Gruberhorn	*Elevation of main entrance [m a.s.l.]*	1,905
UTM 33T Easting	357,855	*UTM 33T Northing*	5, 271,643
Length [m]	5,953	*Depth [m]*	860
Lithology	Dachsteinkalk (Upper Triassic)	*Key reference*	Golicz, 2021

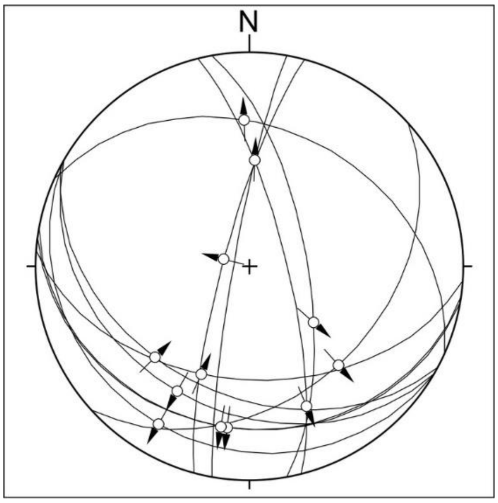

Fig. 4. Angelier diagram of fault-slip data (Equal-area stereographic projection, lower hemisphere) of reactivated faults from Gamssteighöhle.

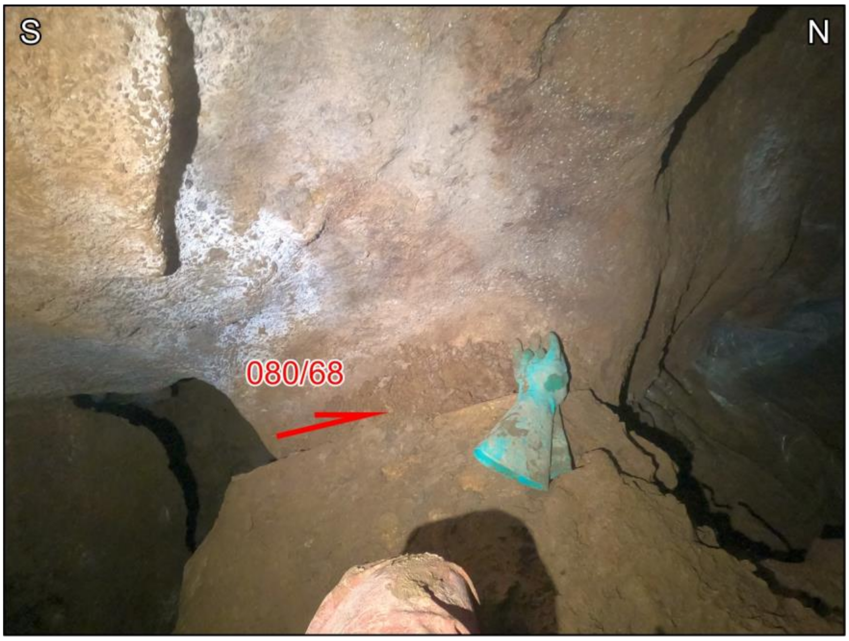

Fig. 5. Photograph of the reactivated fault in Gamssteighöhle.

3. Dependance

No. of Austrian cave register:	1336/45	*Province*	Salzburg
Location	E of Hoher Göll, S of Gruberhorn	*Elevation of main entrance [m a.s.l.]*	1,300
UTM 33T Easting	357,665	*UTM 33T Northing*	5, 271,105
Length [m]	992	*Depth [m]*	289
Lithology	Dachsteinkalk (Upper Triassic)	*Key reference*	Golicz, 2021

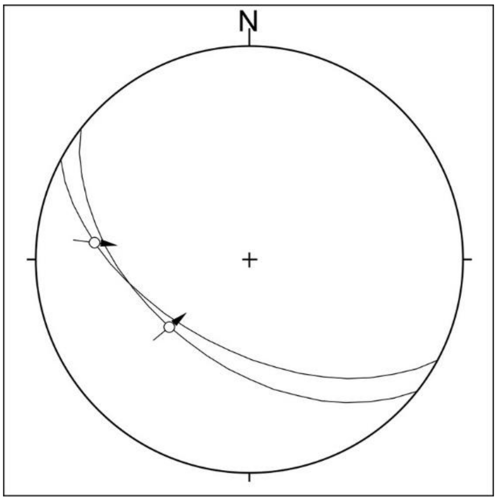

Fig. 1: Angelier diagram of fault-slip data (Equal-area stereographic projection, lower hemisphere) of reactivated faults from Dependance.

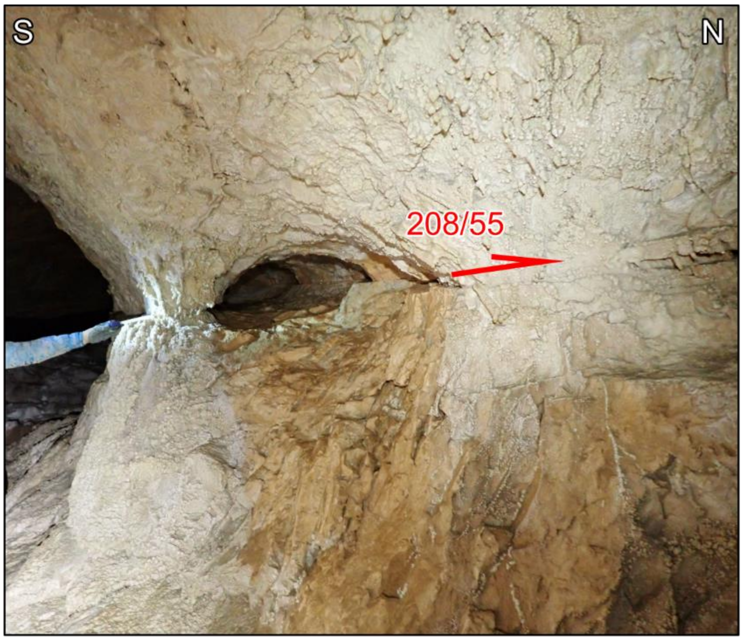

Fig. 2: Photograph of the reactivated fault in Dependance.

4. Interessante Höhle

No. of Austrian cave register:	1335/495	*Province*	Salzburg
Location	Hagengebirge S, E of Kahlersberg	*Elevation of main entrance [m a.s.l.]*	1,940
UTM 33T Easting	353,231	*UTM 33T Northing*	5, 266,264
Length [m]	22,416	*Depth [m]*	639
Lithology	Dachsteinkalk (Upper Triassic)	*Key reference*	Wierzbowski, 2021

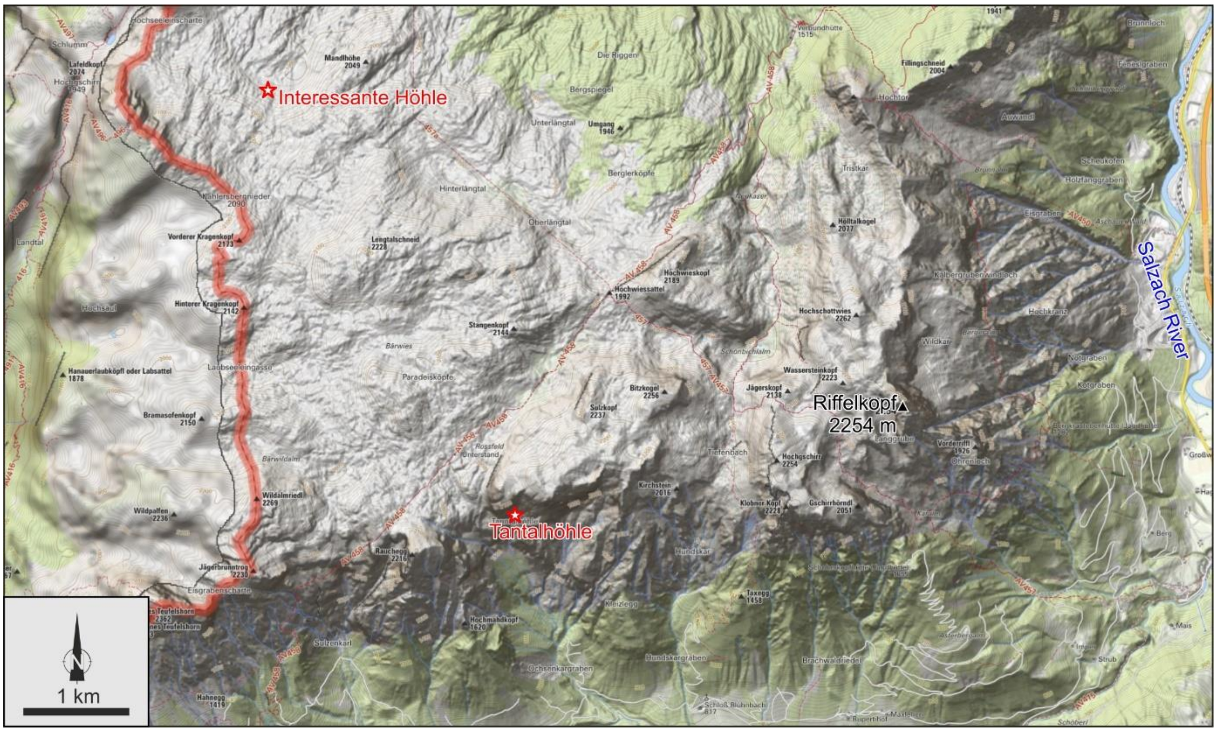

Fig. 8. Topographic map with the location of the caves in Hagengebirge.

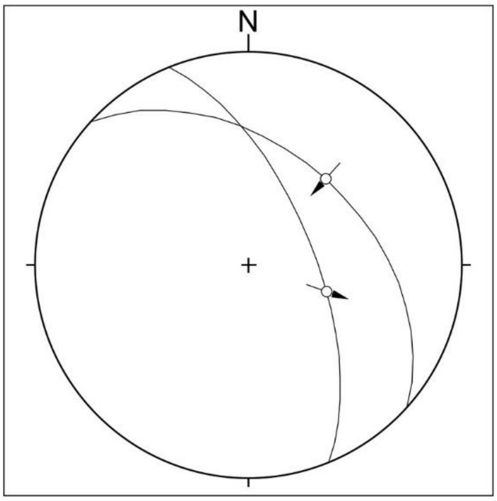

Fig. 9. Angelier diagram of fault-slip data (Equal-area stereographic projection, lower hemisphere) of reactivated faults from Interessante Höhle.

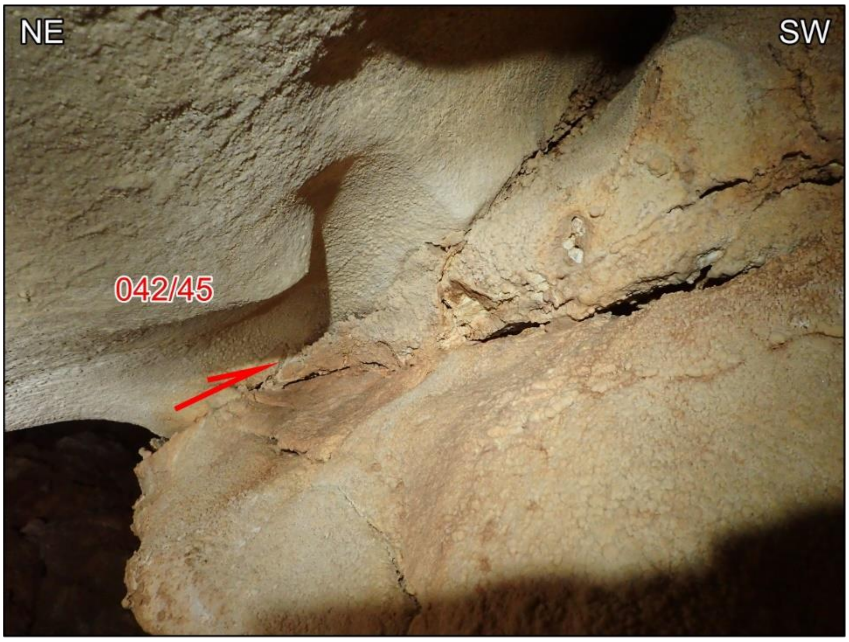

Fig. 10. Photograph of the reactivated fault in Interessante Höhle.

5. Tantalhöhle

No. of Austrian cave register:	1335/30	*Province*	Salzburg
Location	Hagengebirge NW, NE of Rauchegg	*Elevation of main entrance [m a.s.l.]*	1,710
UTM 33T Easting	355,478	*UTM 33T Northing*	5, 262,109
Length [m]	35,520	*Depth [m]*	435
Lithology	Dachsteinkalk, Hauptdolomit (both Upper Triassic)	*Key reference*	Klappacher & Knapczyk, 1979

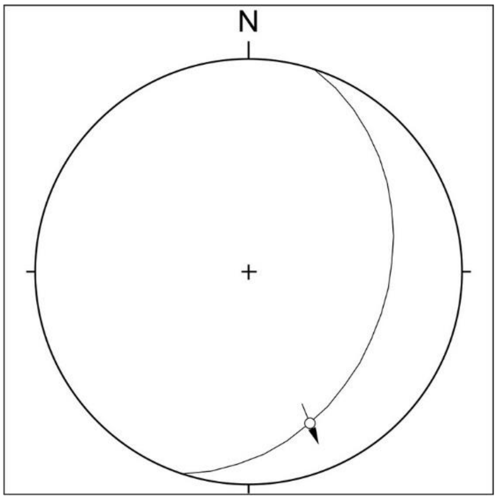

Fig. 11. Angelier diagram of fault-slip data (Equal-area stereographic projection, lower hemisphere) of reactivated faults from Tantalhöhle.

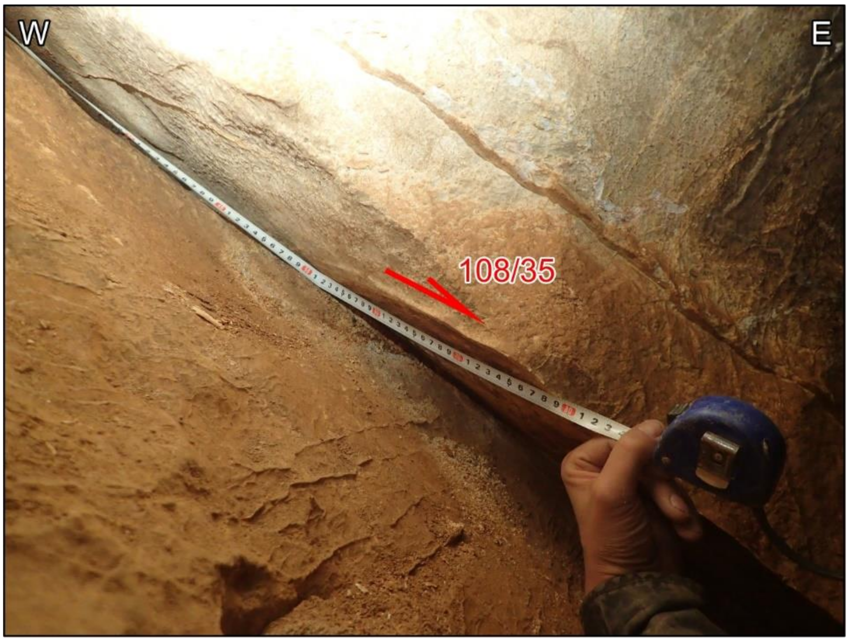

Fig. 12. Photograph of the reactivated fault in Tantalhöhle.

6. Bierloch (part of Berger-Plateneck-Höhlensystem)

No. of Austrian cave register:	1511/175	*Province*	Salzburg
Location	Tennengebirge NW, SE of Pass Lueg	*Elevation of main entrance [m a.s.l.]*	980
UTM 33T Easting	365,439	*UTM 33T Northing*	5,269,834
Length [m]	30,396	*Depth [m]*	1291
Lithology	Dachsteinkalk (Upper Triassic)	*Key reference*	Klappacher & Haseke-Knapczyk, 1985

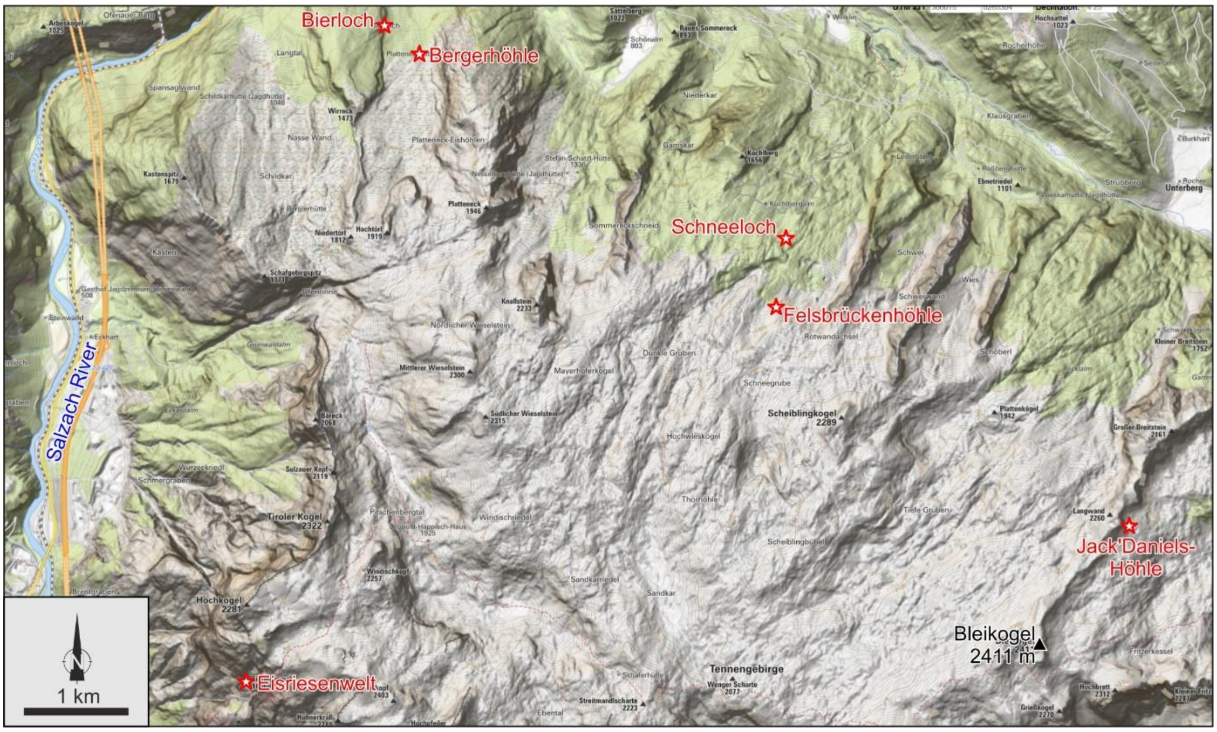

Fig. 13. Topographic map with the location of the cave in Tennengebirge.

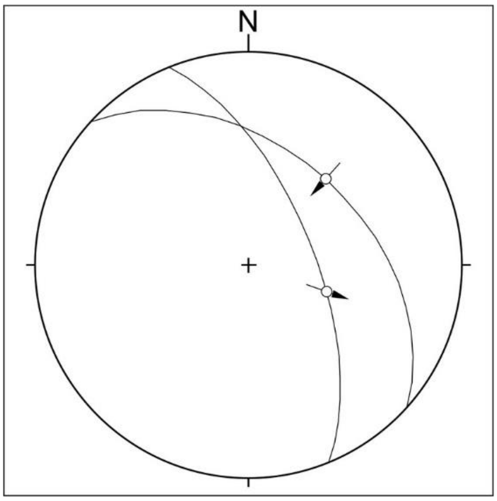

Fig. 14. Angelier diagram of fault-slip data (Equal-area stereographic projection, lower hemisphere) of reactivated faults from Bierloch.

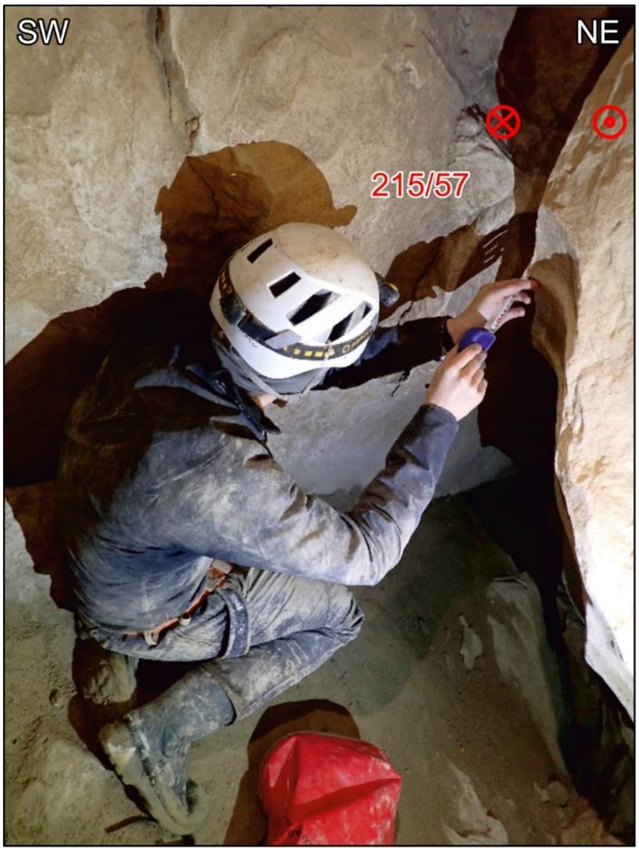

Fig. 15. Photograph of the reactiveted fault in Bierloch (photo M. Golicz).

7. Bergerhöhle (part of Berger-Plateneck-Höhlensystem)

No. of Austrian cave register:	1511/163	*Province*	Salzburg
Location	Tennengebirge NW, SE of Pass Lueg	*Elevation of main entrance [m a.s.l.]*	1031
UTM 33T Easting	365,786	*UTM 33T Northing*	5,269,676
Length [m]	30,396	*Depth [m]*	1291
Lithology	Dachsteinkalk (Upper Triassic)	*Key reference*	Klappacher & Haseke-Knapczyk, 1985

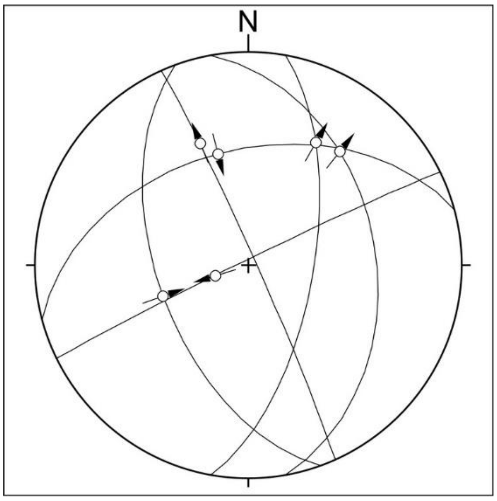

Fig. 16. Angelier diagram of fault-slip data (Equal-area stereographic projection, lower hemisphere) of reactivated faults from Bergerhöhle.

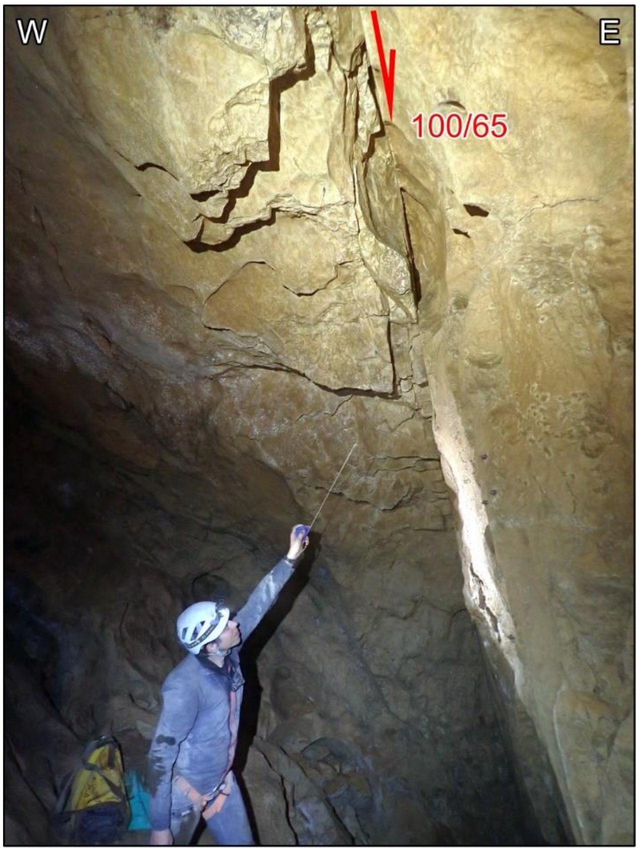

Fig. 17. Photograph of the reactivated fault in Bergerhöhle (photo M. Golicz).

8. Schneeloch (=Kuchelbergalmschacht)

No. of Austrian cave register:	1511/7	*Province*	Salzburg
Location	Tennengebirge N, NNW of Scheiblingkogel	*Elevation of main entrance [m a.s.l.]*	1615
UTM 33T Easting	369,406	*UTM 33T Northing*	5,267,778
Length [m]	10,319	*Depth [m]*	1101
Lithology	Dachsteinkalk (Upper Triassic)	*Key reference*	Klappacher & Haseke-Knapczyk, 1985

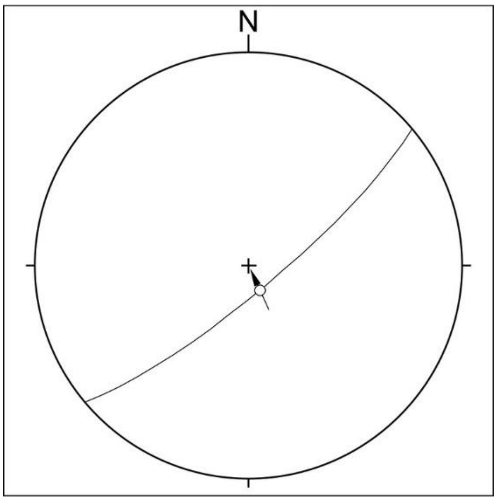

Fig. 18. Angelier diagram of fault-slip data (Equal-area stereographic projection, lower hemisphere) of reactivated faults from Schneeloch.

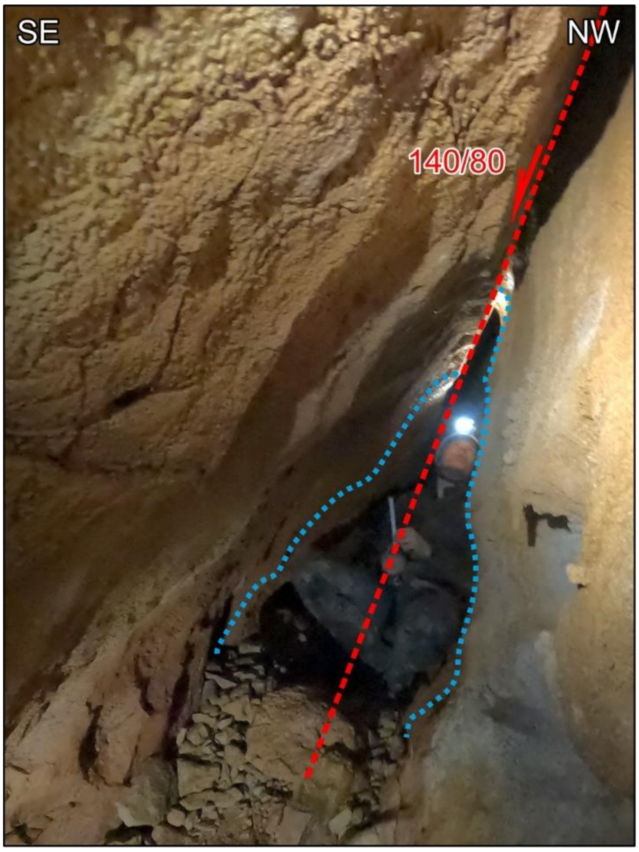

Fig. 19. Photograph of the reactivated fault in Schneeloch (photo M. Golicz).

9. Felsbrückenhöhle (part of Kuchlberg-Höhlensystem)

No. of Austrian cave register:	1511/930	*Province*	Salzburg
Location	Tennengebirge N, NW of Scheiblingkogel	*Elevation of main entrance [m a.s.l.]*	1866
UTM 33T Easting	369,277	*UTM 33T Northing*	5,267,023
Length [m]	26,118	*Depth [m]*	771
Lithology	Dachsteinkalk (Upper Triassic)	*Key reference*	Pointner, 2016

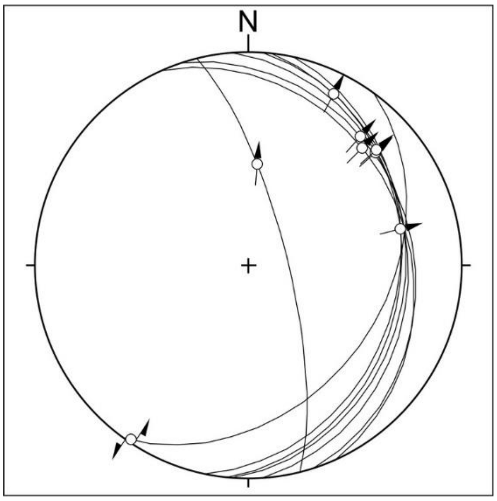

Fig. 20. Angelier diagram of fault-slip data (Equal-area stereographic projection, lower hemisphere) of reactivated faults from Felsbrückenhöhle.

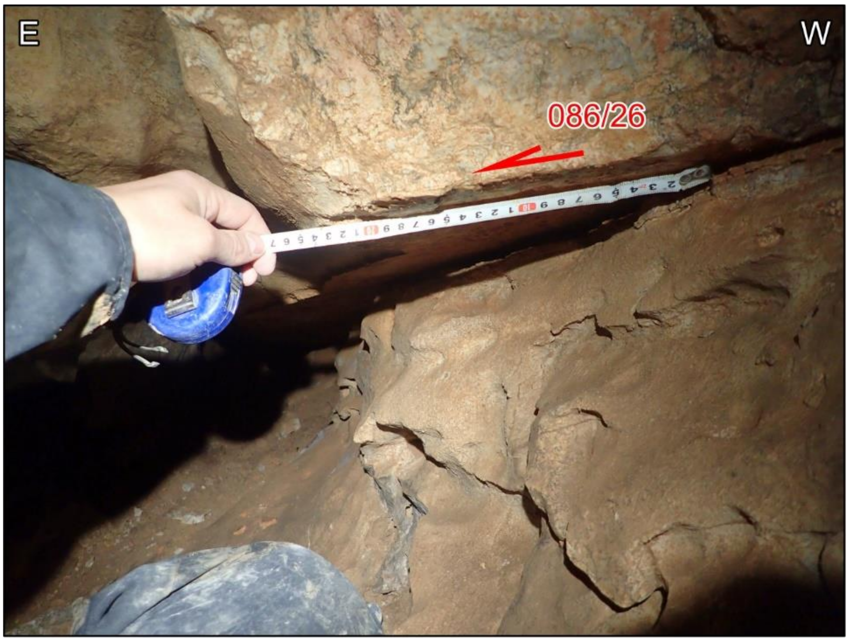

Fig. 21. Photograph of the reactivated fault in Felsbrückenhöhle.

10. Jack'Daniels-Höhle

No. of Austrian cave register:	1511/930	*Province*	Salzburg
Location	Tennengebirge E, NE of Bleikogel	*Elevation of main entrance [m a.s.l.]*	2111
UTM 33T Easting	372,759	*UTM 33T Northing*	5,264,800
Length [m]	10,135	*Depth [m]*	748
Lithology	Dachsteinkalk (Upper Triassic)	*Key reference*	Klappacher et al., 2006

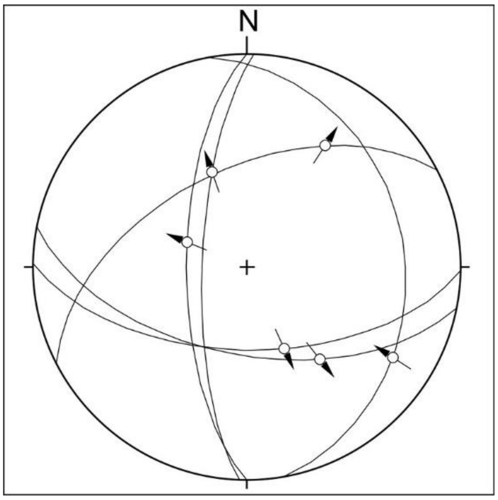

Fig. 22. Angelier diagram of fault-slip data (Equal-area stereographic projection, lower hemisphere) of reactivated faults from Jack'Daniels-Höhle.

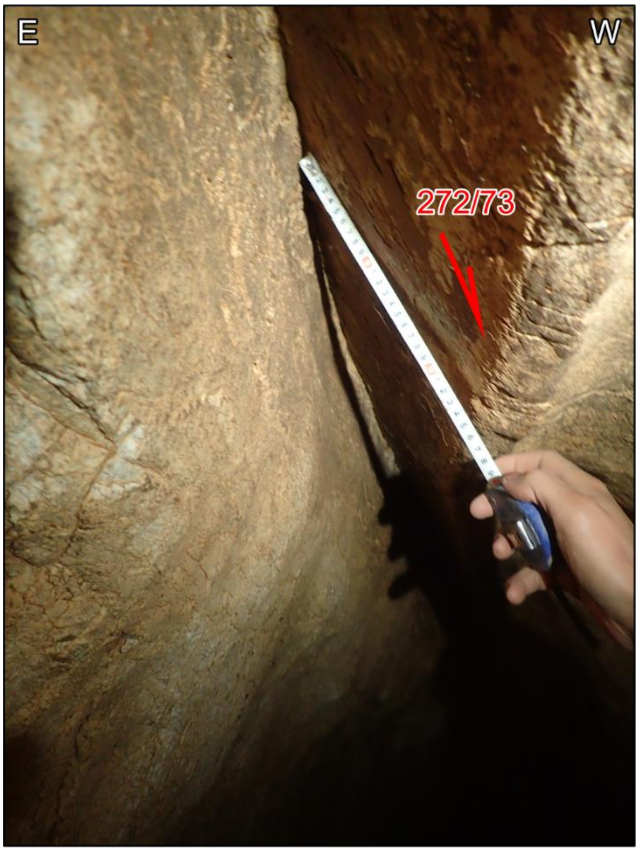

Fig. 23. Photograph of the reactivated fault in Jack'Daniels-Höhle.

11. Eisrisenwelt

No. of Austrian cave register:	1511/930	*Province*	Salzburg
Location	Tennengebirge W, NNE of Werfen	*Elevation of main entrance [m a.s.l.]*	1640
UTM 33T Easting	363,957	*UTM 33T Northing*	5,263,517
Length [m]	42,000	*Depth [m]*	442
Lithology	Dachsteinkalk and -dolomit (Upper Triassic)	*Key reference*	Plan et al., 2021

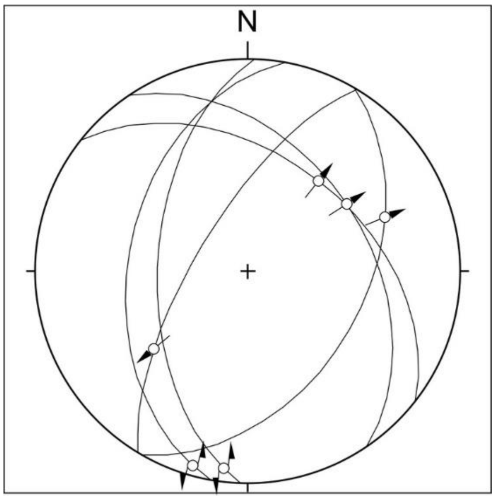

Fig. 24. Angelier diagram of fault-slip data (Equal-area stereographic projection, lower hemisphere) of reactivated faults from Eisrisenwelt.

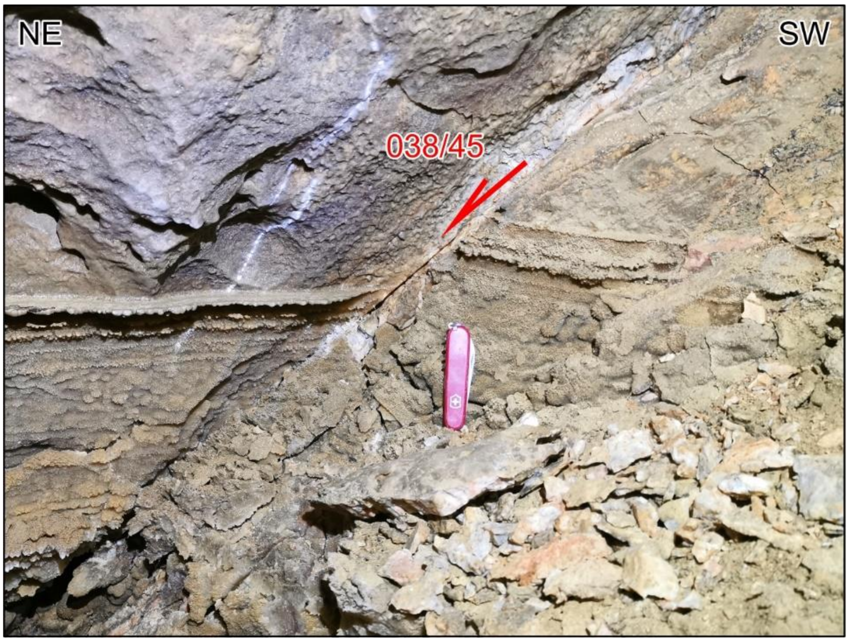

Fig. 25. Photograph of the reactivated fault (offset calcite shelfstone at former water table) in Eisrisenwelt.

12. *Hirlatzhöhle*

No. of Austrian cave register:	1546/7	*Province*	Upper Austria
Location	Dachstein N, SWof Hallstatt	*Elevation of main entrance [m a.s.l.]*	870
UTM 33T Easting	396,966	*UTM 33T Northing*	5,266,505
Length [m]	113,550	*Depth [m]*	1560
Lithology	Dachsteinkalk (U. Triassic), Wettersteindolomit (M. Trias.)	*Key reference*	Buchegger & Greger, 1999

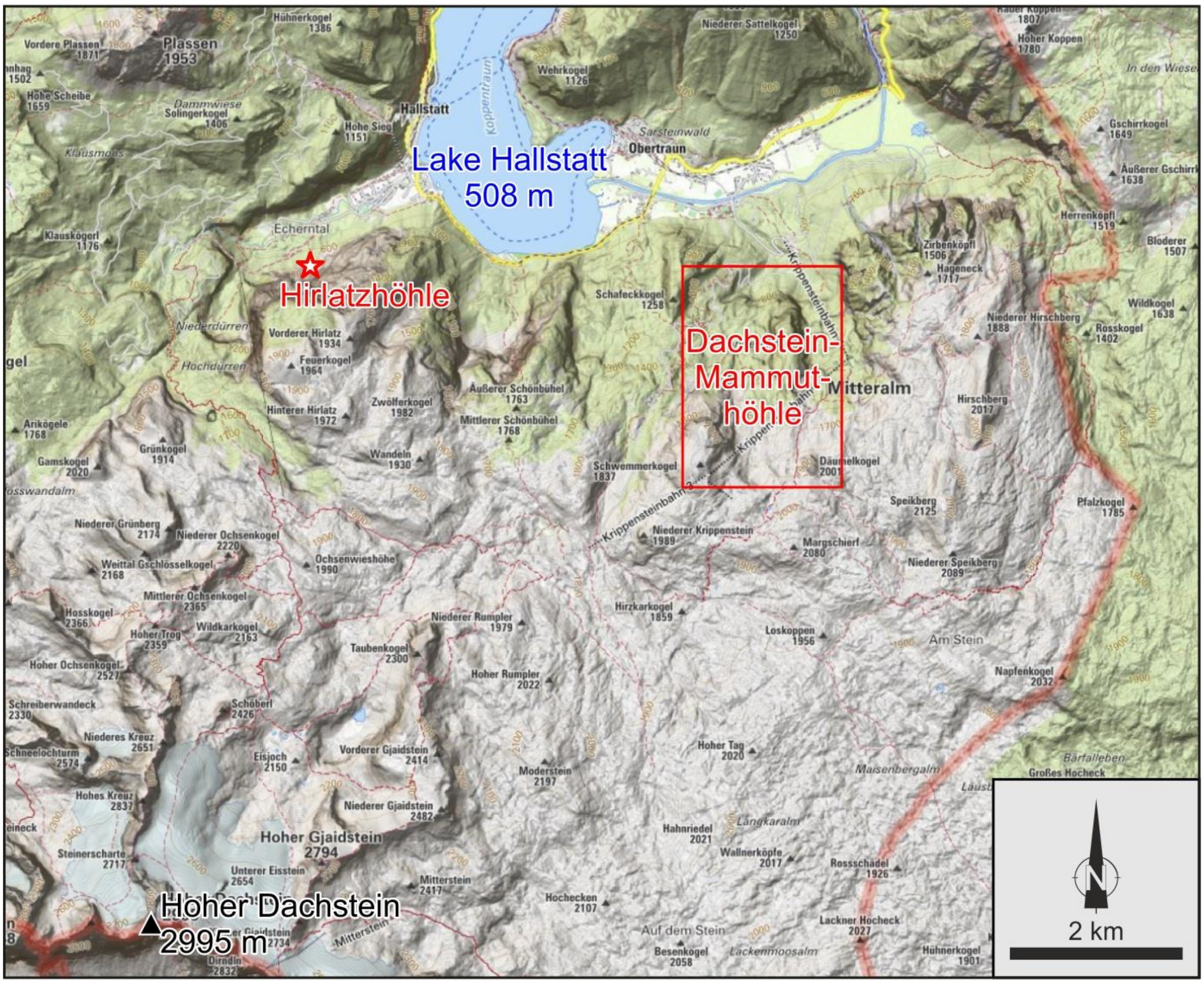

Fig. 26. Topographic map with the location of the caves in Dachstein.

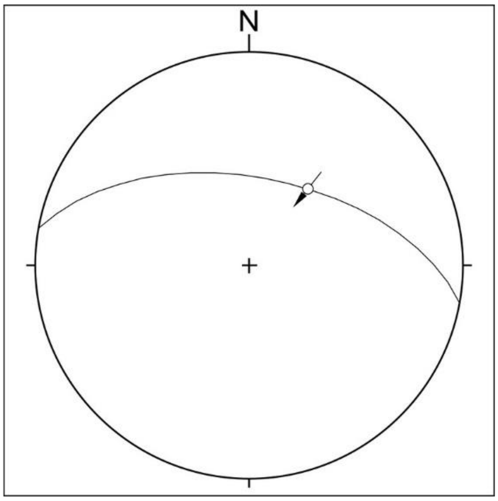

Fig. 27. Angelier diagram of fault-slip data (Equal-area stereographic projection, lower hemisphere) of reactivated faults from Hirlatzhöhle.

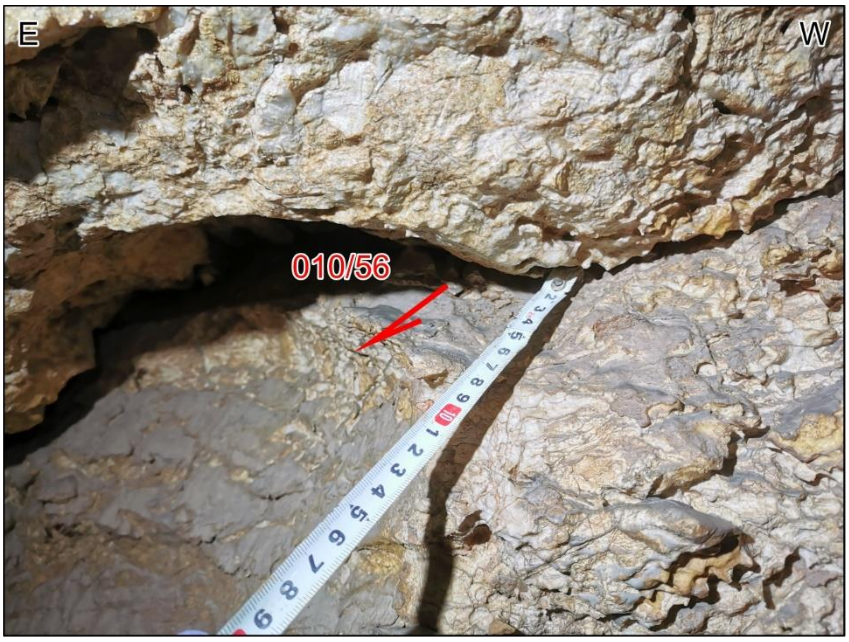

Fig. 3: Photograph of the reactivated fault in Hirlatzhöhle.

13. Dachstein-Mammuthöhle

No. of Austrian cave register:	1547/9	*Province*	Upper Austria
Location	N face of Dachstein,<br>S of village Obertraun	*Elevation of main entrance [m a.s.l.]*	1,321
UTM 33T Easting	402,829	*UTM 33T Northing*	5,265,422
Length [m]	67,725	*Depth [m]*	1,207
Lithology	Dachsteinkalk<br>(Upper Triassic)	*Key reference*	Behm et al., 2016

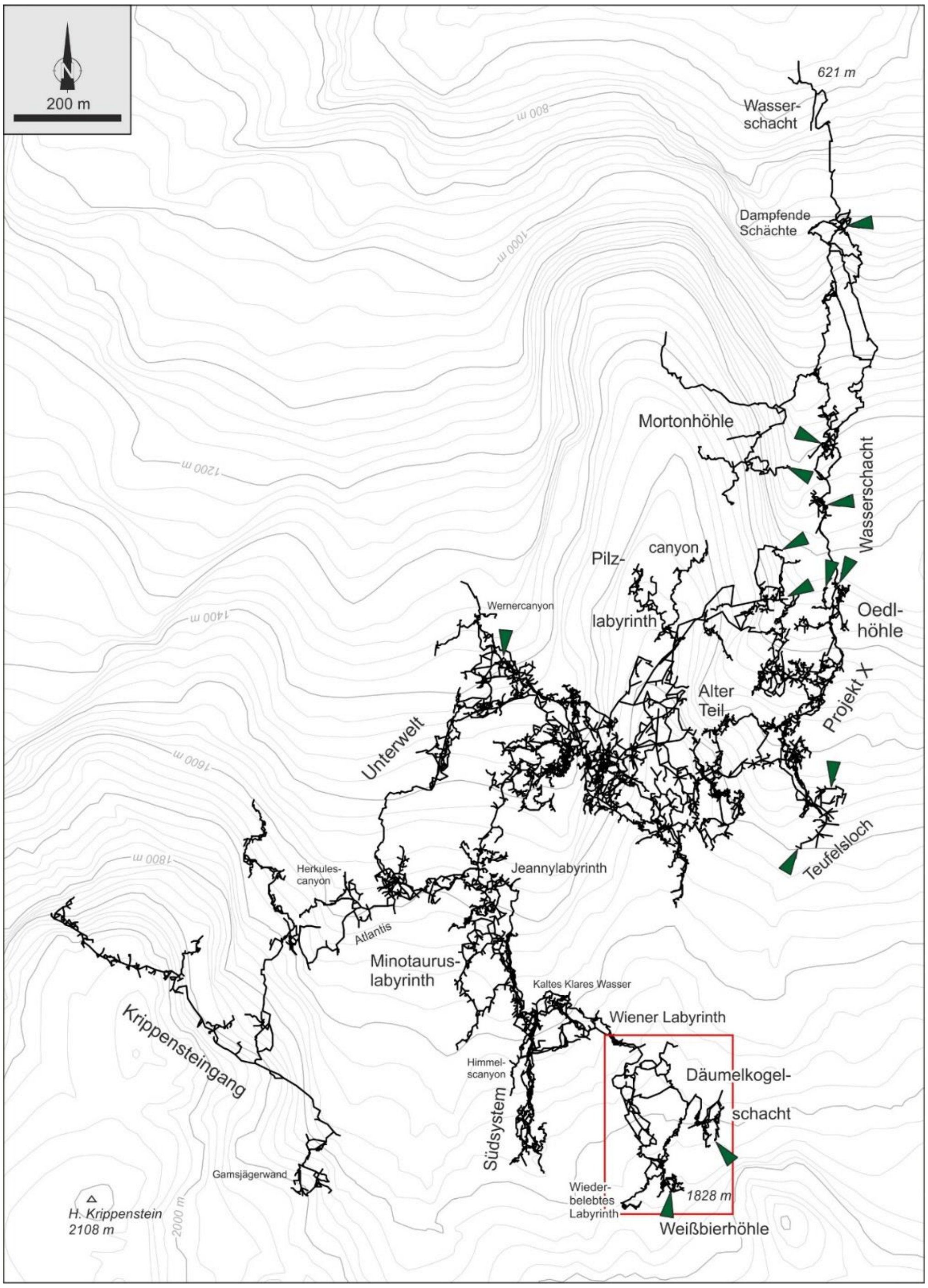

Fig. 29. Overview survey travers of Dachstein-Mammuthöhle (after Behm, M. and Plan, L. 2005).

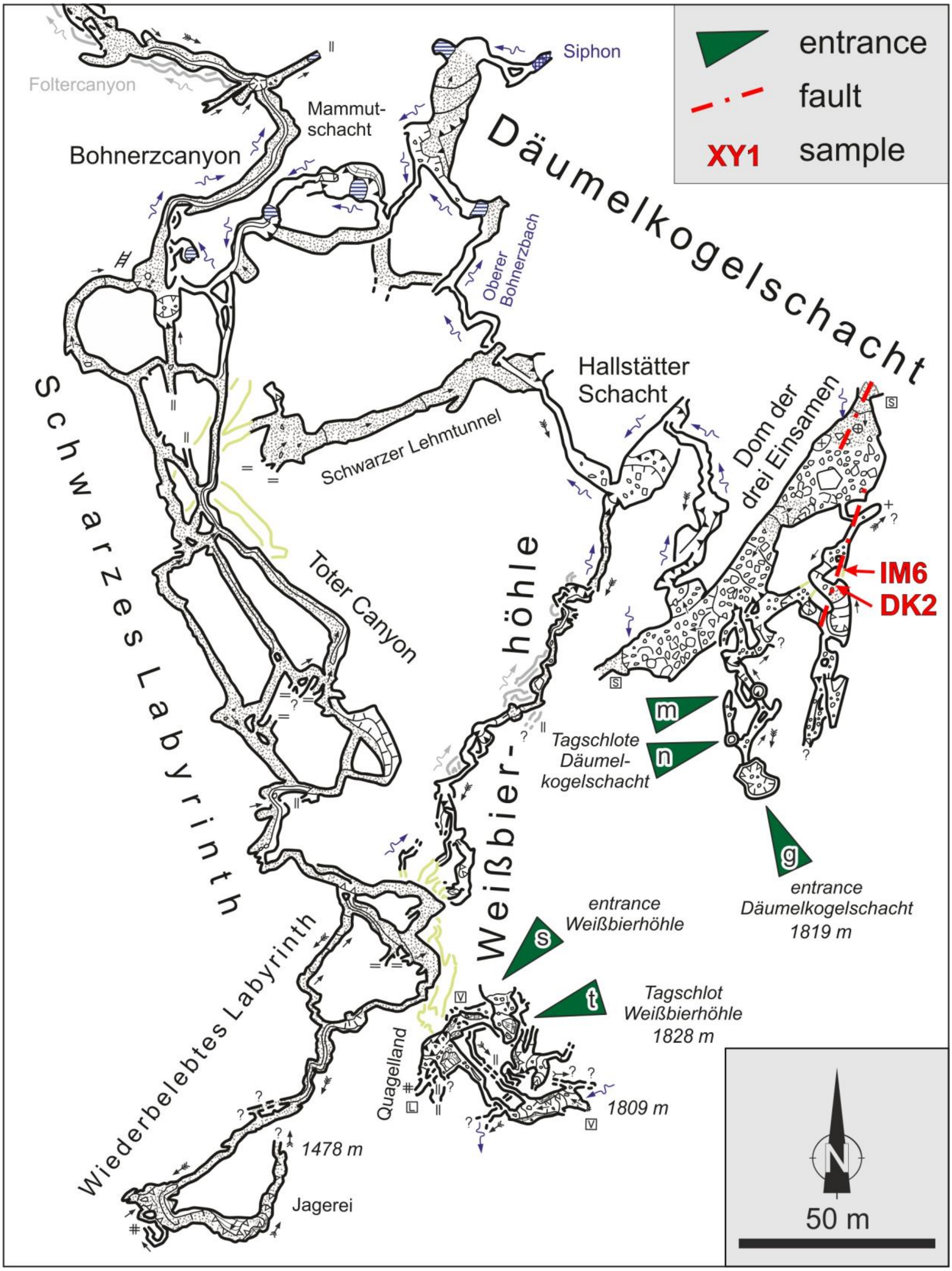

Fig. 30. Detaille of the map of Dachstein-Mammuthöhle of the part Däumelkogleschacht, where samples were taken. Map by: Xaver, A, Kula G. und Plan L. (2016).

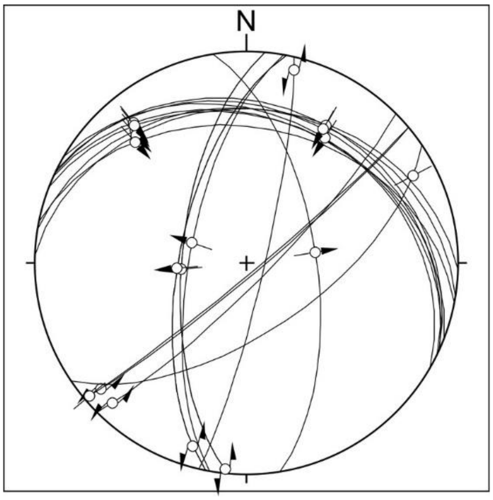

Fig.31. Angelier diagram of fault-slip data (Equal-area stereographic projection, lower hemisphere) of reactivated faults from Dachstein-Mammuthöhle.

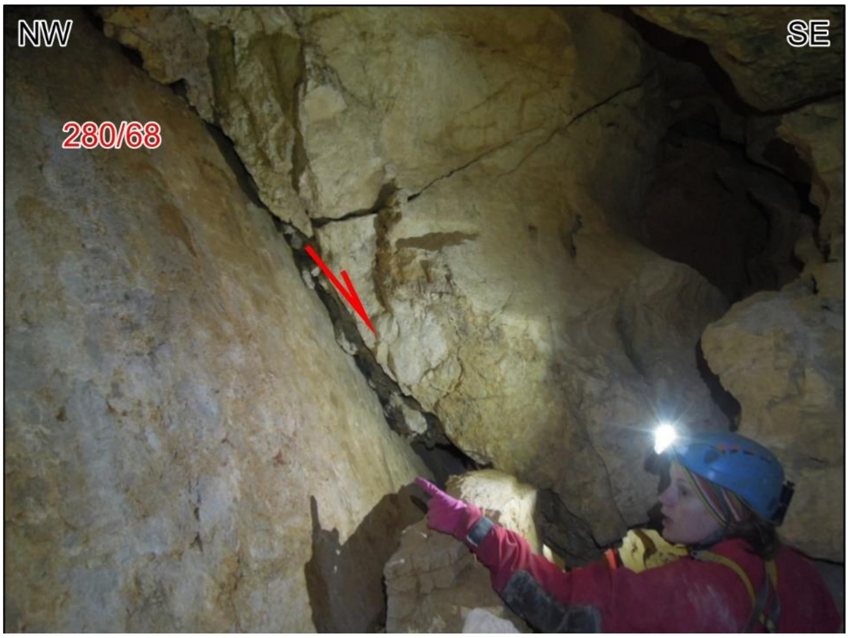

Fig. 32. Photograph of the reactivated fault in Dachstein-Mammuthöhle (in Däumelkogelschacht).

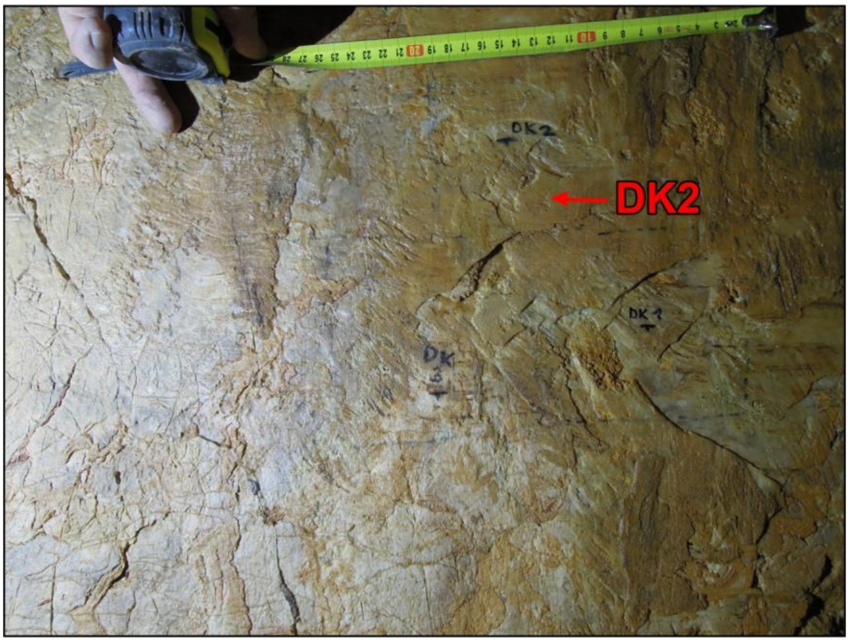

Fig.33. Location of sample DK2. Approximately 30 cm of the scale are is vible.

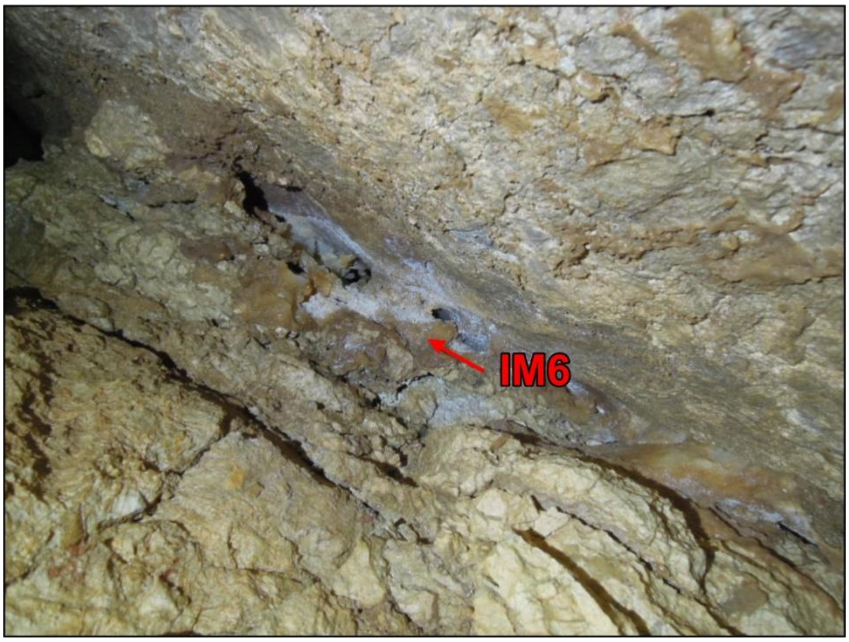

Fig. 34. Location of sample IM6. Width of the picture: 0.3 m.

14. Kugelmühle

No. of Austrian cave register:	1623/221	*Province*	Styria
Location	Totes Gebirge SW, NE Altaussee	*Elevation of entrance [m a.s.l.]*	804
UTM 33T Easting	409,686	*UTM 33T Northing*	5, 278,710
Length [m]	227	*Depth [m]*	109
Lithology	Dachsteinkalk (Upper Triassic)	*Key reference*	Seebacher, 2015a

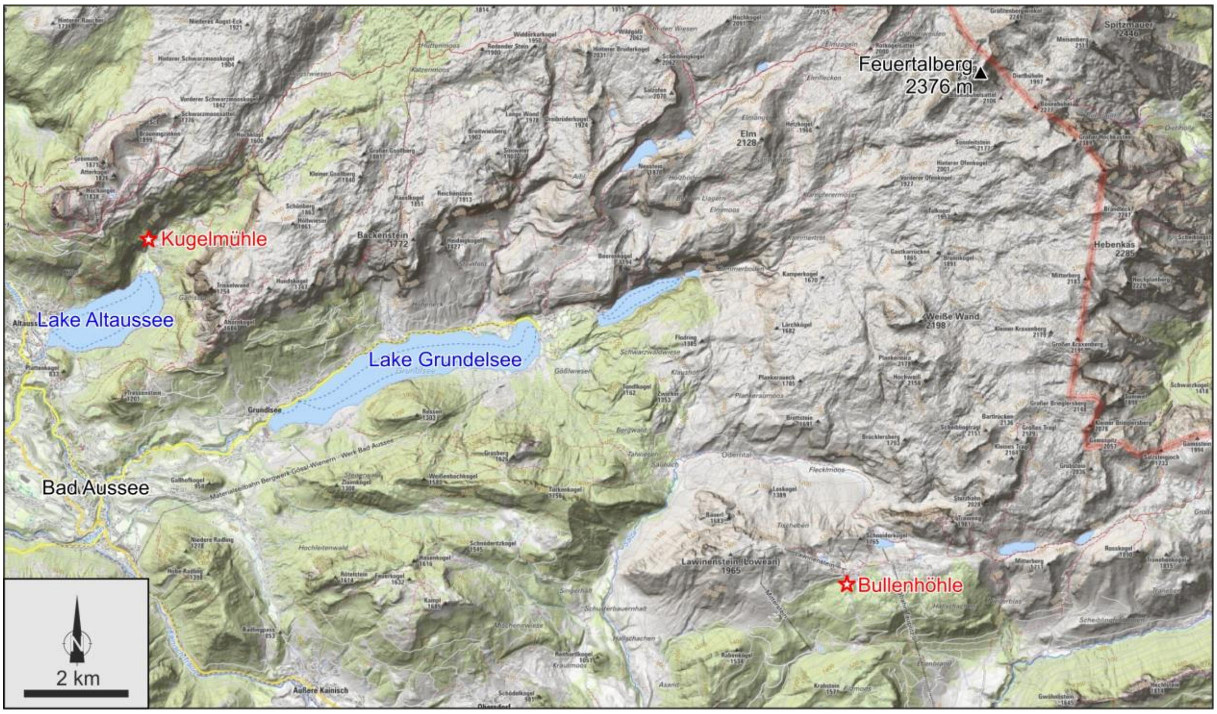

Fig. 35. Topographic map with the location of the caves at the south side of Totes Gebirge.

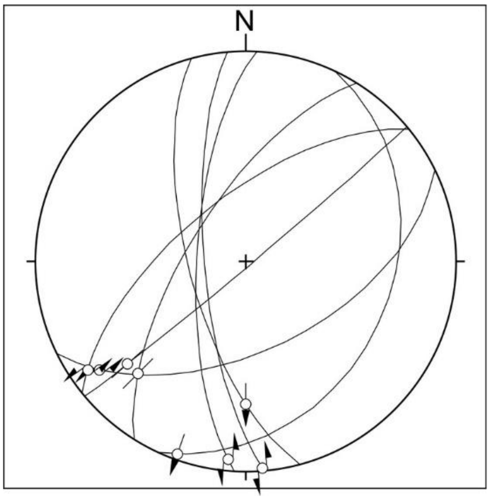

Fig. 36. Angelier diagram of fault-slip data (Equal-area stereographic projection, lower hemisphere) of reactivated faults from Kugelmühle.

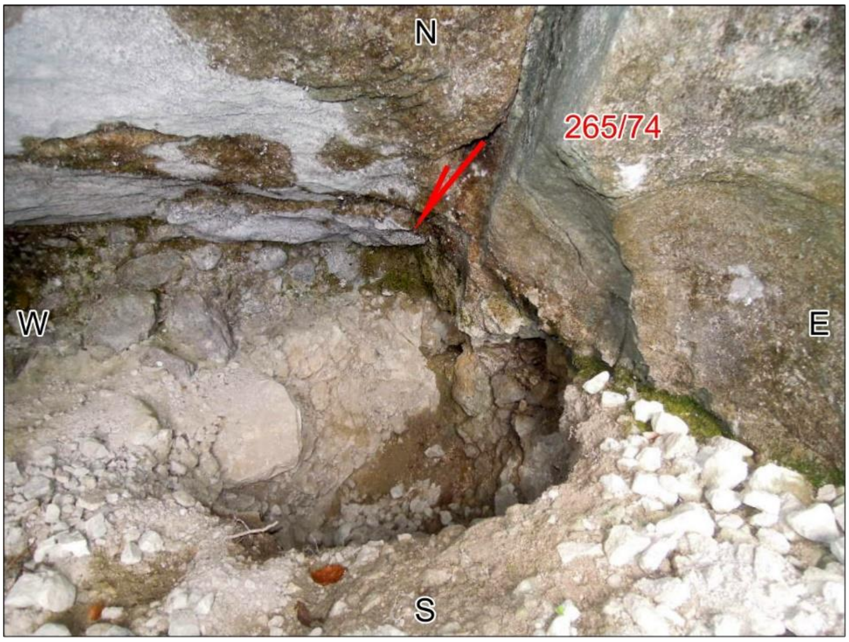

Fig. 37. Photograph of the reactivated fault in Kugelmühle; downward view.

15. Bullenhöhle

No. of Austrian cave register:	1622/57	*Province*	Styria
Location	Totes Gebirge SE, NE Bad Mitterndorf	Elevation of entrance [m a.s.l.]	1570
UTM 33T Easting	423,585	*UTM 33T Northing*	5,271,403
Length [m]	1484	*Depth [m]*	256
Lithology	Dachsteinkalk (Upper Triassic)	*Key reference*	Seebacher, 2015b

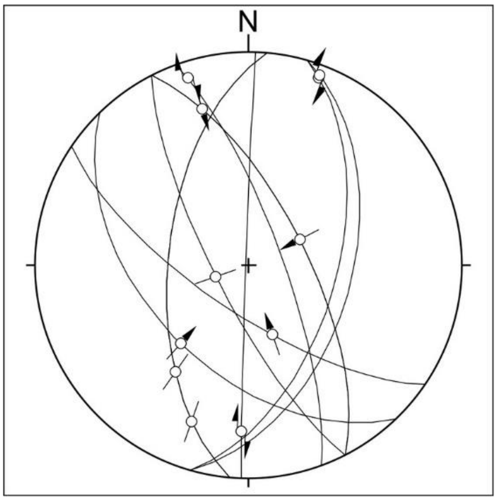

Fig. 38. Angelier diagram of fault-slip data (Equal-area stereographic projection, lower hemisphere) of reactivated faults from Bullenhöhle.

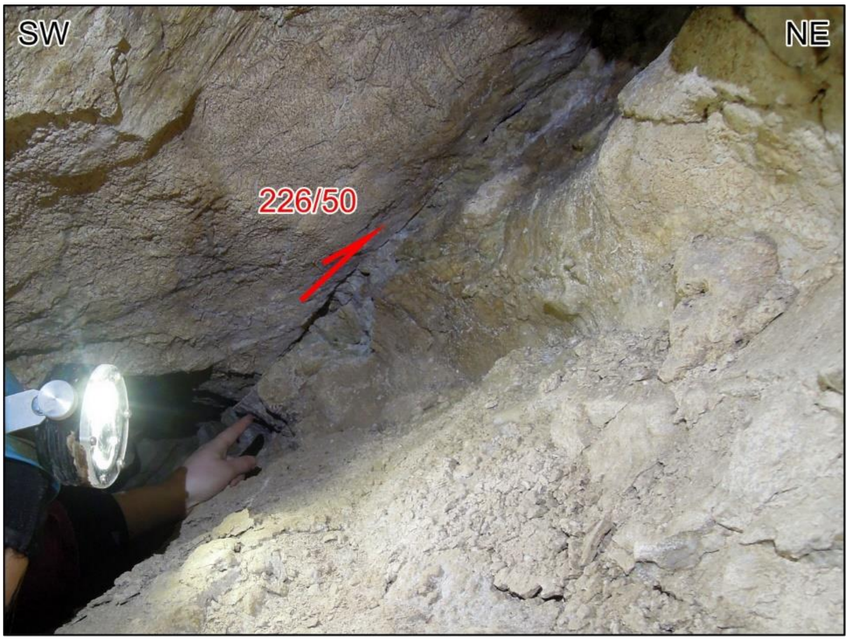

Fig. 39. Photograph of the reactivated fault in Bullenhöhle; oblique upward view.

16. POL-Nord-Ponor

No. of Austrian cave register:	1744/534	*Province*	Styria
Location	Hochschwab N, NE Ebenstein	*Elevation of entrance [m a.s.l.]*	1710
UTM 33T Easting	503,190	*UTM 33T Northing*	5273,150
Length [m]	493	*Depth [m]*	190
Lithology	Wettersteinkalk (Middle Triassic)	*Key reference*	Plan et al., 2017

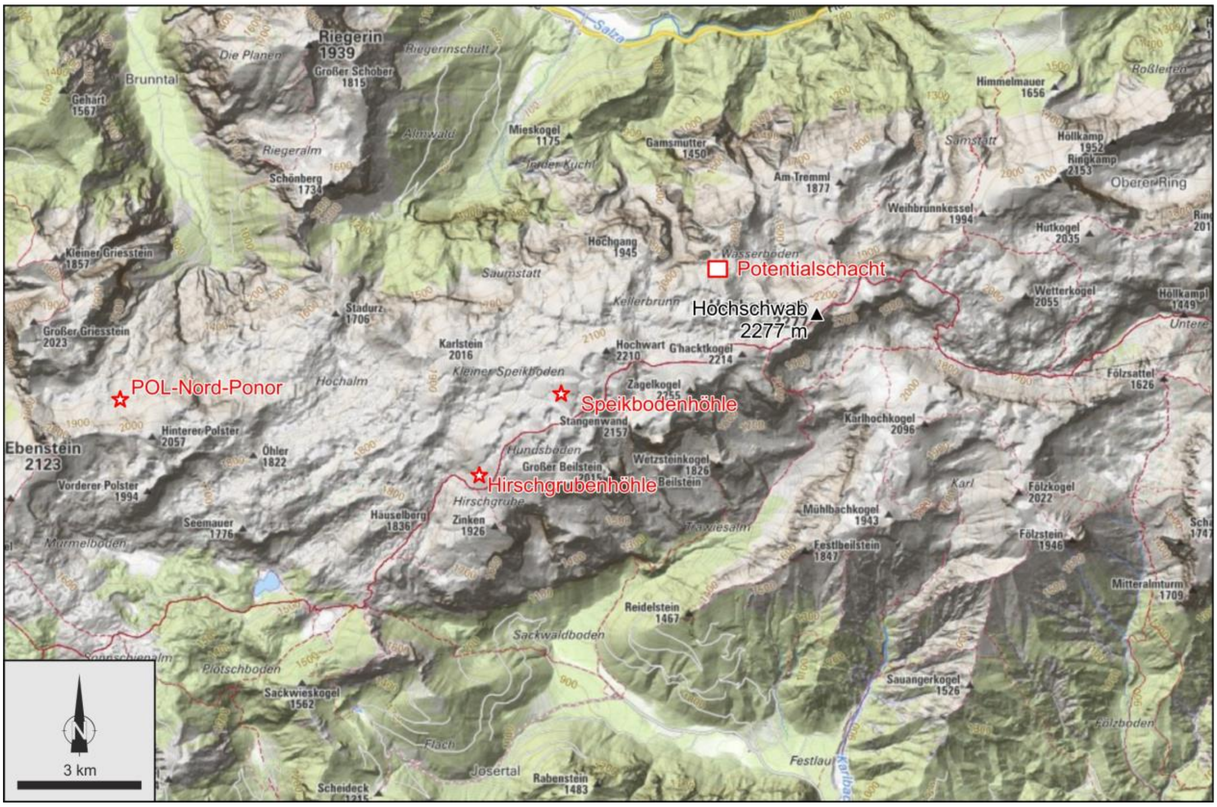

Fig. 40. Topographic map with the location of the caves in Hochschwab.

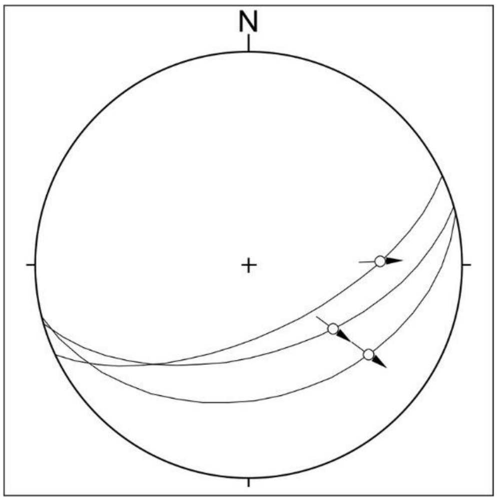

Fig. 41. Angelier diagram of fault-slip data (Equal-area stereographic projection, lower hemisphere) of reactivated faults from POL-Nord-Ponor.

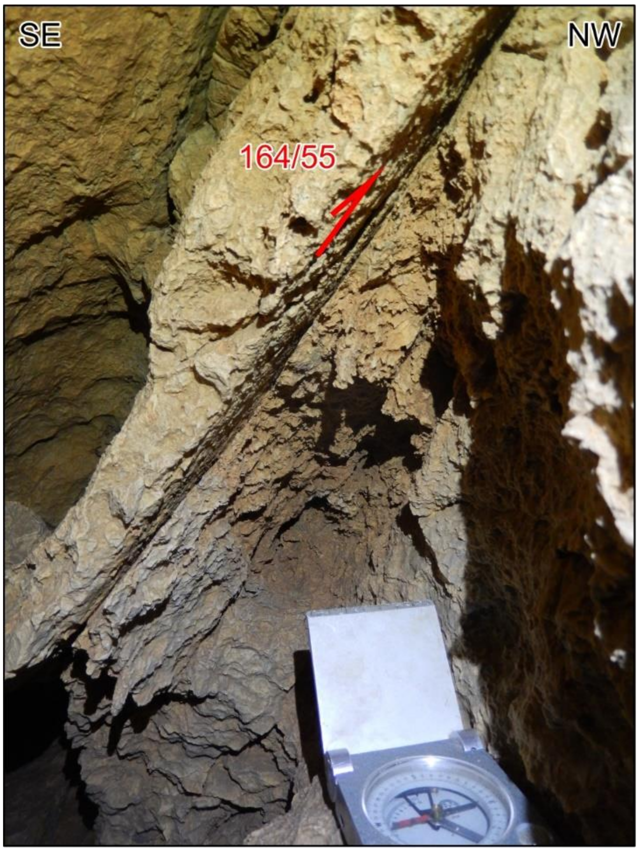

Fig. 42. Photograph of oblique upward fault in POL-Nord-Ponor.

17. Speikbodenhöhle

No. of Austrian cave register:	1744/650	*Province*	Styria
Location	Hochschwab centre, SW Hochwart	*Elevation of entrance [m a.s.l.]*	2,100
UTM 33T Easting	507,874	*UTM 33T Northing*	5,273,000
Length [m]	1,936	*Depth [m]*	277
Lithology	Dachsteinkalk/Wettersteindolomite (Middle and Upper Triassic)	*Key reference*	Plan et al., 2021

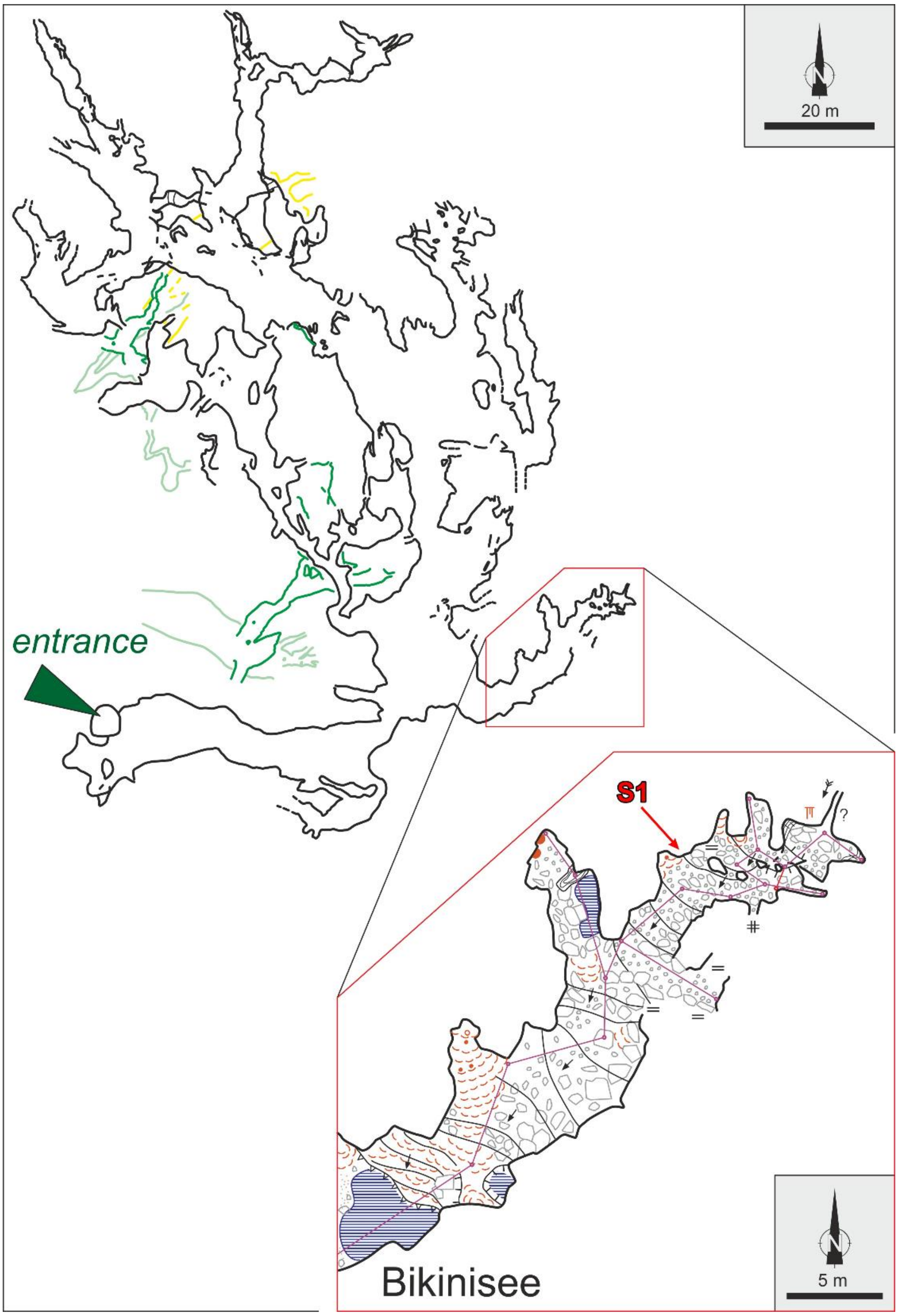

Fig. 43. Overview map of Speikbodenhöhle and insert with the detailed location of sample S1. Map by: Plan L. (2021).

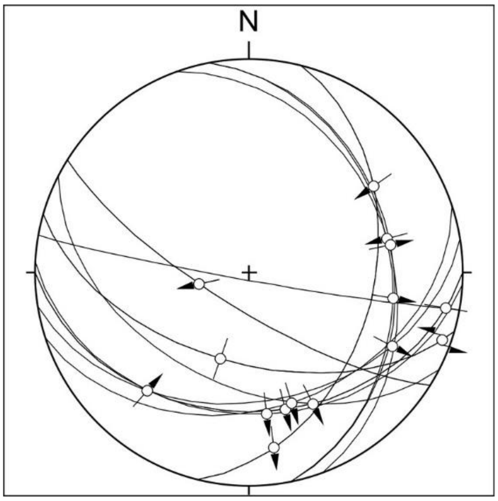

Fig. 44. Angelier diagram of fault-slip data (Equal-area stereographic projection, lower hemisphere) of reactivated faults from Speikbodenhöhle.

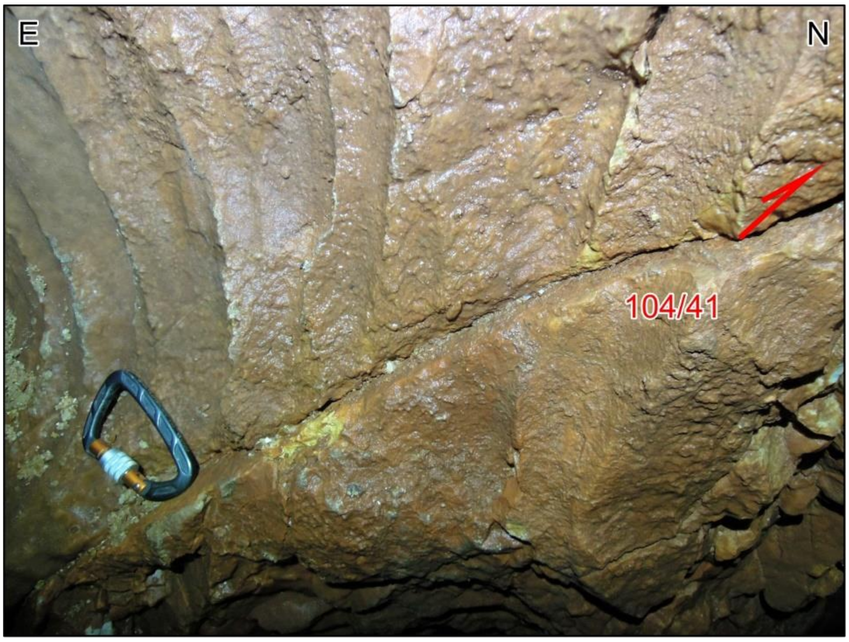

Fig. 45. Photograph of oblique upward fault in Speikbodenhöhle.

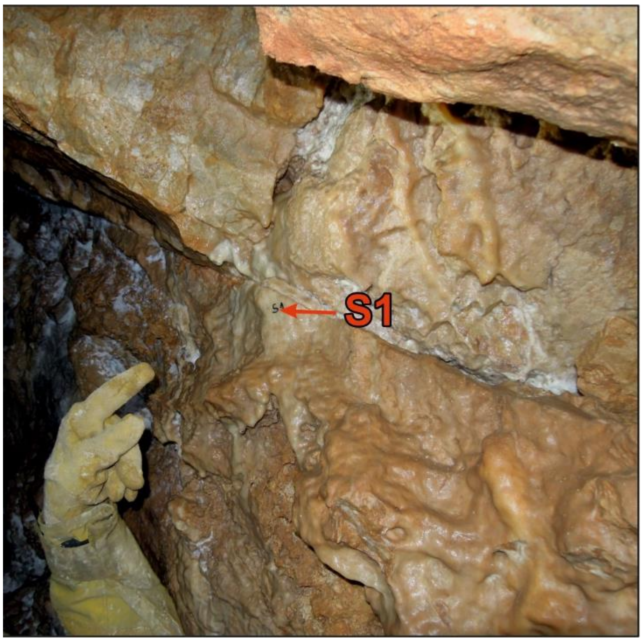

Fig. 46. Photo of the sample location in Speikbodenhöhle.

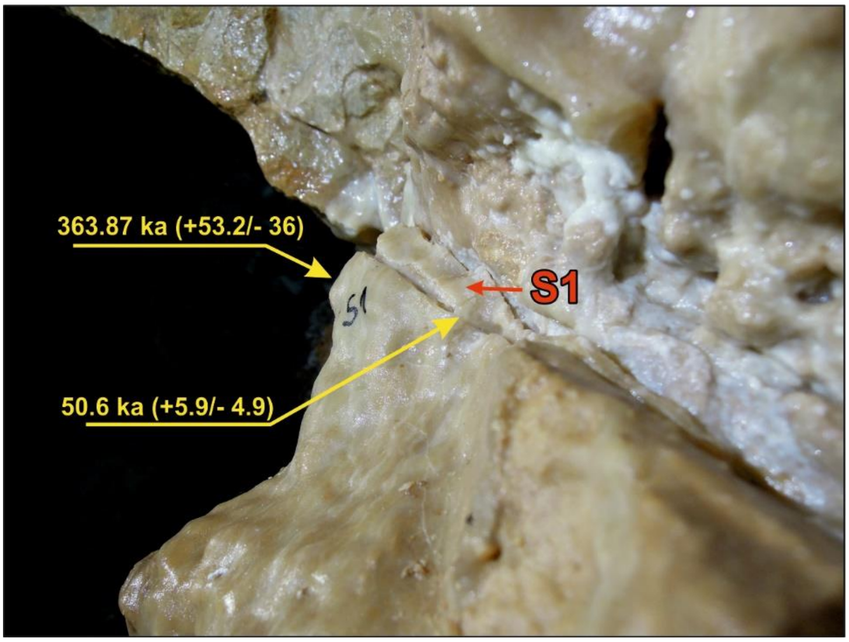

Fig. 47. Detail of sample S1 (including dating result). Width of the picture is 10 cm.

18. Potentialschacht

No. of Austrian cave register:	1744/475	*Province*	Styria
Location	Hochschwab centre, E Hochgang	*Elevation of entrance [m a.s.l.]*	2,070
UTM 33T Easting	509,511	*UTM 33T Northing*	5,274,334
Length [m]	2,329	*Depth [m]*	107
Lithology	Wettersteinkalk/-dolomite (Mid. Triassic)	*Key reference*	Plan and Baroň, 2021

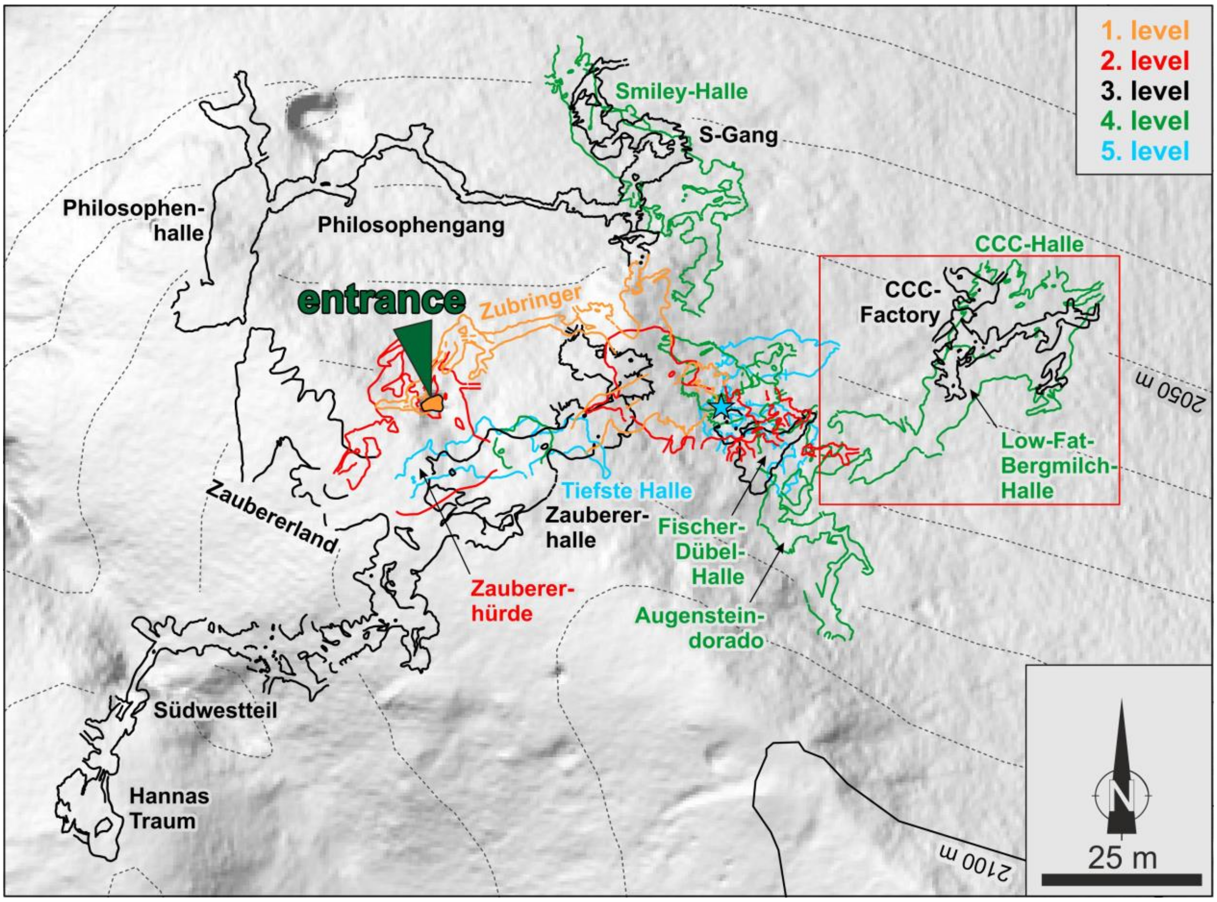

Fig. 48. Overview map of Potentialschacht. Map by: Plan L. (2021).

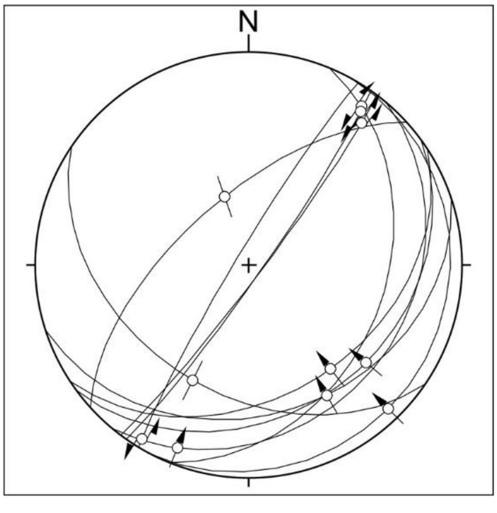

Fig.49. Angelier diagram of fault-slip data (Equal-area stereographic projection, lower hemisphere) of reactivated faults from Potentialschacht.

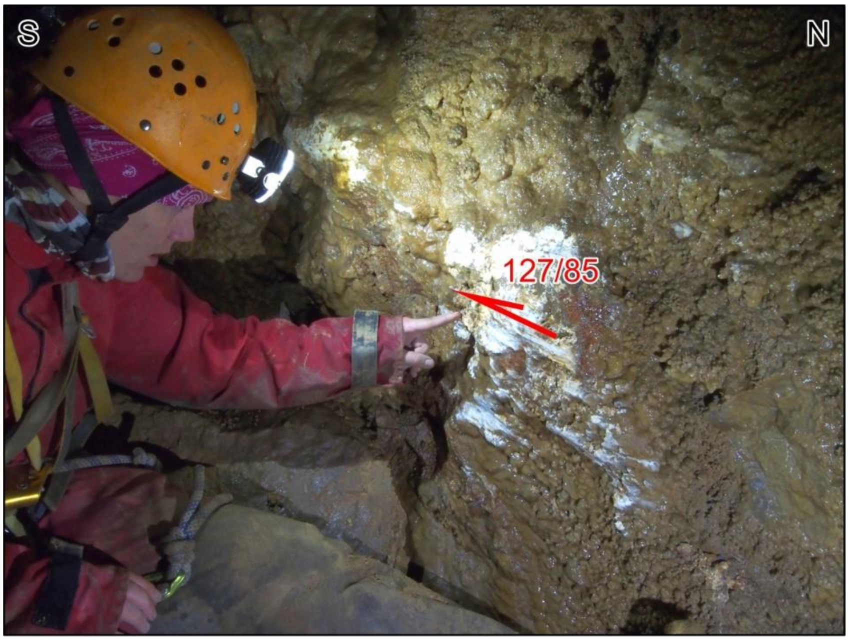

Fig. 50. Photograph of oblique upward fault in Potentialschacht.

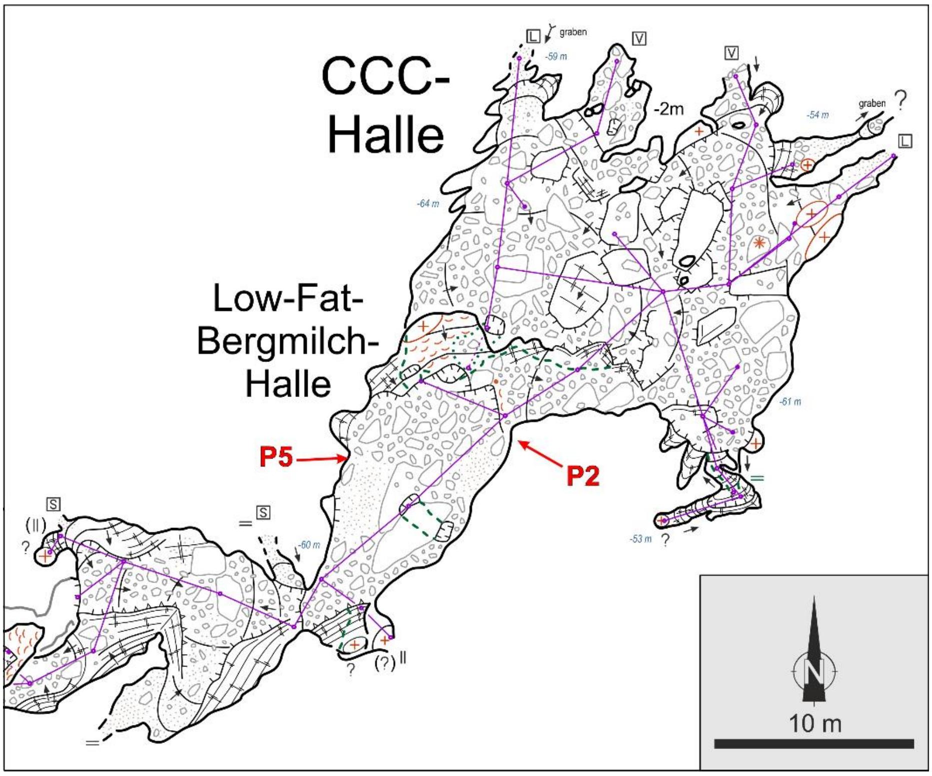

Fig. 51. Detailed map of Potentialschacht with sample locations. Map by: Plan L. (2021).

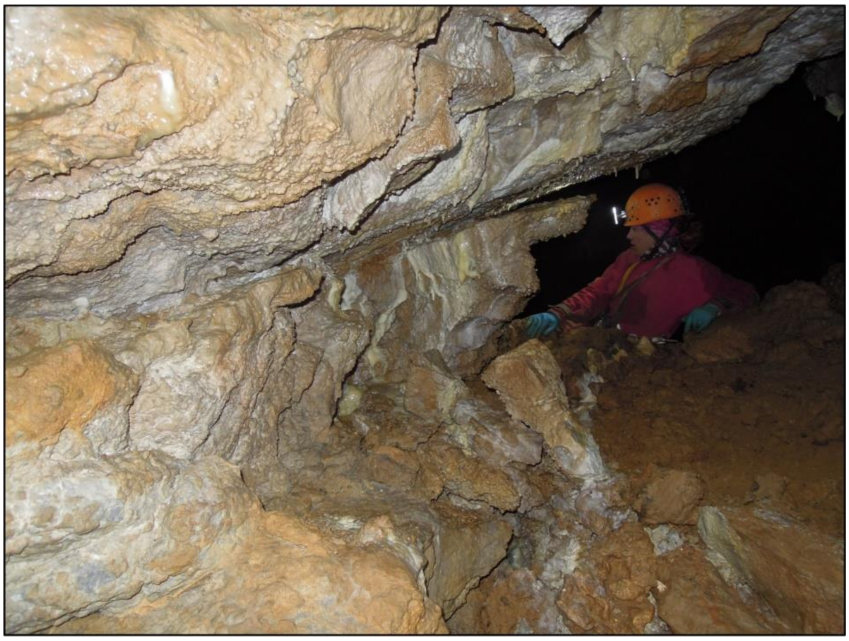

Fig. 52. Photo of the sample location in Potentialschacht. Overview of the fault.

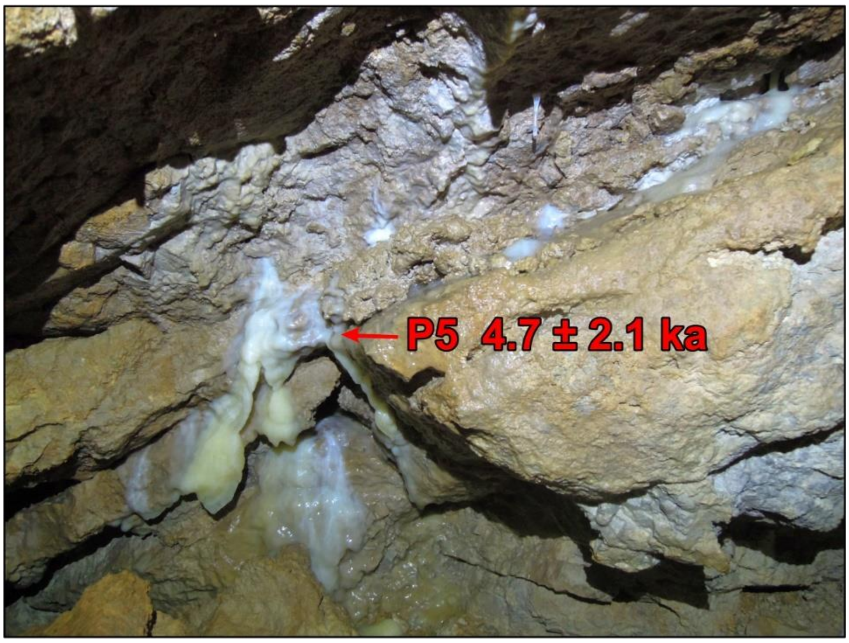

Fig. 53. Location of sample P5 (including dating result). Width of the picture 0.5 m.

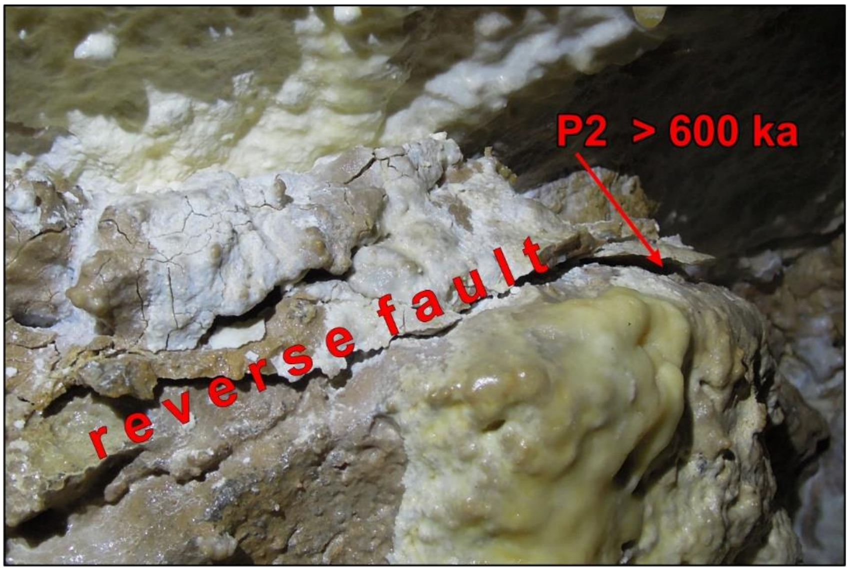

Fig. 54. Location of sample P2 (including dating result in years). Width of the picture c. 15 cm.

19 Hirschgrubenhöhle

No. of Austrian cave register:	1744/450	*Province*	Styria
Location	Hochschwab centre, NW of Zinken	*Elevation of entrance [m a.s.l.]*	1,896
UTM 33T Easting	506,987	*UTM 33T Northing*	5,272,020
Length [m]	5,596	*Depth [m]*	201
Lithology	Dachsteinkalk (U. Triasic), Wettersteindolomite (Mid. Tr.)	*Key reference*	Plan et al., 2019

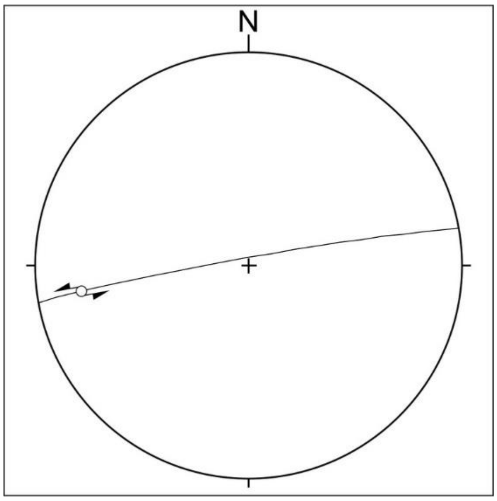

Fig. 55. Angelier diagram of fault-slip data (Equal-area stereographic projection, lower hemisphere) of reactivated faults from Hirschgrubenhöhle.

20 Große Offenbergerhöhle

No. of Austrian cave register:	1733/1	*Province*	Styria
Location	N St. Lorenzen im Mürztal	*Elevation of entrance [m a.s.l.]*	766
UTM 33T Easting	527,512	*UTM 33T Northing*	5,261,473
Length [m]	366	*Depth [m]*	21
Lithology	Limestone marble (Triassic)	*Key reference*	Oberender et al., 2017

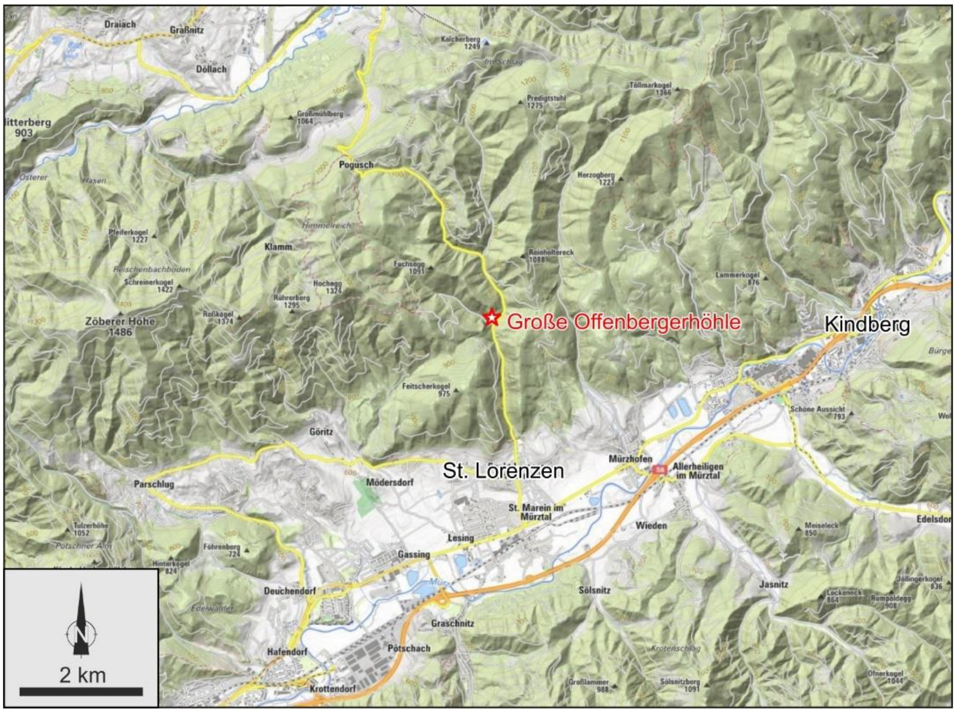

Fig. 56. Topographic map with the location of Große Offenbergerhöhle.

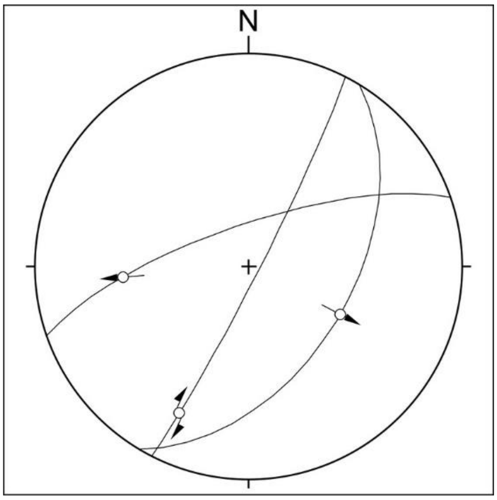

Fig. 57. Angelier diagram of fault-slip data (Equal-area stereographic projection, lower hemisphere) of reactivated faults from Große Offenbergerhöhle.

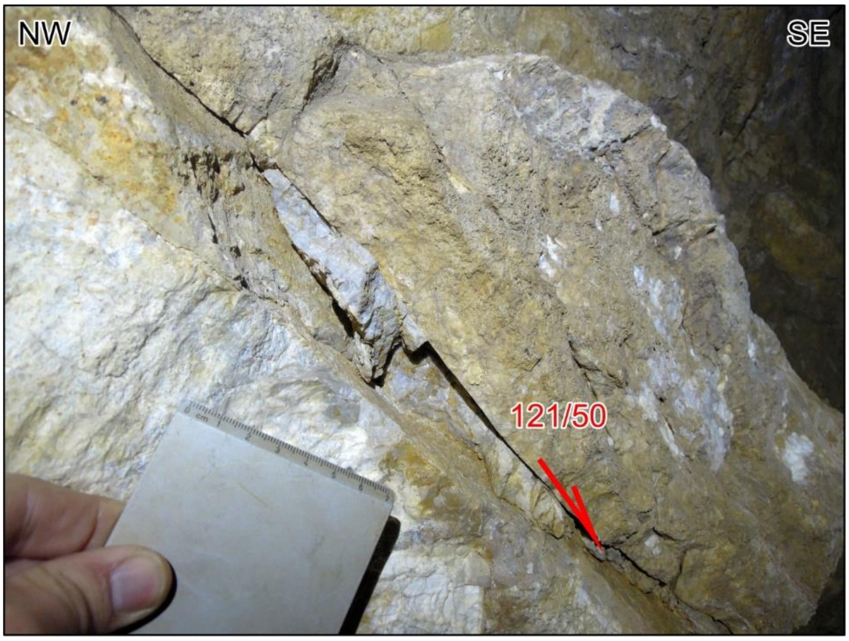

Fig. 58. Photograph of the reactivated fault in Große Offenbergerhöhle.

21 Zederhaushöhle

No. of Austrian cave register:	2861/17	*Province*	Styria
Location	NW Spital am Semmering	*Elevation of entrance [m a.s.l.]*	915
UTM 33T Easting	555,635	*UTM 33T Northing*	5,273,923
Length [m]	57	*Depth [m]*	19
Lithology	Limestone marble (Triassic)	*Key reference*	Fink et al., 1979

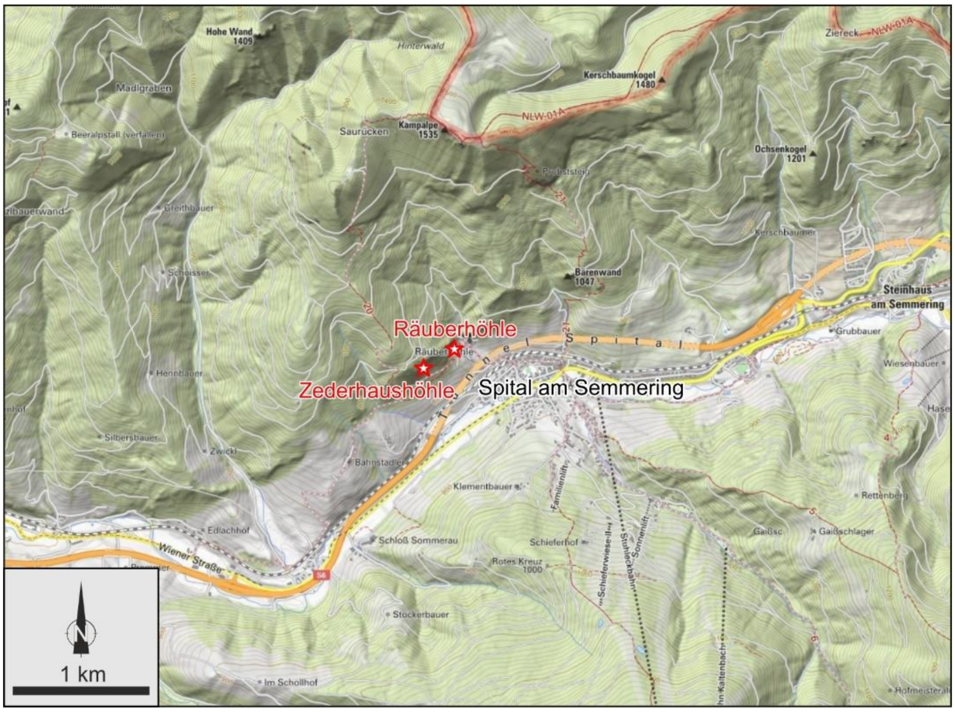

Fig. 59. Topographic map with the location of caves near Spital am Semmering.

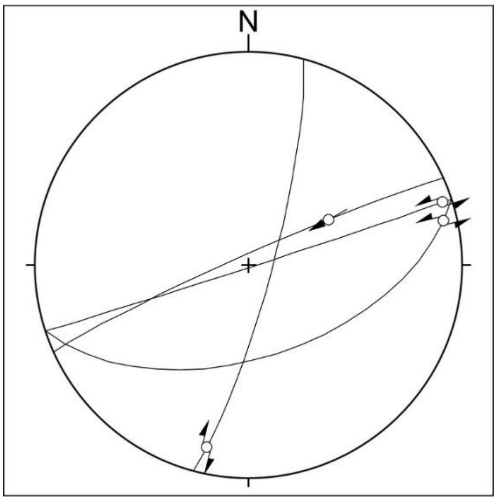

Fig. 60. Angelier diagram of fault-slip data (Equal-area stereographic projection, lower hemisphere) of reactivated faults from Zederhaushöhle.

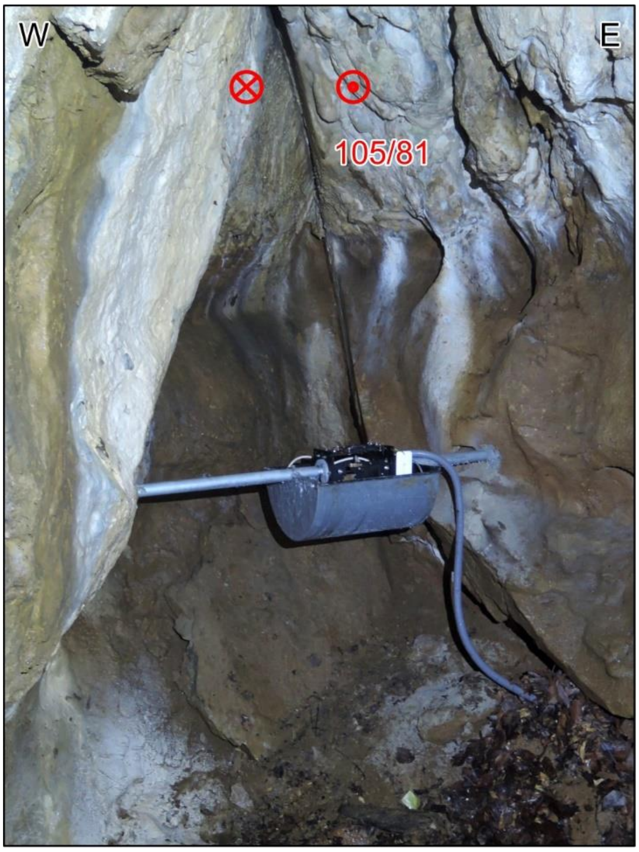

Fig. 61. Photograph of the reactivated fault in Zederhaushöhle.

22 Räuberhöhle

No. of Austrian cave register:	2861/12	*Province*	Styria
Location	NW Spital am Semmering	*Elevation of entrance [m a.s.l.]*	890
UTM 33T Easting	555,824	*UTM 33T Northing*	5,274,074
Length [m]	120	*Depth [m]*	25
Lithology	Limestone marble (Triassic)	*Key reference*	Fink et al., 1979

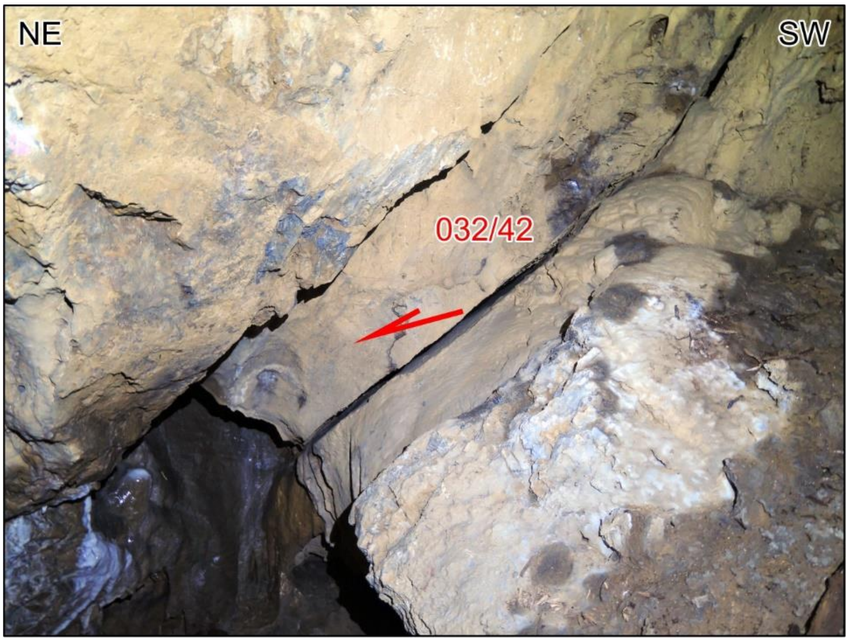

Fig. 62. Photograph of the reactivated fault in Räuberhöhle; oblique upward view.

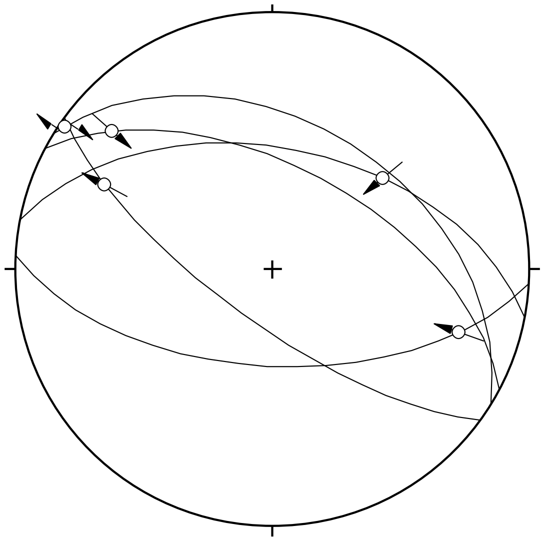

Fig. 63. Angelier diagram of fault-slip data (Equal-area stereographic projection, lower hemisphere) of reactivated faults from Räuberhöhle.

23 Hermannshöhle

No. of Austrian cave register:	2871/7	*Province*	Lower Austria
Location	NW Kirchberg am Wechsel	*Elevation of entrance [m a.s.l.]*	629
UTM 33T Easting	573,799	*UTM 33T Northing*	5,274,222
Length [m]	4430	*Depth [m]*	73
Lithology	Limestone marble (Triassic)	*Key reference*	Plan et al., 2015

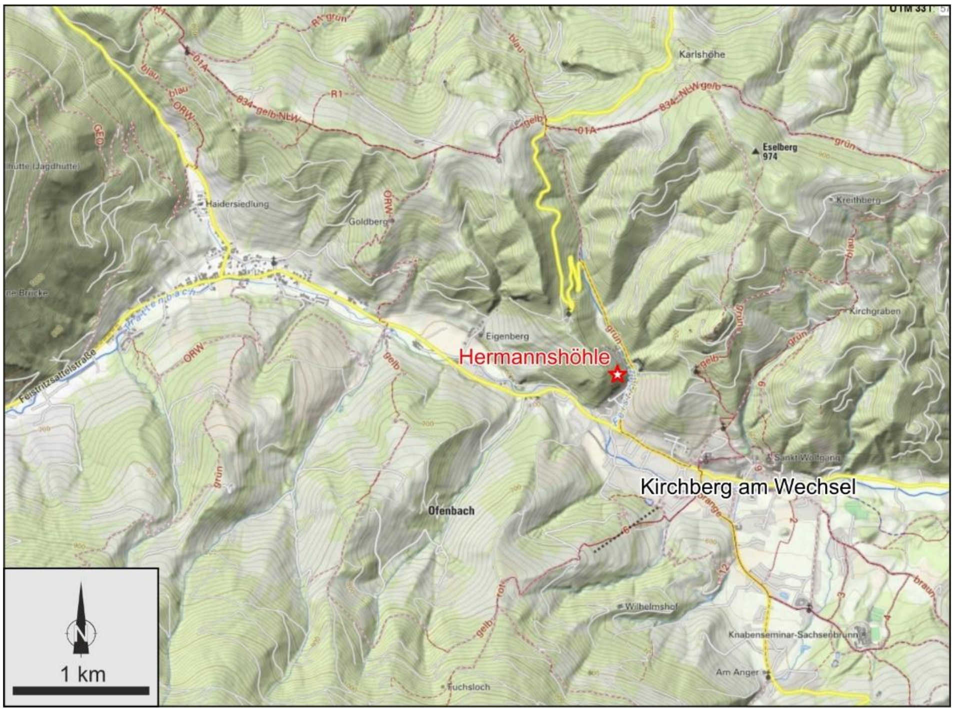

Fig. 64. Topographic map with the location of Hermannshöhle.

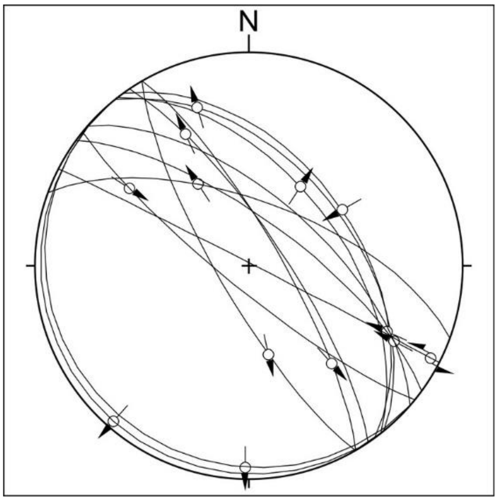

Fig. 65. Angelier diagram of fault-slip data (Equal-area stereographic projection, lower hemisphere) of reactivated faults from Hermannshöhle.

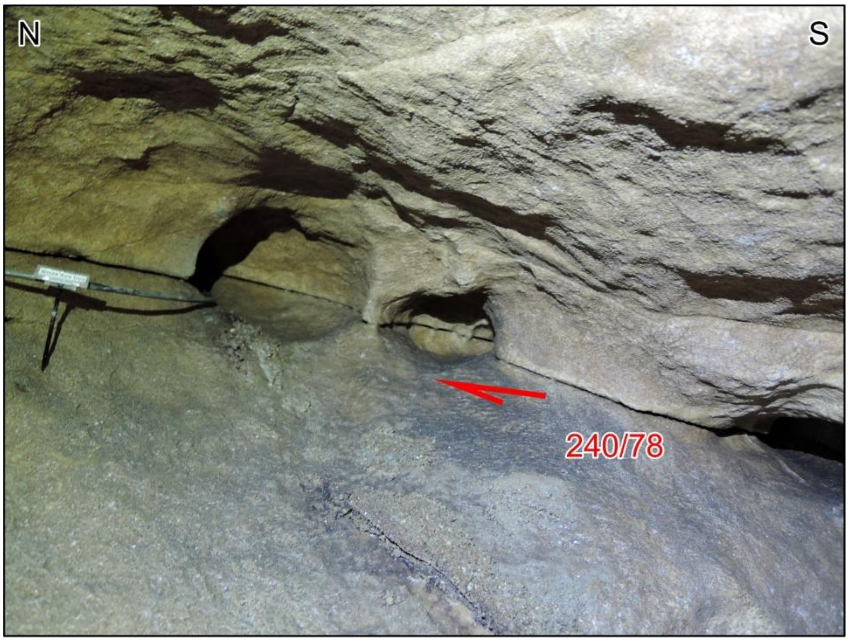

Fig. 66. Photograph of the reactivated fault in Hermannshöhle. upward view

24 Excentriqueshöhle

No. of Austrian cave register:	2872/4	*Province*	Lower Austria
Location	Bucklige Welt,<br>S margin of Vienna Basin	*Elevation of entrance [m a.s.l.]*	350
UTM 33T Easting	591,473	*UTM 33T Northing*	5,284,584
Length [m]	212	*Depth [m]*	11
Lithology	Limestone marble (Mid. Triassic)	*Key reference*	Fink et al., 1979

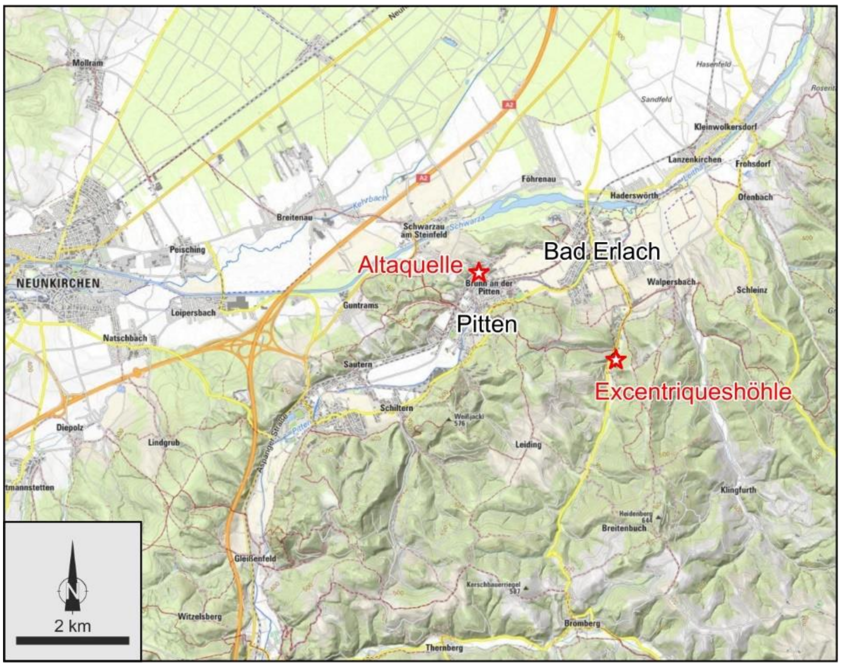

Fig. 67. Topographic map with the location of Excentriqueshöhle and Altaquelle.

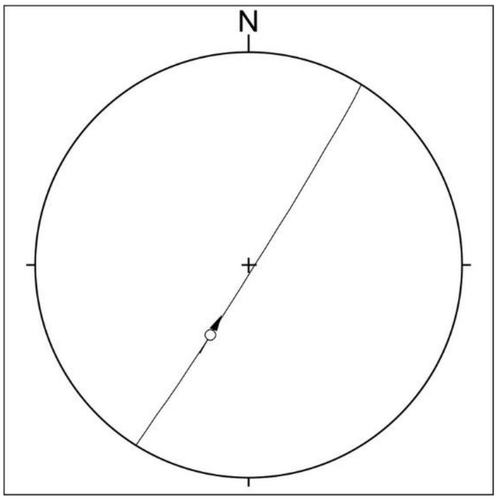

Fig.68. Angelier diagram of fault-slip data (Equal-area stereographic projection, lower hemisphere) of reactivated faults from Excentriqueshöhle.

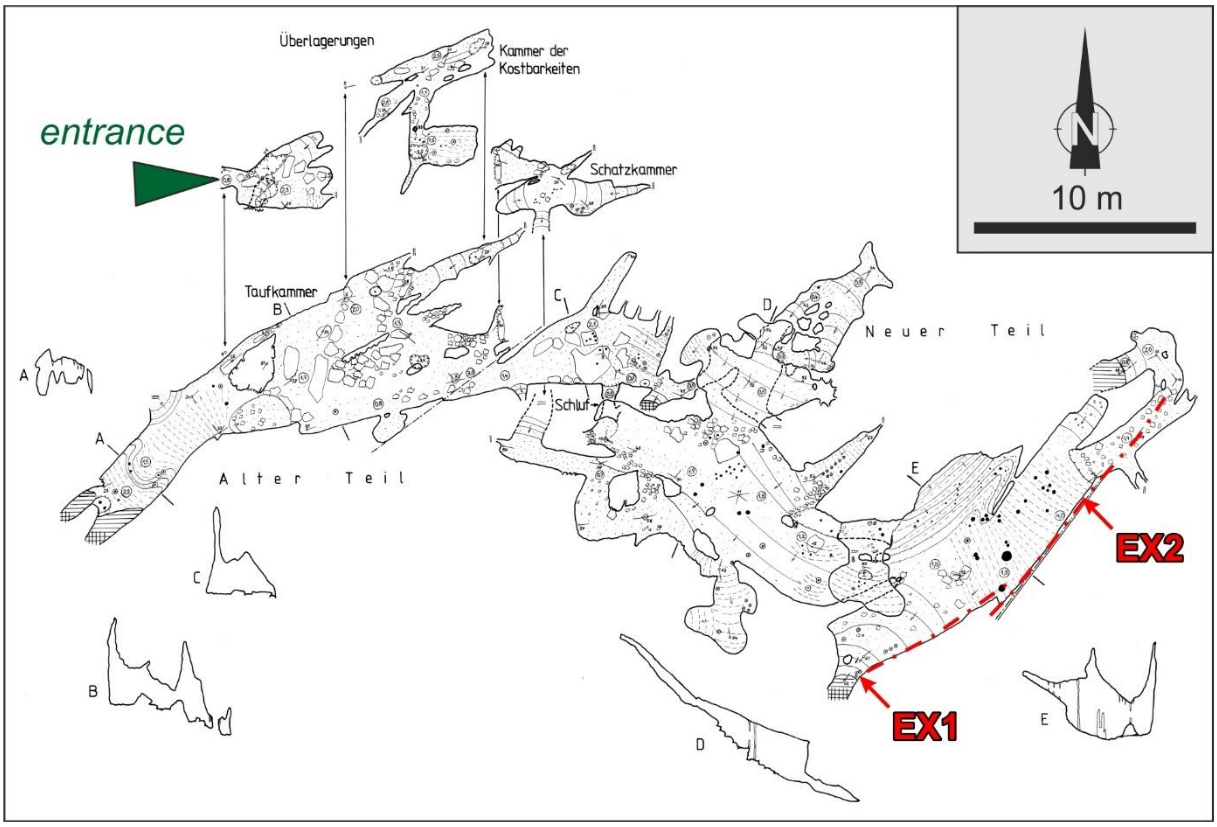

Fig. 69. Map of Excentriqueshöhle with sample locations. Map by: Winkler, G. (1985).

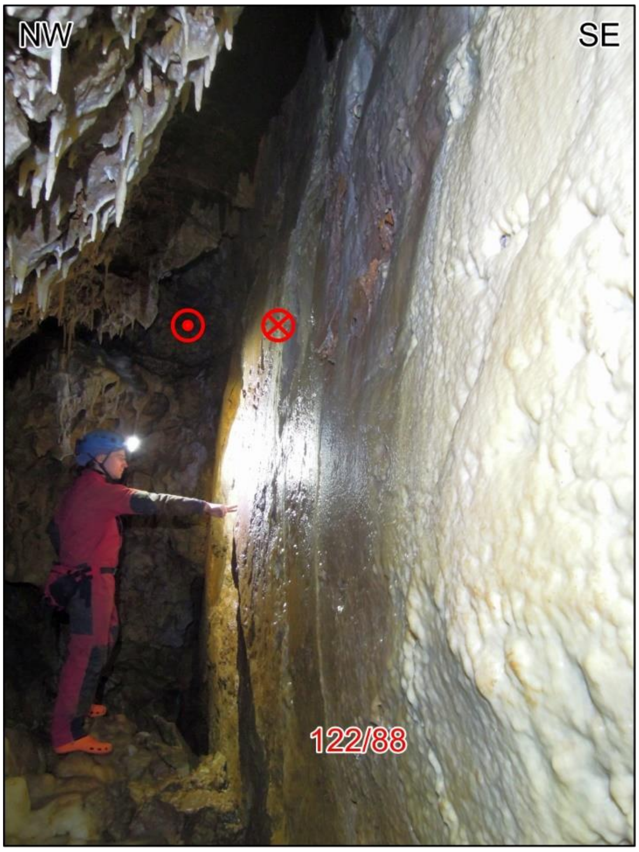

Fig. 70. Photograph of reactivated fault in Excentriqueshöhle.

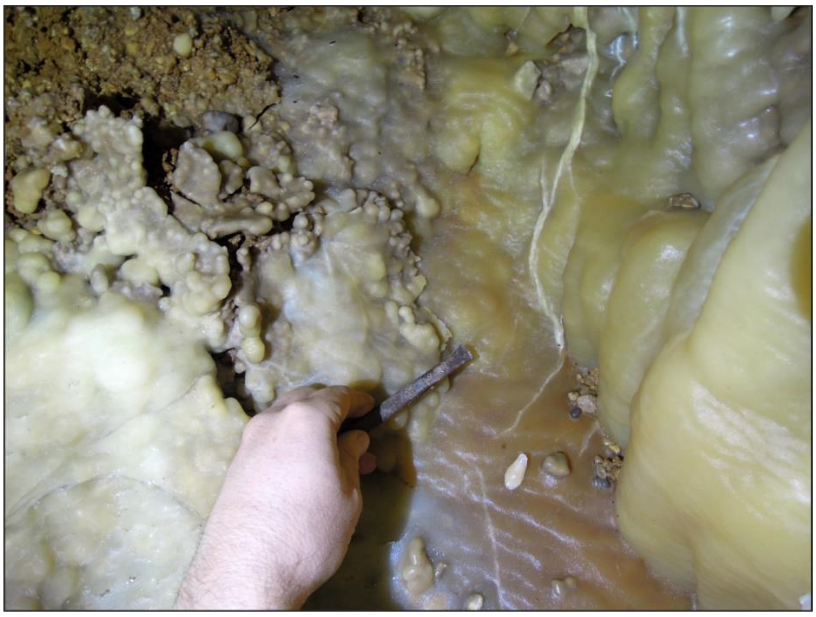

Fig. 71. Photograph of the faults in Excentriqueshöhle. Location of sample EX1.

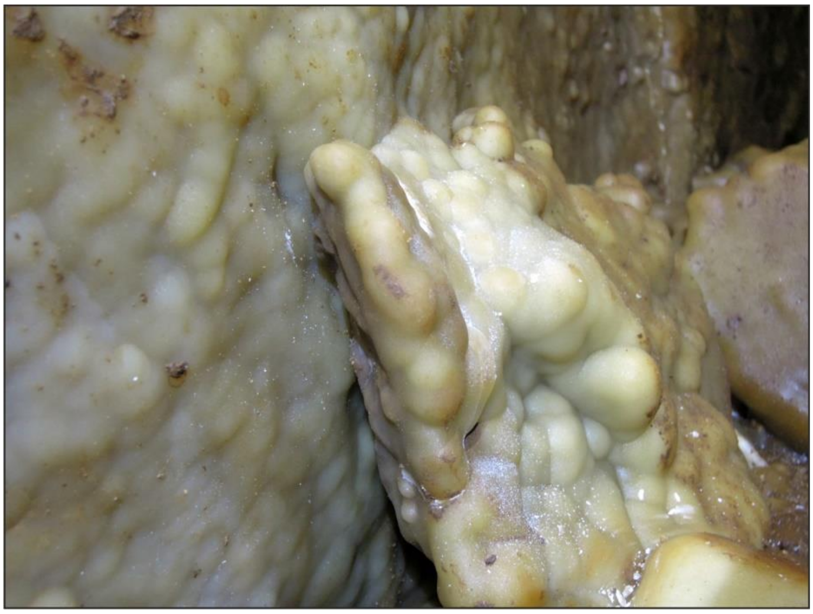

Fig. 72. Location of sample EX2. Width of the image is 10 cm..

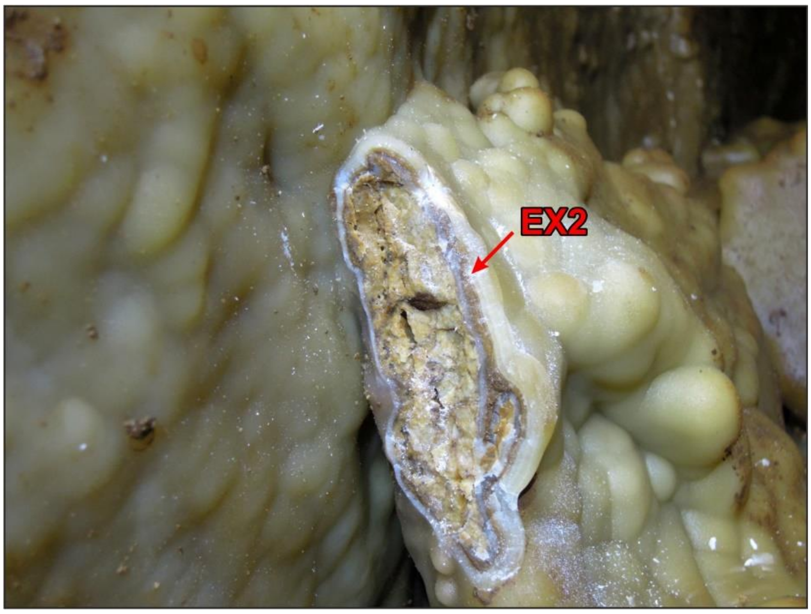

Fig. 73. Exact position of sub-sample extraction of EX2. Width of the image is 7 cm.

25 Altaquelle

No. of Austrian cave register:	2871/1	*Province*	Lower Austria
Location	Brunn bei Pitten,<br>S margin of Vienna Basin	*Elevation of main entrance [m a.s.l.]*	320
UTM 33T Easting	588,960	*UTM 33T Northing*	5,286,391
Length [m]	255	*Depth [m]*	13
Lithology	Wurstmarmor (Neogene)	*Key reference*	Fink et al., 1979

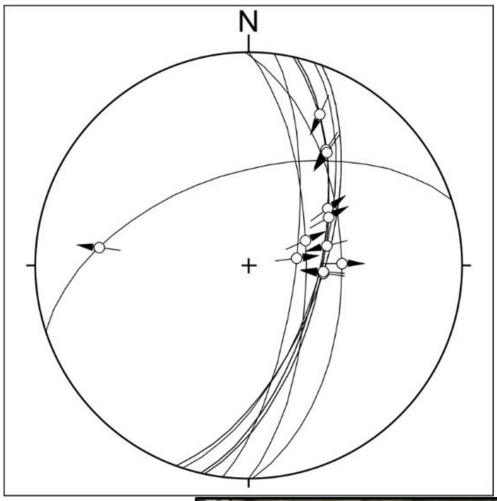

Fig. 74. Angelier diagram of fault-slip data (Equal-area stereographic projection, lower hemisphere) of reactivated faults from Altaquelle.

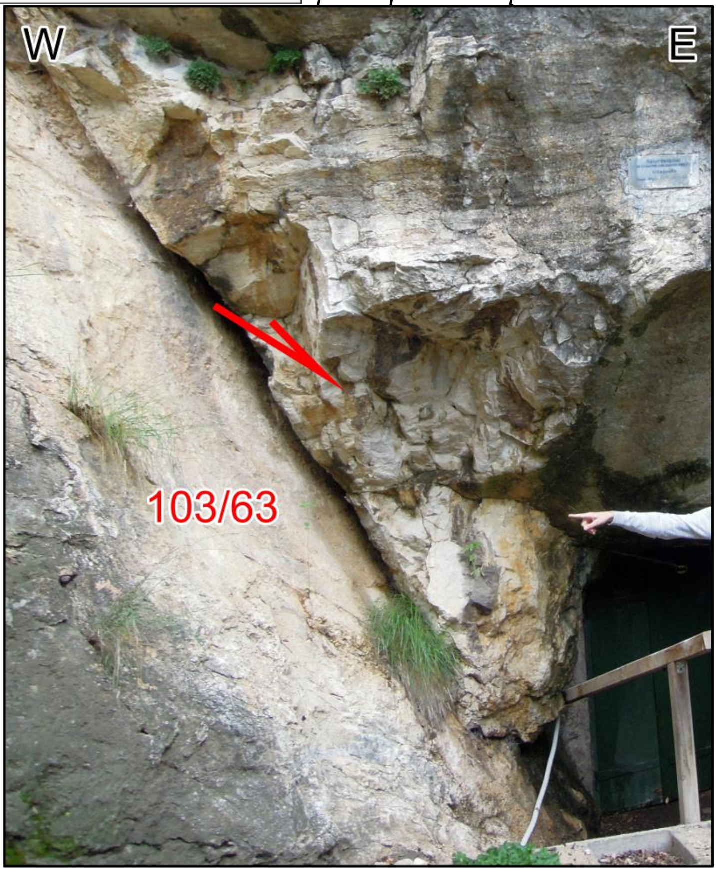

Fig. 75. Photograph of reactivated fault in Altaquelle.

26 Fraisloch

No. of Austrian cave register:	1864/6	*Province*	Lower Austria
Location	Fischauer Vorberge W, W margin of Vienna Basin	*Elevation of main entrance [m a.s.l.]*	465
UTM 33T Easting	583,298	*UTM 33T Northing*	5,296,604
Length [m]	333	*Depth [m]*	39
Lithology	Wurstmarmor (Neogene)	*Key reference*	Fink et al., 1979

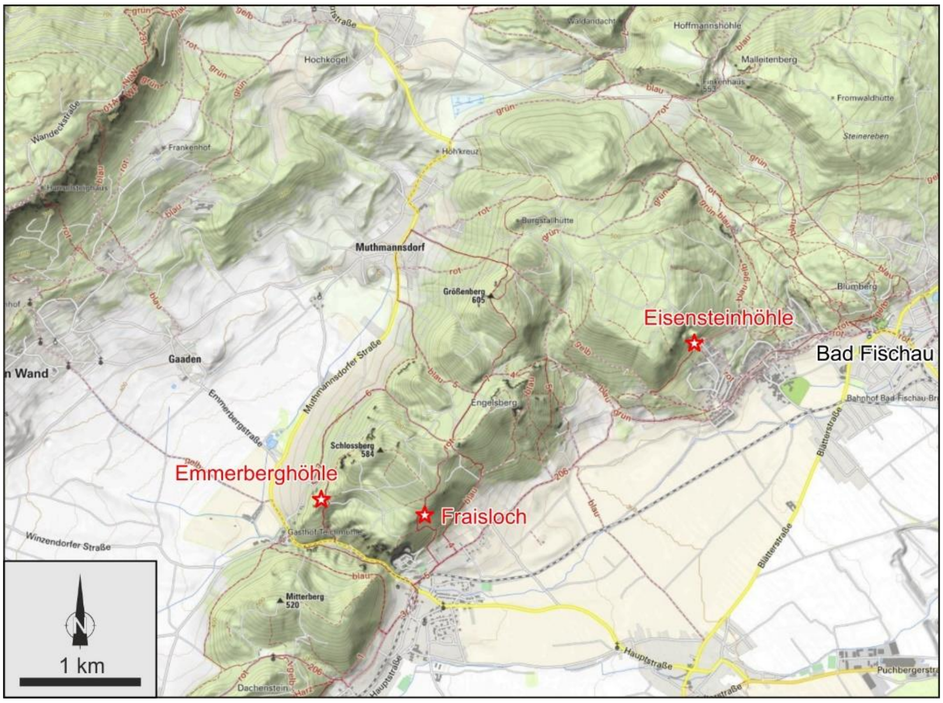

Fig.76. Topographic map with the location of caves in Fischauer Vorberge.

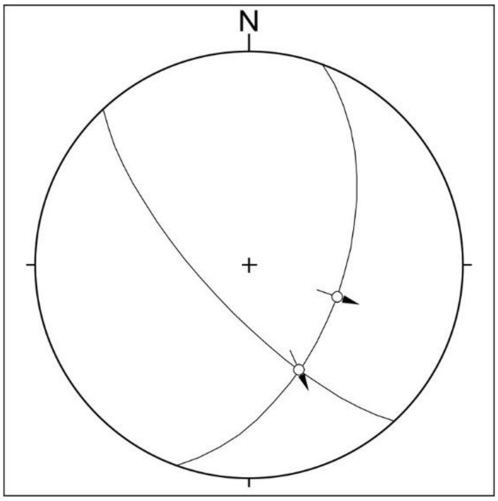

Fig. 77. Angelier diagram of fault-slip data (Equal-area stereographic projection, lower hemisphere) of reactivated faults from Fraisloch.

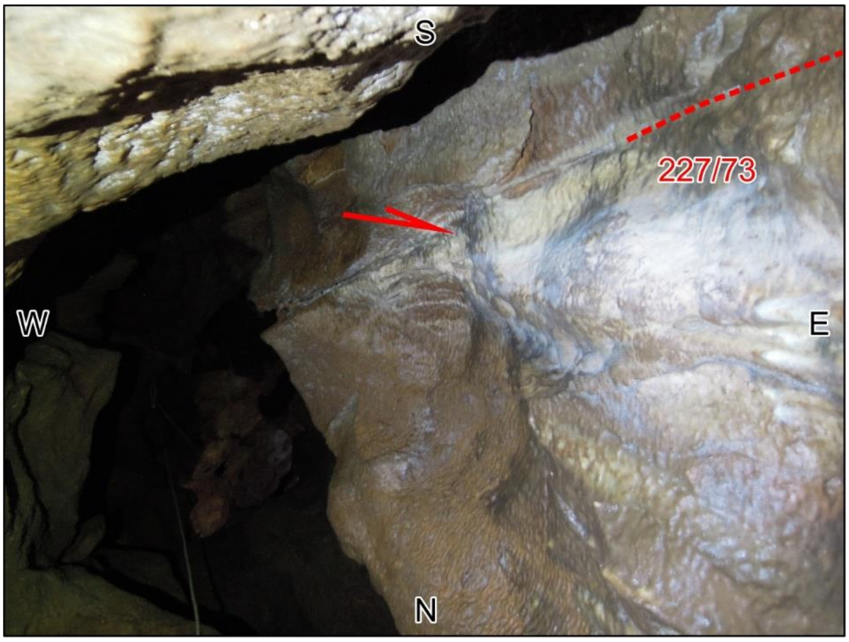

Fig. 78. Photograph of the reactivated fault in Fraisloch. oblique upward view

27 Eisensteinhöhle

No. of Austrian cave register:	1864/1	*Province*	Lower Austria
Location	E of Fischauer Vorberge, W margin of Vienna Basin	*Elevation of main entrance [m a.s.l.]*	430
UTM 33T Easting	585,576	*UTM 33T Northing*	5,298,089
Length [m]	2,341	*Depth [m]*	87
Lithology	Wurstmarmor (Neogene)	*Key reference*	Hardege et al., 2019

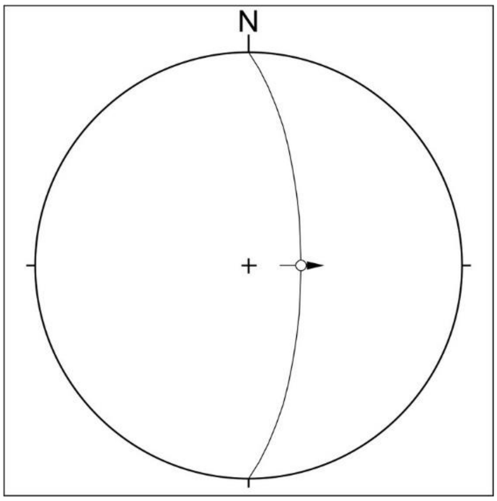

Fig.79. Angelier diagram of fault-slip data (Equal-area stereographic projection, lower hemisphere) of reactivated faults from Fraisloch.

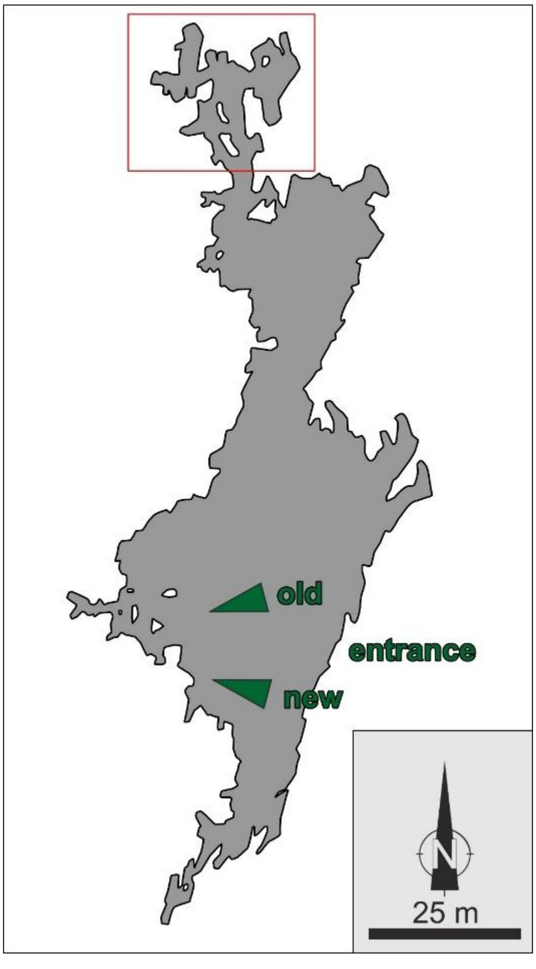

Fig. 80. Overview map of Eisensteinhöhle. Map by: Plan, L. (1999).

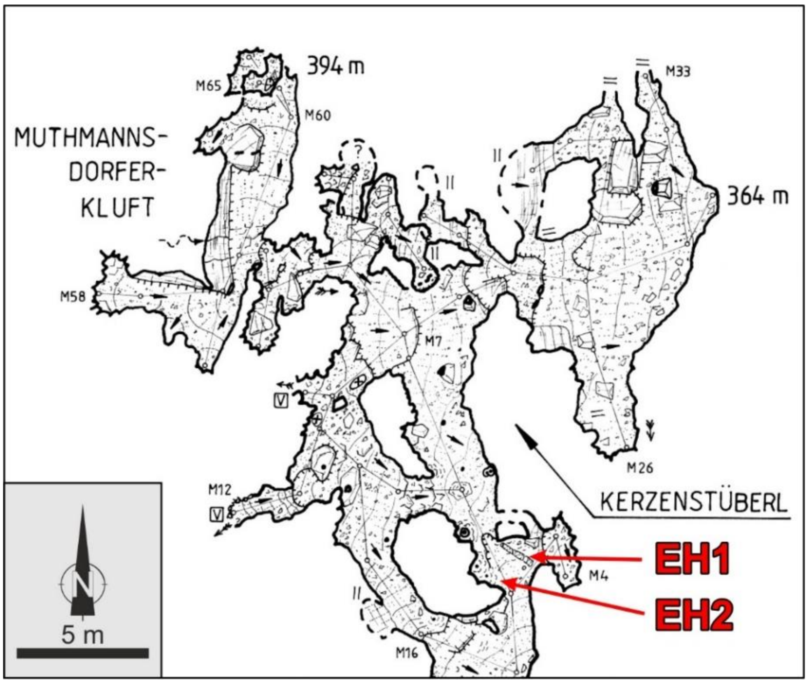

Fig. 81. Detailed map of Eisensteinhöhle with locations of sample EH1 and EH2. Map by Plan, L. (1999).

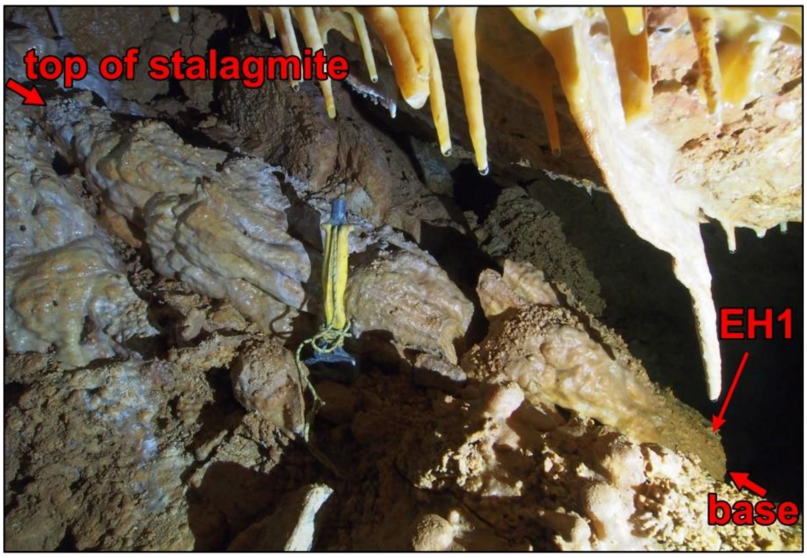

Fig. 82. Photo of the sample locations in Eisensteinhöhle. Sample EH1 was taken at the base of a 1.2 m-long fallen stalagmite.

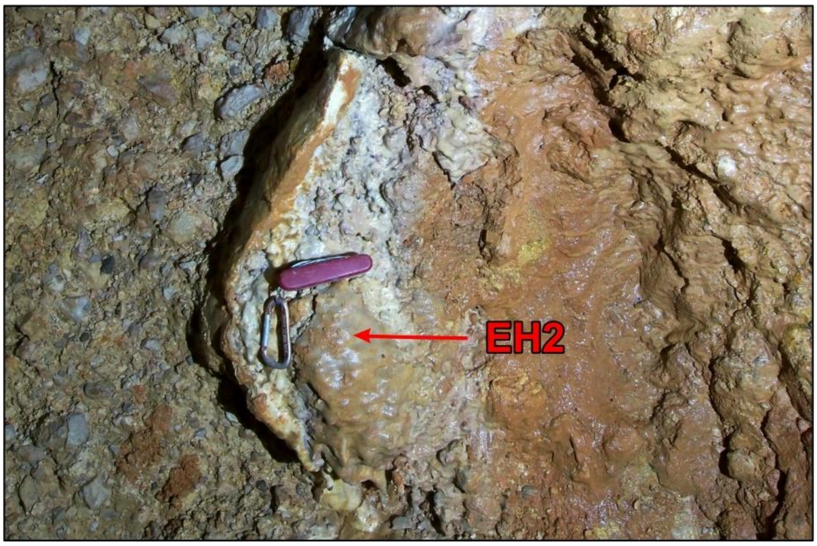

Fig. 83. Sample EH2 was taken from a small stalagmite that grew on screen which is related to a collapse event. 7 cm-long pocketknife for scale.

28 Emmerberghöhle

No. of Austrian cave register:	1864/3	*Province*	Lower Austria
Location	S of Fischauer Vorberge, W margin of Vienna Basin	*Elevation of main entrance [m a.s.l.]*	430
UTM 33T Easting	582,412	*UTM 33T Northing*	5,296,747
Length [m]	150	*Depth [m]*	23
Lithology	Wettersteinkalk (Middle Triassic)	*Key reference*	Fink et al., 1979

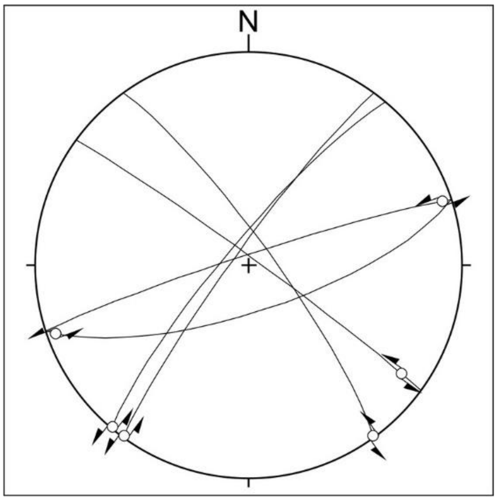

Fig. 84. Angelier diagram of fault-slip data (Equal-area stereographic projection, lower hemisphere) of reactivated faults from Emmerberghöhle.

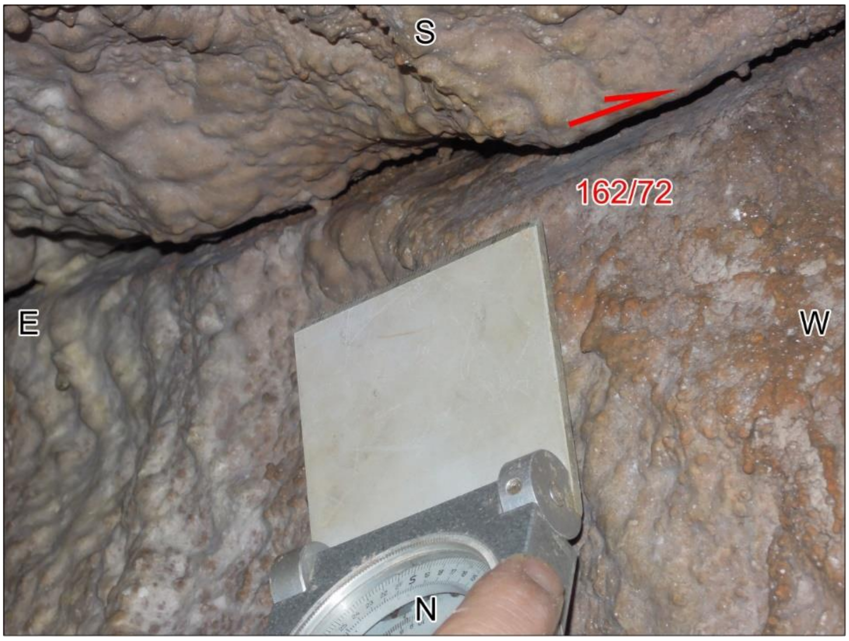

Fig. 85. Vertical upward photograph of an active fault in Emmerberghöhle.

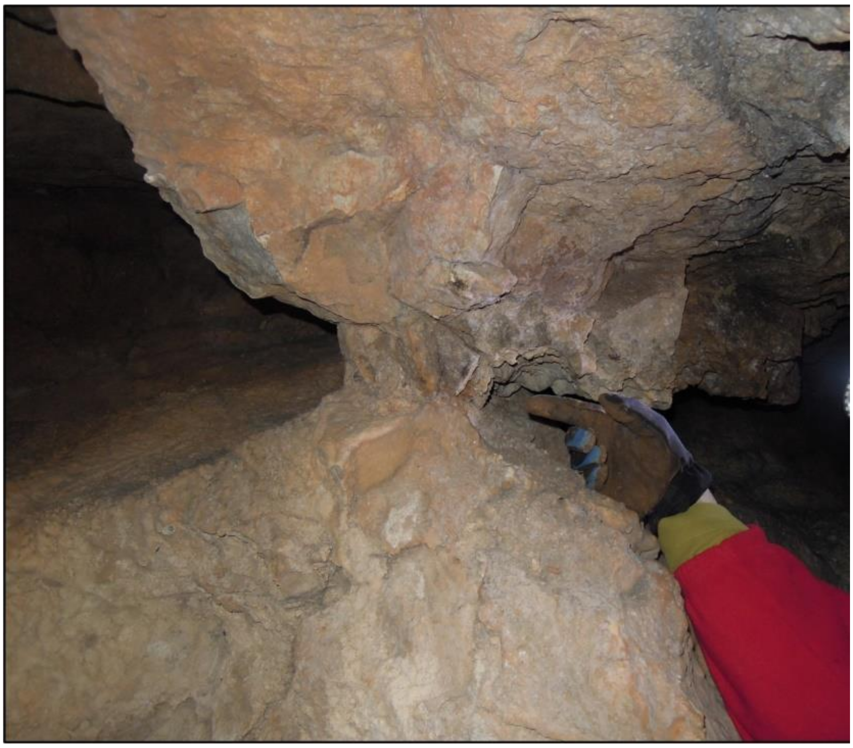

Fig. 86. Photo of the fault in Emmerberghöhle with samples´ location in upward view.

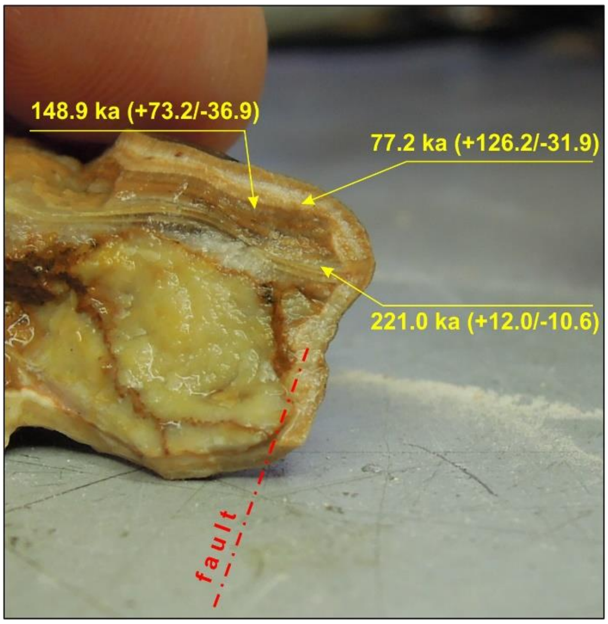

Fig.87. Detail of flowstone sample EM1 (including dating result). The width of the picture is 5 cm.

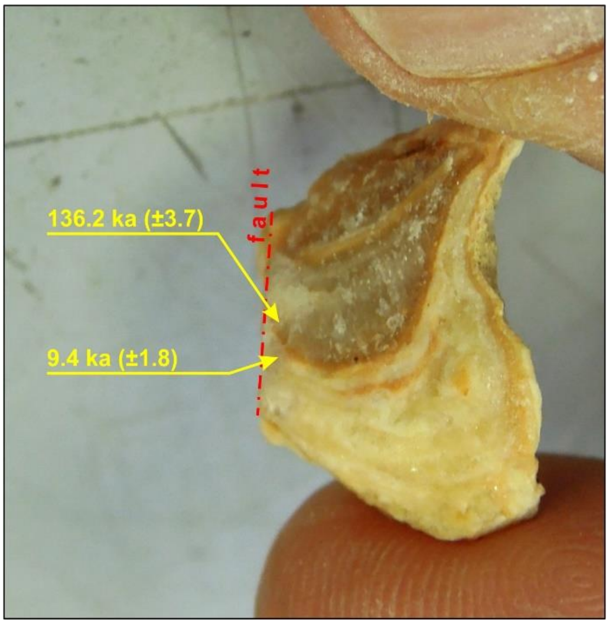

Fig. 88. Detail of Sample EM2 (including dating result). The width of the picture is 4 cm.

References

Behm, M., Plan, L., Seebacher, R. and Buchegger, G. (2016): Dachstein. – In: Spötl, C., Plan, L. & Christian, E. (Ed.): Höhlen und Karst in Österreich. – Linz (OÖ-Landesmuseum): 569-588.

Fink, M.H., Hartmann, H. and Hartmann W. (1979): Die Höhlen Niederösterreichs, Band 1. – Die Höhle, Supplement 28.

Golicz, M. (2021): Die Gamssteighöhle im Hohen Göll (Salzburg). – Die Höhle, 72: 32-44.

Hardege, J.; Plan, L.; Winkler, G.; Grasemann, B.; Baroň, I. (2019): Is hydrotectonics influencing the thermal spring in Eisensteinhöhle (Bad Fischau, Lower Austria)? - Austrian J. Earth Sci., 112: 166-181. DOI: 10.17738/ajes.2019.0009.

Klappacher, W.; Haseke-Knapczyk, H. (1985): Salzburger Höhlenbuch, Band 4. – Salzburg (Landesverein für Höhlenkunde).

Klappacher, W.; Knapczyk, H. (1979): Salzburger Höhlenbuch, Band 3. – Salzburg (Landesverein für Höhlenkunde).

Klappacher, W.; Kondratowicz, R.; Dokupil, W.; Golicz, M.; Rysiecki, Z.; Wierzbowski, M. (2009): Polnische Forschungen in den Höhlen Salzburgs - Teil 2: Drei Expeditionsgruppen dokumentieren ihre Erfolge am Hohen Göll, im Hagengebirge und im Tennengebirge. – Die Höhle 60:44-58.

Oberender, P.; Bauer, H.; Nagl, M. (2017): (Neu-)Vermessung der Höhlen am Offenberg (1733 - Troiseck). – Höhlenkundliche Mitteilungen, Wien, 73: 60-66.

Plan, L., Schober, A., Scholz, D., Spötl, C., Pruner, P. & Bosák, P. (2015): Speleogenesis of the Hermannshöhle cave system (Austria): Constraints from 230Th/ U-dating and palaeomagnetic analysis. - Int. J. Speleology, 44: 315-326; DOI: 10.5038/1827-806X.44.3.8.

Plan, L.; Baroň, I. (2021): Der Potentialschacht im Hochschwab (1744/475). – Höhlenkundliche Mitteilungen (Wien), 77: 137 – 147.

Plan, L.; Guggenberger, E.; Baroň, I. and Koltrai, G. (2021): Die Speikbodenhöhle am Hochschwab (1744/650). – Höhlenkundliche Mitteilungen (Wien), 77: 101-109.

Plan, L.; Kaminsky, E.; Racine, T.; Koltai, G. (2021): Genetische Interpretation der Eisriesenwelt (Tennengebirge). – Die Höhle, 72, 117-138.

Plan, L.; Oberender, P.; Funk, B.; Muttenthaler, A.; Nagl, M. (2017): Forschungen im Bereich Sonnschien- und Häuselalm (1744 Hochschwab, Stmk.). - Höhlenkundliche Mitteilungen, Wien, 73: 115-126.

Plan, L.; Spötl, Ch.; Bryda, G. (2019): Speläologie und Geologie der Hirschgrubenhöhle am Hochschwab (Steiermark). – Die Höhle, 70: 79-93.

Pointner, P. (2016): Tennengebirge. – In: Spötl, C., Plan, L. & Christian, E. (Ed.): Höhlen und Karst in Österreich. – Linz (OÖ-Landesmuseum): 553-568.

Seebacher, R. (2015a): Tauchgänge in der Kugelmühle bei Altaussee, Kat.Nr. 1623/221, Totes Gebirge, Stmk. – Mitteilungen Verein für Höhlenkunde Obersteier, 32: 136-139.

Seebacher, R. (2015b): Forschungen im Zuge des Projektes Tauplitzalm, Totes Gebirge, Gemeinde Bad Mitterndorf, Stmk. – Mitteilungen Verein für Höhlenkunde Obersteier, 32: 126-131.

Wierzbowski, M. (2021): Weiterforschung in der Interessanten Höhle im Hagengebirge (Salzburg). – Die Höhle, 72: 22-31.

Fault-Slip Data and paleostress reconstruction results

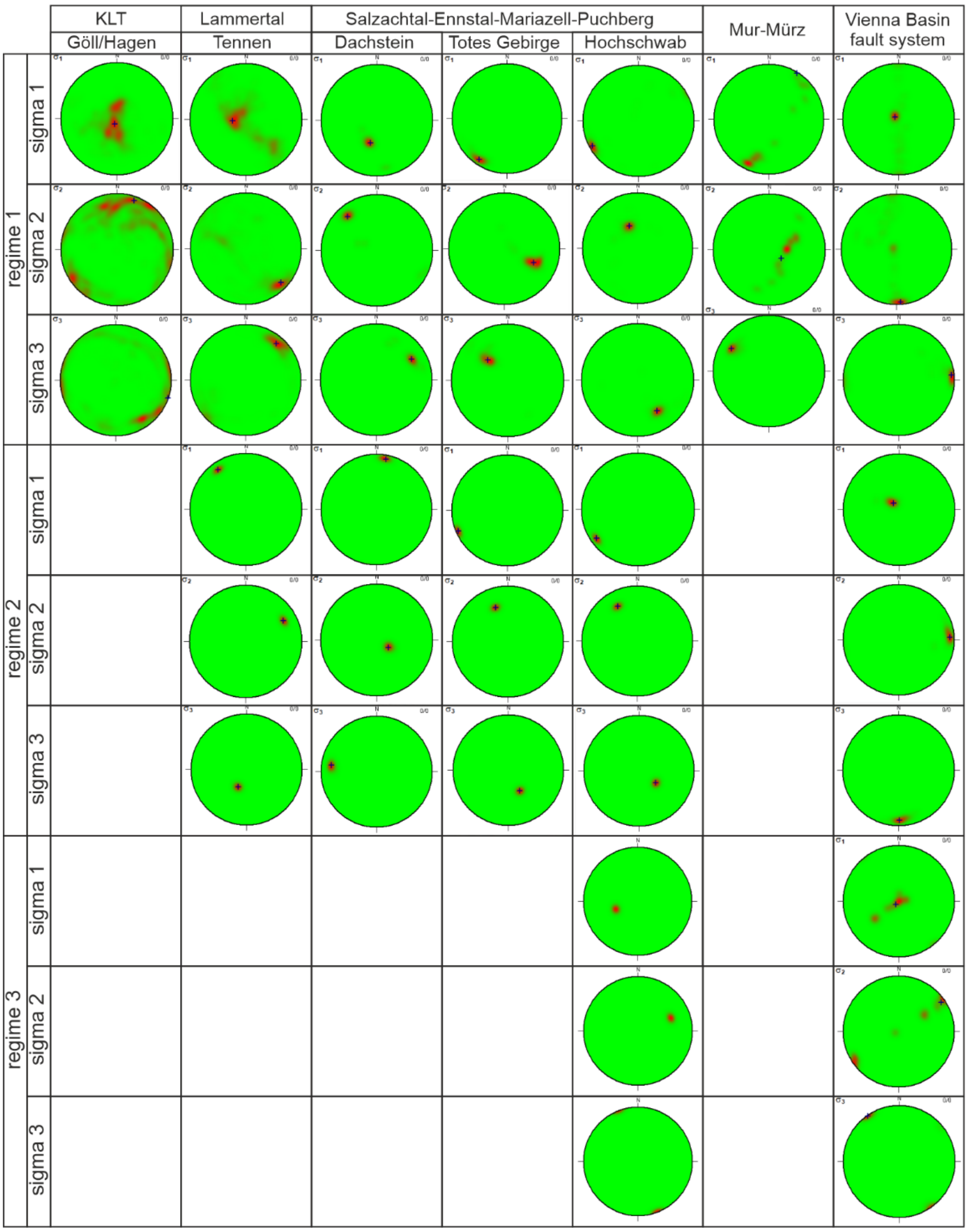

Fig. 83. Density plots of computed stress tensors

Table S1. Fault-slip data from the studied caves in the Northern Calcareous Alps

Major fault	Area	Cave	Fault plane		Displacement vector		Kinematics	Offset [cm]
			Dip dir.	Dip ang.	Trend	Plunge		
KLT	Hoher Goll	1. Gruberhorn	196	22	196	22	N	4
			300	60	4	38	D	20
			170	20	170	20	N	7
			2	65	275	6	D	10
			200	32	158	25	D	3
			222	31	201	29	S	7
			195	35	195	35	N	4
			190	30	190	30	N	4
			107	85	107	85	N	25
			203	30	227	28	S	4
			192	55	229	49	S	3
		3. Dependance	208	55	313	45	SR	15
			218	50	230	49	S	7
		2. Gammsteig	184	46	204	44	S	15
			138	38	138	38	N	2
			210	16	210	16	N	7
			188	25	188	25	N	5
			208	40	226	39	S	12
			285	80	285	80	N	5
			190	25	190	25	N	12
			210	33	210	33	N	6
			76	76	157	30	D	5
			280	84	3	49	D	17
			24	35	358	32	S	12
			80	68	131	57	D	17
	Hagengebirge	5. Tanatal	108	35	158	24	D	26
		4. Interesante	42	45	42	45	R	6
			68	65	105	55	D	20
Lammertal	Tennnegebirge	10. Jack'Daniels	181	58	155	56	S	15
			270	67	292	65	D	5
			272	73	340	51	D	22
			191	56	142	44	S	20
			80	27	122	21	S	12
			333	52	33	33	D	2
		8. Schneelock	140	80	155	80	S	40
		9. Felsbrückenhöhle	76	75	50	20	S	7
			94	27	27	10	S	21
			86	26	49	19	S	20
			126	36	24	12	S	30
			96	29	46	25	S	20
			72	24	41	21	S	30
			66	26	45	20	S	43
			102	30	76	30	S	23
		7. Bergerhöhle	80	40	40	30	S	3
			250	55	250	55	R	3
			334	88	238	77	N	2
			100	65	28	35	S	25
			345	45	345	45	R	3
			66	87	35	20	S	5
		6. Bierloch	204	54	138	11	D	7
			200	65	270	29	D	4
			215	57	251	47	D	11
			201	45	124	47	D	12
		11. Eisrisenwelt	301	70	231	42	S	10
			280	43	196	5	S	10
			272	55	187	7	S	10

		11. Eisrisenwelt	120	44	63	38	S	10
			38	45	38	45	N	10
			56	43	56	43	N	10
KLT/SEMP	Dachstein	12. Hirlatz	10	56	44	60	RS	7
		13. Mammuthhöhle	103	83	13	7	S	
			81	63	81	63	N	30
			9	28	321	17	S,R	2
			2	28	321	16	S,R	
			11	36	317	24	S,R	
			24	32	319	20	S,R	
			21	32	320	19	S,R	
			146	63	59	14		
			140	86	230	3		
			25	26	30	25	R	
			30	31	32	30	R	
			140	85	229	10	S	
			135	82	229	10	S	
			26	28	30	28	R	
			280	68	290	67	N	
			275	65	265	64	N	
			275	65	186	2	S	
			281	64	265	64	N	80
			281	64	196	11	S	
			140	85	229	10	S	
			135	82	229	10	S	8
	Totes Gebirge	15. Bullenhöhle	243	77	250	76		
			275	59	200	23		
			275	59	205	45		
			226	50	221	49	R	
			272	89	182	23	D	
			63	68	347	23	R	
			63	68	63	68	R	
			106	56	17	9	D	1
			70	77	340	9	D	1
			214	72	158	63	R	10
			106	50	16	9	D	2
		14. Kugelmühlehöhle	265	74	174	2	N	10
			255	68	181	32	N	10
			273	71	185	6	S	10
			115	31	200	4	N	10
			154	55	234	15	S	10
			321	65	235	10	S	10
			140	88	214	25		10
			302	68	236	21		10
VBT	Margins of Vienna Basin	27. Eisensteinhöhle	90	70	90	65	N	20
		26. Fraisloch	227	73	154	45	N, S	2
			110	54	110	54	N	2
		28. Emmerberghöhle	162	72	252	5	S	4
			342	86	72	5	S	4
			36	87	126	13	S	1
			310	81	220	1	S	
			54	81	144	1	S	
			306	84	216	1	S	
		25. Altaqelle	342	54	277	30	N	2
			89	54	89	54	N	10
			97	72	83	70	N	
			90	68	69	63	N	
			103	63	53	53	N	
			102	62	59	54	N	
		24. Excentriqueshöhle	122	88	221	59	S	10
		23. Hermannshöhle	240	78	168	55	S	1
			20	65	324	55	N	1
			221	8	181	7	N	1
			52	55	33	53	N	1
			51	50	344	21	N	1
			60	78	140	40	N	
			56	77	330	33	S	1
			219	81	304	34	S	1
			54	48	59	48	S	1
			36	72	126	32	S	1

			41	63	135	32	S	1
			230	4	221	3	S	1
			27	89	117	5	S	1
MM		22. Räuberhöhle	32	42	302	5	D	30
			11	51	45	36	R	
			28	57	300	25	D	5
			183	59	106	26	D	1
			216	73	295	27	D	10
		21. Zederhaushöhle	162	89	72	5	S	1
			162	54	77	7	S	1
			105	81	198	14	D	4
			336	86	24	45	S	1
		20. Gr.Offenbergerhöhle	341	74	267	40	S	1
			121	50	118	42	S	1
			117	86	206	25	N	1
SEMP	Hochschwab	17. Speikbodenhöhle	171	39	117	26	S, N	2
			207	46	153	33	S, N	2
			180	35	165	35	S, N	3
			180	35	224	35	R	1
			183	34	173	33	S, N	1
			196	55	198	50		2
			196	55	106	7	S, N	3
			190	87	100	7	S	2
			70	34	76	32	R	1
			79	34	99	27	N	1
			162	36	162	36	N	1
			104	41	168	13	N	2
			104	41	55	30	R	8
			212	76	249	67	N	1
			212	76	249	67	N	1
			79	33	79	33	N	
		18. Potentialschacht	118	7	136	7	R	1
			144	30	130	29	R	
			318	64	341	63		
			162	30	150	21	R	
			130	25	205	17	R	1
			112	36	37	7	R, D	1
			147	38	142	38	R	
			214	40	206	40		
			301	85	211	5	S	18
			125	85	35	12	S	18
			127	85	37	16	S	18
		16. Pol_Nord_Ponor	155	63	83	41	N, S	2
			164	55	124	52	N, S	1
			166	37	124	36	N	1
		19. Hirschgrubenhöhle	350	87	261	22	N, S	25*

for abbreviated major faults names and caves location see main text

* Plan et al. 2010

N-normal R-reverse D-dextral S-sinistral

Table S2. Results of paleostress analysis

Area	regime	Regime	Sig 1		Sig 2		Sig 3		Lode
			Trend	Plunge	Trend	Plunge	Trend	Plunge	
KLT - Hagengebirge/Göll	1	Extension	201	82	19	8	109	0	-0.46
Lammertal - Tennengebirge	1	Extension	263	70	134	13	41	15	0.80
	2	Strike-Slip	325	15	62	24	206	61	-0.13
SEMP-Dachstein	1	Oblique extension	196	55	319	21	60	27	0.29
	2	Strike-slip/Obl. Compression	11	8	124	69	278	19	0.13
SEMP - Totes Gebirge	1	Oblique Compression	214	10	116	40	316	48	0.56
	2	Strike-slip	247	4	340	35	151	54	0.41
SEMP-Hochschwab	1	Oblique Compression	242	6	340	55	148	35	0.45
	2	Oblique Compression	235	11	331	29	125	59	0.20
	3	Oblique extension	247	58	67	36	151	0	0.35
MM	1	Strike-slip/Obl. Compression	32	2	126	67	301	23	0.96
VBT	1	Extension	302	84	175	4	85	5	0.45
	2	Extension	319	77	87	8	179	10	-0.36
	3	Extension	221	83	56	7	325	2	0.59
